# Random Unitary Representations of Surface Groups I: Asymptotic Expansions

**DOI:** 10.1007/s00220-021-04295-5

**Published:** 2021-12-31

**Authors:** Michael Magee

**Affiliations:** Department of Mathematical Sciences, Durham University, Lower Mountjoy, DH1 3LE Durham, UK

## Abstract

In this paper, we study random representations of fundamental groups of surfaces into special unitary groups. The random model we use is based on a symplectic form on moduli space due to Atiyah, Bott and Goldman. Let $\Sigma _{g}$ denote a topological surface of genus $g\ge 2$. We establish the existence of a large *n* asymptotic expansion, to any fixed order, for the expected value of the trace of any fixed element of $\pi _{1}(\Sigma _{g})$ under a random representation of $\pi _{1}(\Sigma _{g})$ into $\mathsf {SU}(n)$. Each such expected value involves a contribution from all irreducible representations of $\mathsf {SU}(n)$. The main technical contribution of the paper is effective analytic control of the entire contribution from irreducible representations outside finite sets of carefully chosen rational families of representations.

## Introduction

Let $g\in \mathbf {N}$ with $g\ge 2$ and let $\Sigma _{g}$ denote a closed topological surface of genus *g*. If $x_{0}$ is a point in $\Sigma _{g}$, then we have

$$\begin{aligned} \pi _{1}(\Sigma _{g},x_{0})\cong \Gamma _{g}{\mathop {=}\limits ^{\mathrm {def}}}\langle a_{1},b_{1},\ldots ,a_{g},b_{g}\,|\,[a_{1},b_{1}]\cdots [a_{g},b_{g}]\rangle . \end{aligned}$$

The group $\Gamma _{g}$ is called the *surface group* of genus *g*. For $n\in \mathbf {N}$, the group $\mathsf {U}(n)$ is the group of $n\times n$ complex unitary matrices with respect to the standard Hermitian inner product on $\mathbf {C}^{n}$. Then $\mathsf {SU}(n)$ is the subgroup of $\mathsf {U}(n)$ consisting of matrices with unit determinant.

The space of homomorphisms $\mathrm {Hom}(\Gamma _{g},\mathsf {SU}(n))$ is given the topology coming from the embedding

1.1 $$\begin{aligned} \mathrm {Hom}(\Gamma _{g},\mathsf {SU}(n))\hookrightarrow \mathsf {SU}(n)^{2g},\quad \phi \mapsto (\phi (a_{1}),\phi (b_{1}),\ldots ,\phi (a_{g}),\phi (b_{g})). \end{aligned}$$

This embedding shows that $\mathrm {Hom}(\Gamma _{g},\mathsf {SU}(n))$ is an algebraic variety, but it is a variety with singularities [Gol84, pg. 204 Prop.]. We let $\mathrm {Hom}(\Gamma _{g},\mathsf {SU}(n))^{\mathrm {irr}}$ denote the collection of homomorphisms $\phi$ such that $\phi$ is irreducible as a linear representation of $\Gamma _{g}$. The space $\mathrm {Hom}(\Gamma _{g},\mathsf {SU}(n))^{\mathrm {irr}}$ then inherits the structure of a smooth non-complete manifold from (1.1) (*ibid.)*.

There is an action of $\mathsf {SU}(n)$ on $\mathrm {Hom}(\Gamma _{g},\mathsf {SU}(n))$ by postcomposition with inner automorphisms; from the point of view of (1.1), this is just the diagonal action of $\mathsf {SU}(n)$ by conjugation. This action factors through an action of $\mathsf {PSU}(n)$, that is, $\mathsf {SU}(n)$ modulo its finite center. The quotient by this action is denoted by $\mathrm {Hom}(\Gamma _{g},\mathsf {SU}(n))/\mathsf {PSU}(n)$. It is shown by Goldman in *(ibid.) *that the action of $\mathsf {PSU}(n)$ on $\mathrm {Hom}(\Gamma _{g},\mathsf {SU}(n))^{\mathrm {irr}}$ is free and the *moduli space*

$$\begin{aligned} \mathcal {M}_{g,n}{\mathop {=}\limits ^{\mathrm {def}}}\mathrm {Hom}(\Gamma _{g},\mathsf {SU}(n))^{\mathrm {irr}}/\mathsf {PSU}(n) \end{aligned}$$

is a smooth real manifold. This moduli space is the underlying set of *random representations *of $\Gamma _{g}$ discussed in this paper. By a theorem of Narasimhan and Seshadri [NS65], if a complex structure on $\Sigma _{g}$ is fixed, $\mathcal {M}_{g,n}$ corresponds via a natural map to a moduli space of stable holomorphic rank-*n* vector bundles on $\Sigma _{g}$.

To describe the law of the random representation, we need to recall some further results of Goldman. In *(ibid*.), Goldman shows that there is a natural symplectic form $\omega _{g,n}$ on $\mathcal {M}_{g,n}$, defined up to a scalar normalization that we fix in §§2.7. This symplectic form arose previously in the work of Atiyah and Bott [AB83]. It is analogous to the Weil–Petersson form on the Teichmüller space of complex structures on $\Sigma _{g}$ and is defined precisely in §§2.7 of this paper. The symplectic form $\omega _{g,n}$ yields a volume form

$$\begin{aligned} d\mathrm {Vol}_{\mathcal {M}_{g,n}}{\mathop {=}\limits ^{\mathrm {def}}}\frac{\wedge ^{\frac{1}{2}\dim \mathcal {M}_{g,n}}(\omega _{g,n}^{\mathrm {ABG}})}{(\frac{1}{2}\dim \mathcal {M}_{g,n})!}. \end{aligned}$$

The random representations in this paper are sampled according to this volume form, normalized to be a probability measure. We call the normalized measure the *Atiyah–Bott–Goldman *measure.

The statistics of random representations we are interested in come from functions on

$\mathrm {Hom}(\Gamma _{g},\mathsf {SU}(n))$ that are invariant under conjugation by $\mathsf {SU}(n)$. The natural functions to integrate on moduli spaces like $\mathcal {M}_{g,n}$ are *geometric functions* (as studied, e.g., by Mirzakhani [Mir07] in the Weil–Petersson context). These functions are also called *Wilson loops* in the theoretical physics literature.

We now fix a concrete instance of a family of geometric functions. For $g\in \mathsf {U}(n)$, let $\mathrm {tr} (g)$ denote the trace of *g* as an $n\times n$ matrix. Given any element $\gamma \in \Gamma _{g}$, we obtain a continuous function

$$\begin{aligned} \mathrm {tr} _{\gamma }:\mathrm {Hom}(\Gamma _{g},\mathsf {SU}(n))\rightarrow \mathbf {C},\quad \mathrm {tr} _{\gamma }(\phi ){\mathop {=}\limits ^{\mathrm {def}}}\mathrm {tr} (\phi (\gamma )). \end{aligned}$$

Clearly $\mathrm {tr} _{\gamma }$ is invariant under conjugation by $\mathsf {SU}(n)$ and hence yields a continuous bounded function that we give the same name

$$\begin{aligned} \mathrm {tr} _{\gamma }:\mathcal {M}_{g,n}\rightarrow \mathbf {C}. \end{aligned}$$

In this paper, we instigate a study of the expected value of $\mathrm {tr} _{\gamma }$, that is,

1.2 $$\begin{aligned} \mathbb {E}_{g,n}[\mathrm {tr} _{\gamma }]{\mathop {=}\limits ^{\mathrm {def}}}\frac{\int _{\mathcal {M}_{g,n}}\mathrm {tr} _{\gamma }\,d\mathrm {Vol}_{\mathcal {M}_{g,n}}}{\int _{\mathcal {M}_{g,n}}\,d\mathrm {Vol}_{\mathcal {M}_{g,n}}}. \end{aligned}$$

For fixed $\gamma$, we are interested in the large *n* behavior of this expected value. One has the simple bound

$$\begin{aligned} |\mathbb {E}_{g,n}[\mathrm {tr} _{\gamma }]|\le n \end{aligned}$$

and this bound is attained if $\gamma =\mathrm {id}_{\Gamma _{g}}$. On the other hand, if $\gamma \in \Gamma _{g}$ is not the identity, then a basic prediction is

1.3 $$\begin{aligned} \lim _{n\rightarrow \infty }\frac{|\mathbb {E}_{g,n}[\mathrm {tr} _{\gamma }]|}{n}=0. \end{aligned}$$

The significance of this prediction is that it extends a celebrated result of Voiculescu [Voi91, Theorem 3.8] on the asymptotic $*$-freeness of Haar unitary matrices, suitably interpreted, from free groups to surface groups. The current paper lays the groundwork for the proof of (1.3) in the next paper in the series [Mag21].

Not only that, but here we will expose a separate phenomenon for the values $\mathbb {E}_{g,n}[\mathrm {tr} _{\gamma }]$: they can be approximated to any order $O(n^{-M})$ as $n\rightarrow \infty$ by a Laurent polynomial in *n* depending on $\gamma$. The formal theorem is the following:

### Theorem 1.1

For any $g\ge 2$ and $\gamma \in \Gamma _{g}$, there is an infinite sequence of rational numbers

$$\begin{aligned} a_{-1}(\gamma ),a_{0}(\gamma ),a_{1}(\gamma ),a_{2}(\gamma ),\ldots \end{aligned}$$

such that for any $M\in \mathbf {N}$, as $n\rightarrow \infty$

1.4 $$\begin{aligned} \mathbb {E}_{g,n}[\mathrm {tr} _{\gamma }]=a_{-1}(\gamma )n+a_{0}(\gamma )+\frac{a_{1}(\gamma )}{n}+\cdots +\frac{a_{M-1}(\gamma )}{n^{M-1}}+O(n^{-M}). \end{aligned}$$

### Example 1.2

Let $\gamma \in \Gamma _{2}$ denote an element of the fundamental group of $\Sigma _{2}$ corresponding to a simple^1^ closed curve that separates the surface into two genus one surfaces with one boundary component each. In this case,

$$\begin{aligned} \mathbb {E}_{2,n}[\mathrm {tr} _{\gamma }]=\frac{2}{n}+\frac{5}{n^{3}}+O\left( \frac{1}{n^{5}}\right) \end{aligned}$$

as $n\rightarrow \infty$. This calculation is given in Appendix A.

### Remark 1.3

As Example 1.2 suggests, and as is known to be the case for the corresponding result about unitary representations of *free* groups [MP19a, Rmk. 1.9], it is natural to expect that all even coefficients $a_{2k}(\gamma )$ are zero, for any $\gamma$.

Theorem 1.1 has the following direct corollary.

### Corollary 1.4

For any $\gamma \in \Gamma _{g}$, the limit

$$\begin{aligned} \lim _{n\rightarrow \infty }\frac{\mathbb {E}_{g,n}[\mathrm {tr} _{\gamma }]}{n} \end{aligned}$$

exists.

The main technical result we prove in order to establish Theorem 1.1 is interesting in its own right so we discuss this now. The quantity in the denominator of (1.2), i.e., the symplectic volume of $\mathcal {M}_{g,n}$, was calculated non-rigorously by Witten in [Wit91]. Witten’s result is in terms of *Witten zeta functions*, so named by Zagier in [Zag94]. The *Witten zeta function* of $\mathsf {SU}(n)$ is defined by the series

1.5 $$\begin{aligned} \zeta (s;n){\mathop {=}\limits ^{\mathrm {def}}}\sum _{\begin{array}{c} {({\rho ,W)\in \widehat{\mathsf {SU}(n)}}} \end{array} }\frac{1}{(\dim W)^{s}}. \end{aligned}$$

Here $\widehat{\mathsf {SU}(n)}$ is the set of equivalence class of irreducible representations of $\mathsf {SU}(n)$. The sum in (1.5) converges absolutely for $\mathrm {Re}(s)>\frac{2}{n}$ by a result of Larsen and Lubotzky [LL08, Thm. 5.1] (see also [HS19, §2] for an alternative proof of this fact). The following formula that Witten obtained was rigorously established to hold by Sengupta in [Sen03].

### Theorem 1.5

(Witten’s formula). With the normalization of $\mathrm {Vol}_{\mathcal {M}_{g,n}}$ fixed as in §§2.7, we have

$$\begin{aligned} \int _{\mathcal {M}_{g,n}}d\mathrm {Vol}_{\mathcal {M}_{g,n}}=n\zeta (2g-2;n). \end{aligned}$$

In fact, Sengupta also provided a method to compute the integral of any continuous function on $\mathcal {M}_{g,n}$ with respect to $\mathrm {Vol}_{\mathcal {M}_{g,n}}$ and this is the starting point of our work. We let $\mathrm {\mathbf {F}} _{2g}{\mathop {=}\limits ^{\mathrm {def}}}\langle a_{1},b_{1},\ldots ,a_{g},b_{g}\rangle$ be the free group on the generators $a_{1},b_{1},\ldots ,a_{g},b_{g}$. Let $R_{g}{\mathop {=}\limits ^{\mathrm {def}}}[a_{1},b_{1}]\cdots [a_{g},b_{g}]\in \mathrm {\mathbf {F}} _{2g}$. Therefore, we have a surjective homomorphism $\mathrm {\mathbf {F}} _{2g}\xrightarrow {q_{g}}\Gamma _{g}$ obtained from quotient by the normal subgroup generated by $R_{g}$. We say that $w\in \mathrm {\mathbf {F}} _{2g}$ represents the conjugacy class of $\gamma \in \Gamma _{g}$ if $q_{g}(w)$ is conjugate to $\gamma$ in $\Gamma _{g}$. For any $w\in \mathrm {\mathbf {F}} _{2g}$, there is a *word map* $w:\mathsf {SU}(n)^{2g}\rightarrow \mathsf {SU}(n)$ obtained by substituting elements of $\mathsf {SU}(n)$ into the letters of *w*. We write $d\mu _{\mathsf {SU}(n)^{2g}}^{\mathrm {Haar}}(x)$ for the probability Haar measure on $\mathsf {SU}(n)^{2g}$, and this is the product of the probability Haar measures on the 2*g* factors.

One has the following corollary of Sengupta’s main result [Sen03, Thm.1].

### Corollary 1.6

Let $g\ge 2$ and $\gamma \in \Gamma _{g}$. Suppose that $w\in \mathrm {\mathbf {F}} _{2g}$ is an element representing the conjugacy class of $\gamma$. Then

1.6 $$\begin{aligned} \mathbb {E}_{g,n}[\mathrm {tr} _{\gamma }]=\zeta (2g-2;n)^{-1}\sum _{\begin{array}{c} {{({\rho ,W)\in \widehat{\mathsf {SU}(n)}}}} \end{array} }(\dim W){\mathcal {I}}(w,\rho ), \end{aligned}$$

where

1.7 $$\begin{aligned} {\mathcal {I}}(w,\rho ){\mathop {=}\limits ^{\mathrm {def}}}\int \mathrm {tr} (w(x))\overline{\mathrm {tr} (\rho (R_{g}(x)))}d\mu _{\mathsf {SU}(n)^{2g}}^{\mathrm {Haar}}(x) \end{aligned}$$

if the sum on the right-hand side of (1.6) is absolutely convergent.

We explain how to obtain Corollary 1.6 from [Sen03, Thm.1] in §§2.7 using ideas already presented in [Sen03]. Let $[\Gamma _{g},\Gamma _{g}]$ denote the commutator subgroup of $\Gamma _{g}$. Using Corollary 1.6, it is not hard to show:

### Proposition 1.7

If $\gamma \notin [\Gamma _{g},\Gamma _{g}]$, then there exists $n_{0}=n_{0}(\gamma )$ such that for $n\ge n_{0}$

$$\begin{aligned} \mathbb {E}_{g,n}[\mathrm {tr} _{\gamma }]=0. \end{aligned}$$

Proposition 1.7 is proved in §§3.2. This proves Theorem 1.1 in the case that $\gamma \notin [\Gamma _{g},\Gamma _{g}]$.

We now explain how we prove Theorem 1.1 in general by using Corollary 1.6. We first discuss the zeta function factor in (1.6). One has the following theorem due to Guralnick, Larsen and Manack.

### Theorem 1.8

([GLM12, Thm. 2]) For any $s>0$, $\lim _{n\rightarrow \infty }\zeta (s;n)=1$.

The limiting value arises from the trivial representation in (1.5); it is possible to boost the methods of [GLM12] to show that $\zeta (2g-2;n)$ can be approximated to any order $O(n^{-M})$ by a Laurent polynomial in *n*. In fact, the results of this paper can be viewed as a far generalization of this result and accordingly, the just-mentioned result is established as a byproduct of our proofs in §§5.4.

Thus, the proof of Theorem 1.1 amounts to showing that

1.8 $$\begin{aligned} \sum _{\begin{array}{c} {{({\rho ,W)\in \widehat{\mathsf {SU}(n)}}}} \end{array} }(\dim W){\mathcal {I}}(w,\rho ) \end{aligned}$$

is absolutely convergent and can be approximated to any order by some Laurent polynomial. The obvious bad feature of the sum (1.8) is that it runs over infinitely many representations of $\mathsf {SU}(n)$, and moreover, there are more and more of these as *n* increases. We aim to approximate (1.8) by finitely many of its terms and this requires an ordering of the representations of $\widehat{\mathsf {SU}(n)}$.

The correct way to do this is as follows. For every $k,\ell \in \mathbf {N}$ and pair of Young diagrams $\mu \vdash k$, $\nu \vdash \ell$, with number of rows given by $\ell (\mu )$, $\ell (\nu )$, for every $n\ge \ell (\mu )+\ell (\nu )$ there is a rational family of irreducible representations^2^ denoted by $(\rho _{n}^{[\mu ,\nu ]},W_{n}^{[\mu ,\nu ]})\in \widehat{\mathsf {SU}(n)}$ defined in §§2.4. If we define for $B\in \mathbf {N}$

1.9 $$\begin{aligned} \Omega (B;n){\mathop {=}\limits ^{\mathrm {def}}}\{\,(\rho _{n}^{[\mu ,\nu ]},W_{n}^{[\mu ,\nu ]}):\,\ell (\mu ),\ell (\nu )\le B,\mu _{1},\nu _{1}\le B^{2}\}, \end{aligned}$$

then $\Omega (B;n)$ is in one-to-one correspondence with the $(\mu ,\nu )$ such that $\ell (\mu ),\ell (\nu )\le B,\mu _{1},\nu _{1}\le B^{2}$ when *n* is sufficiently large. This specific choice of $\Omega (B;n)$ is for technical convenience, becoming useful in §§5.4.

We prove bounds on the ${\mathcal {I}}(w,\rho )$ in Theorem 4.1. The main challenges are that not only that estimates for ${\mathcal {I}}(w,\rho )$ must overcome the weights $\dim W$ in (1.8) but also that these bounds must remain effective for $\dim W$ much larger than *n*. It is quite well understood that matrix integrals such as ${\mathcal {I}}(w,\rho )$ are challenging in this regime as the main method of performing such integrals, known as the *Weingarten calculus*, often fails to produce understandable answers there. This is because the Weingarten function $\mathrm {Wg}_{n,k}$ defined in (2.12) becomes increasingly complicated for $k\gg n$, drawing on more and more different representations of large symmetric groups. We overcome this inherent difficulty as follows.

Firstly there is a minor observation that in all cases of interest, $\mathsf {SU}(n)$ can be replaced by $\mathsf {U}(n)$ in (1.7) (Proposition 3.1). The main idea is then that after some splitting up, parts of ${\mathcal {I}}(w,\rho )$ can be evaluated by integrating first $\overline{\mathrm {tr} (\rho (R_{g}(x)))}$ over all double cosets for a very large subgroup $\mathsf {U}(n-\mathfrak {D})\le \mathsf {U}(n)$ where $\mathfrak {D}$ is bounded depending only on *w*, which is fixed. During this first integration, the structure of the word $R_{g}$ can be exploited to produce a lot of cancelation.

After this initial integral, we then apply the Weingarten calculus through a novel strategy (cf. §§4.3) making heavy use of representation theory of both symmetric groups and $\mathsf {U}(n)$. What we achieve is the following technical result.

### Theorem 1.9

Suppose that $g\ge 2$, $w\in \mathrm {\mathbf {F}} _{2g}$ and $B\in \mathbf {N}$.

1. For $n\ge n_{0}(w,g)$, the sum in (1.6) is absolutely convergent.
2. As $$n\rightarrow \infty $$
  1.10 $$\begin{aligned} \sum _{\begin{array}{c} {{({\rho ,W)\in \widehat{\mathsf {SU}(n)}}\backslash \Omega (B;n)}} \end{array} }(\dim W){\mathcal {I}}(w,\rho )\ll _{B,w,g}n^{|w|}n^{-2\log B} \end{aligned}$$
  where |*w*| denotes the length of *w* as a reduced word.

The point of (1.10) is not the exact form of the right-hand side, but rather, it gives effective control of the tail. Theorem 1.9 shows that by taking *B* sufficiently large and fixed depending on *w*, the contribution to $\mathbb {E}_{g,n}[\mathrm {tr} _{\gamma }]$ from $(\rho ,W)\in \widehat{\mathsf {SU}(n)}\backslash \Omega (B;n)$ can be made to decay as fast as any $n^{-M}$, for $M\in \mathbf {N}$. We have the following direct corollary of Theorem 1.9, Theorem 1.8, and Corollary 1.6.

### Corollary 1.10

Suppose that $g\ge 2$, $\gamma \in \Gamma _{g}$, and $w\in \mathrm {\mathbf {F}} _{2g}$ represents the conjugacy class of $\gamma$. For any $B\in \mathbf {N}$, we have as $n\rightarrow \infty$

$$\begin{aligned} \mathbb {E}_{g,n}[\mathrm {tr} _{\gamma }]=\zeta (2g-2;n)^{-1}\sum _{\begin{array}{c} {{({\rho ,W)\in }\Omega (B;n)}} \end{array} }(\dim W){\mathcal {I}}(w,\rho )+O_{B,w,g}\left( n^{|w|}n^{-2\log B}\right) . \end{aligned}$$

### Related works I: Spaces of representations

The existence of an asymptotic expansion of $\mathbb {E}_{g,n}[\mathrm {tr} _{\gamma }]$ as in Theorem 1.1 follows a long line of related results. The most closely related of these is the analog of Theorem 1.1 when $\mathsf {SU}(n)$ is replaced by the family of symmetric groups $S_{n}$. For $\pi \in S_{n}$, let $\mathsf {fix}(\rho )$ denote the number of fixed points of $\pi$, and for $\gamma \in \Gamma _{g}$, let $\mathsf {fix}_{\gamma }:\mathrm {Hom}(\Gamma _{g},S_{n})\rightarrow \mathbf {N}$ be the function $\mathsf {fix}_{\gamma }(\phi ){\mathop {=}\limits ^{\mathrm {def}}}\mathsf {fix}(\phi (\gamma ))$. The representation space $\mathrm {Hom}(\Gamma _{g},S_{n})$ is finite and we let $\mathbb {E}_{g,S_{n}}[\mathsf {fix}_{\gamma }]$ denote the expected value of $\mathsf {fix}_{\gamma }$ with respect to the uniform probability measure on $\mathrm {Hom}(\Gamma _{g},S_{n})$. An exactly analogous result to Theorem 1.1 for $\mathbb {E}_{g,S_{n}}[\mathsf {fix}_{\gamma }]$ was established by the author and Puder in [MP20, Thm. 1.1].

Similarly, if instead of using a surface group $\Gamma _{g}$, we consider a free group $\mathrm {\mathbf {F}} _{r}$ with $r\ge 2$, for any compact Lie group *G* the representation space $\mathrm {Hom}(\mathrm {\mathbf {F}} _{r},G)$ can be identified with $G^{r}$ and hence can be given the corresponding probability Haar measure. If *G* is finite, this is simply the uniform probability measure. For any character $\chi$ of *G* and $w\in \mathrm {\mathbf {F}} _{r}$, we obtain a function

$$\begin{aligned} \chi _{w}:\mathrm {Hom}(\mathrm {\mathbf {F}} _{r},G)\rightarrow \mathbf {C},\quad \chi _{w}(\phi ){\mathop {=}\limits ^{\mathrm {def}}}\chi (\phi (w)). \end{aligned}$$

Then we can define $\mathbb {E}_{\mathrm {\mathbf {F}} _{r},G}[\chi _{w}]$ to be the expected value of $\chi _{w}$ with respect to the Haar probability measure. Not only is the analog of Theorem 1.1 true for many natural families of $(G(n),\chi (n))$, but actually, in the case of free groups, $\mathbb {E}_{\mathrm {\mathbf {F}} _{r},G(n)}[\chi (n)_{\gamma }]$ is a *rational* function of *n* for *n* sufficiently large. Indeed, for fixed $w\in \mathrm {\mathbf {F}} _{r}$, $\mathbb {E}_{\mathrm {\mathbf {F}} _{r},G(n)}[\chi (n)_{\gamma }]$ agrees with a rational function of *n* for $n\gg _{w}1$ when

- $G(n)=S_{n}$ and $\chi (n)=\mathsf {fix}$ [Nic94, LP10]
- *G*(*n*) is a family of generalized symmetric groups, e.g., hyperoctahedral groups, and $\chi (n)$ is the trace in a natural defining representation [MP19c]
- $$G(n)=\mathsf {U}(n)$$ and $$\chi (n)=\mathrm {tr} $$ [MP19a]
- $G(n)={\mathsf {O}}(n)$ or ${{\mathsf {S}}}{{\mathsf {p}}}(n)$ and $\chi (n)=\mathrm {tr}$ [MP19b].

### Related works II: 2D Yang–Mills theory

The expected values $\mathbb {E}_{g,n}[\mathrm {tr} _{\gamma }]$ are very closely connected with the expected value of Wilson loops in 2D Yang–Mills theory. We briefly explain these connections and some prior work done by theoretical physicists in the area. It is explained by Sengupta in [Sen03, Appendix A] that if $\Sigma _{g}$ is endowed with a Riemannian metric with associated area $A=A(\Sigma _{g})$ then

1.11 $$\begin{aligned} \sum _{\begin{array}{c} {{(\rho ,W)\in \widehat{\mathsf {SU}(n)}}} \end{array} }e^{-\frac{C(\rho )tA}{2n}}(\dim W)\,\mathcal {I}(w,\rho ) \end{aligned}$$

is, in the context of a quantum $\mathsf {SU}(n)$ Yang–Mills theory on $\Sigma _{g}$, a heuristic definition of the expected value of the Wilson loop measuring the trace of the holonomy around the loop in $\Sigma _{g}$ that $w\in \mathrm {\mathbf {F}} _{2g}$ represents. Here $C(\rho )$ is the Casimir eigenvalue of the representation $(\rho ,W)$ and *t* is a coupling constant. (We have inserted the factor $\frac{1}{n}$ in the above exponent so that it matches with, e.g., [GT93].) It is worth pointing out that the methods of the current paper should also allow one to effectively and rigorously approximate (1.11) although this is not pursued here.

The emphasis in the physics literature is not on the rigorous analytic approximation of integrals such as (1.11), but rather, on the interpretation of (1.11) as a formal power series and then reordering the terms and truncating in a formal way. These manipulations are not intended as having rigorous mathematical consequences. For example, as we understand, none of the ‘$\frac{1}{n}$-expansions’ of (1.11) obtained before in any sense rigorously approximate (1.11); nonetheless, they are significant to physicists. Since the values $\mathbb {E}_{g,n}[\mathrm {tr} _{\gamma }]$ that we focus on here correspond to the $t=0$ case of (1.11) via Corollary 1.6, we briefly survey what is known to physicists for general *t*, with the disclaimer that the author is by no means an expert in the concepts of theoretical physics.

In physics literature, the *chiral* expansion means that the $(\rho ,W)$ are parameterized by $(\rho _{n}^{\uplambda },W_{n}^{\uplambda })$ where $\uplambda$ runs over Young diagrams. For this parameterization to work (cf. §§2.4), one should restrict to Young diagrams with less than *n* rows. This is referred to as the *finite-n* expansion. However, in some cases this restriction is lifted and (1.11) is interpreted as a sum over *all* Young diagrams. This is called the *large-n* expansion. In the chiral expansion, the Young diagrams are ordered by the number of boxes that they contain.

The *partition function *of the quantum Yang–Mills theory corresponds to (1.11) in the case $w=\mathrm {id}$, up to a factor $\frac{1}{n}$, and is given by [GT93, 2.4]

$$\begin{aligned} Z(G,tA,n){\mathop {=}\limits ^{\mathrm {def}}}\sum _{\begin{array}{c} {{(\rho ,W)\in \widehat{\mathsf {SU}(n)}}} \end{array} }e^{-\frac{C(\rho )tA}{2n}}\frac{1}{(\dim W)^{2g-2}}. \end{aligned}$$

A large-*n* chiral expansion of the partition function was obtained by Gross and Taylor in [GT93]. The coefficients of this expansion are interpreted in terms of branched covers of surfaces, and from this, Gross and Taylor deduce their titular statement that ‘Two dimensional QCD is a String Theory.’ A finite-*n* chiral expansion of the partition function in terms of branched covers with some extra data was obtained by Baez and Taylor in [BT94]. In [Ram96], Ramgoolam gives a large-*n* chiral expansion of (1.11) in terms of branched covers of surfaces.

In the language of these papers, the expansion we obtain in Theorem 1.1 is a finite-*n*, fully non-chiral expansion of the expected value of a Wilson loop, when the coupling constant is set to zero. The main point is that this asymptotic expansion is established rigorously through Theorem 1.9.

To conclude this section, we mention that there are some other important rigorous results about Wilson loop expectations in Yang–Mills theories; see [AS12, DN20, DGHK17, Hal18, L17] for rigorous results about Wilson loop expectations in 2D quantum Yang–Mills and [Cha19] for a rigorous computation of Wilson loop expectations in strongly coupled *lattice* Yang–Mills theory in higher dimension.

#### Notation

We write $\mathbf {N}$ for the natural numbers (not including zero), $\mathbf {N}_{0}{\mathop {=}\limits ^{\mathrm {def}}}\mathbf {N}\cup \{0\}$, and $\mathbf {Q}$ denotes the rationals. We write $[n]{\mathop {=}\limits ^{\mathrm {def}}}\{1,\ldots ,n\}$ for $n\in \mathbf {N}$ and $[k,\ell ]{\mathop {=}\limits ^{\mathrm {def}}}\{k,k+1,\ldots ,\ell \}$ for $k,\ell \in \mathbf {N}$, $k\le \ell$. If *A* and *B* are sets $A\backslash B$ is the set of elements of *A* that are not in *B*. We write

$$\begin{aligned} (n)_{\ell }{\mathop {=}\limits ^{\mathrm {def}}}n(n-1)\cdots (n-\ell ). \end{aligned}$$

We let $e(\theta ){\mathop {=}\limits ^{\mathrm {def}}}\exp (2\pi i\theta )$ for $\theta \in \mathbf {R}$. If *G* is a group, and $g_{1},g_{2}\in G$, we let $[g_{1},g_{2}]{\mathop {=}\limits ^{\mathrm {def}}}g_{1}g_{2}g_{1}^{-1}g_{2}^{-1}$. We write [*G*, *G*] for the subgroup of *G* generated by elements of the form $[g_{1},g_{2}]$ for $g_{1},g_{2}\in G$. If *V* is a complex vector space and $q\in \mathbf {N}$, we let

$$\begin{aligned} V^{\otimes q}{\mathop {=}\limits ^{\mathrm {def}}}\underbrace{V\otimes _{\mathbf {C}}\cdots \otimes _{\mathbf {C}}V}_{q}; \end{aligned}$$

in general, if we write a tensor product without explicit subscript it is over $\mathbf {C}$. We write $\mathbf {Q}(t)$ for the ring of rational functions in an indeterminate *t*, i.e., ratios of polynomials.

We use Vinogradov notation as follows. If *f* and *h* are functions of $n\in \mathbf {N}$, we write $f\ll h$ to mean that there are constants $n_{0}\ge 0$ and $C_{0}\ge 0$ such that for $n\ge n_{0}$, $f(n)\le C_{0}h(n)$. We write $f=O(h)$ to mean $f\ll |h|$. We write $f\asymp h$ to mean both $f\ll h$ and $h\ll f$. If in any of these statements the implied constants depend on additional parameters, we add these parameters as subscript to $\ll ,O,$ or $\asymp$. Throughout the paper, we view the genus *g* as fixed and so any implied constant may depend on *g*.

## Background

### Young diagrams and tableaux

#### Young diagrams

A *Young diagram *(YD) is a collection of left-aligned rows of identical square boxes in the plane, where the number of boxes in each row is non-increasing from top to bottom. Hence, we use the English convention for Young diagrams throughout the text. Any YD $\uplambda$ also gives a non-increasing sequence of natural numbers $(\uplambda _{1},\uplambda _{2},\ldots ,\uplambda _{\ell (\uplambda )})$ where $\uplambda _{i}$ is the number of boxes in the *i*^th^ (from top to bottom) row of $\uplambda$, and $\ell (\uplambda )$ is the number of rows of $\uplambda$. A finite non-increasing sequence of natural numbers is called a *partition*. We think of partitions and YDs interchangeably in this paper via the above correspondence. For example, the partition (*k*) corresponds to the Young diagram with one row consisting of *k* boxes. The empty YD with no boxes is denoted by $\emptyset$. The *size *of a YD $\uplambda$ is the number of boxes that it contains, or $\sum _{i=1}^{\ell (\uplambda )}\uplambda _{i}$. The size of $\uplambda$ is denoted by $|\uplambda |$, and the statement $|\uplambda |=k$ is sometimes written $\uplambda \vdash k$.

Given two Young diagrams $\uplambda$ and $\mu$, we say $\mu \subset \uplambda$ if every box of $\mu$ is a box of $\uplambda$. A *skew Young diagram *(SYD) is a pair $\uplambda ,\mu$ of Young diagram such that $\mu \subset \uplambda$. This is usually written as $\uplambda /\mu$ and $\uplambda /\mu$ is thought of as the collection of boxes of $\uplambda$ that are not boxes of $\mu$. A Young diagram $\uplambda$ is identified with $\uplambda /\emptyset$, and in this way, YDs are special cases of SYDs.

We will have use for the following relations between Young diagrams. We say $\mu \subset _{k}\uplambda$ if $\mu \subset \uplambda$ and $\uplambda /\mu$ contains *k* boxes. We say $\mu \subset ^{1}\uplambda$ if $\mu \subset \uplambda$ and no two boxes of $\uplambda /\mu$ are in the same column. We say $\mu \subset ^{r}\uplambda$ if there is a sequence $\mu _{1},\ldots ,\mu _{r-1}$ of YDs such that

$$\begin{aligned} \mu \subset ^{1}\mu _{1}\subset ^{1}\cdots \subset ^{1}\mu _{r-1}\subset ^{1}\uplambda . \end{aligned}$$

Finally, we write $\mu \subset _{k}^{r}\uplambda$ if both $\mu \subset _{k}\uplambda$ and $\mu \subset ^{r}\uplambda$.

#### Semistandard Young tableaux

Given a SYD $\uplambda /\mu$ (which may in fact be a YD) and a subset $S\subset \mathbf {N}$, a *semistandard tableau* of shape $\uplambda /\mu$ with entries in *S* is a filling of the boxes of $\uplambda /\mu$ with the numbers of *S* such that the numbers in the boxes are strictly increasing along columns from top to bottom and non-strictly increasing along rows from left to right. If $\uplambda /\mu$ is a SYD, we write

$$\begin{aligned} \mathcal {SST}_{[k,\ell ]}(\uplambda /\mu ) \end{aligned}$$

for the semistandard tableaux of shape $\uplambda /\mu$ with entries in $[k,\ell ]$. We also use all obvious variants of this notation, e.g., for YD $\uplambda$ and $n\in \mathbf {N}$, $\mathcal {SST}_{[n]}(\uplambda )$ is the collection of semistandard tableaux of shape $\uplambda$ with entries in [*n*]. There is by convention a unique semistandard tableau (with any entry set) of shape the empty YD (or an empty SYD).

If $\uplambda /\mu$ is a SYD, $n>m$, $T_{1}\in \mathcal {SST}_{[m]}(\mu )$ and $T_{2}\in \mathcal {SST}_{[m+1,n]}(\uplambda /\mu )$, we write $T_{1}\sqcup T_{2}$ for the semistandard tableau obtained by adjoining the numbers-in-boxes of $T_{2}$ to those of $T_{1}$. It is easy to see this is always a valid semistandard tableau of shape $\uplambda$.

Given $T\in \mathcal {SST}_{[k,m]}(\uplambda /\mu )$, the *weight *of *T* is the function

$$\begin{aligned} \omega _{T}:[k,m]\rightarrow \mathbf {N}_{0}, \end{aligned}$$

where $\omega _{T}(j)$ is the number of occurrences of *j* in *T*.

Given $T\in \mathcal {SST}_{[n]}(\uplambda )$, $\uplambda$ a YD, and $m\in \mathbf {N}_{0}$ with $m\le n$, we write $T|_{>m}$ for the skew semistandard tableau formed by the numbers-in-boxes of *T* with numbers $>m$, and similarly write $T|_{\le m}$ for the semistandard tableau formed by the numbers-in-boxes of *T* with numbers $\le m$.

### General representation theory

Here we clarify the language of representation theory that will appear throughout the paper.

A unitary representation of a compact Lie group^3^*G* consists of a Hilbert space^4^$\mathcal {H}$ and a homomorphism $\rho _{1},G\rightarrow \mathsf {U}(\mathcal {H})$ where $\mathsf {U}(\mathcal {H})$ is the group of unitary operators on $\mathcal {H}$. Any compact Lie group has a trivial unitary representation $(\mathrm {triv}_{G},\mathbf {C})$ with inner product on $\mathbf {C}$ given by $\langle z_{1},z_{2}\rangle =z_{1}\overline{z_{2}}$ and $\mathrm {triv}_{G}(g)=1$ for all $g\in G$.

If $(\rho _{1},\mathcal {H}_{1})$ and $(\rho _{2},\mathcal {H}_{2})$ are two finite-dimensional representations of a compact Lie group *G*, an *intertwiner* between $(\rho _{1},\mathcal {H}_{1})$ and $(\rho _{2},\mathcal {H}_{2})$ is a linear map $I:\mathcal {H}_{1}\rightarrow \mathcal {H}_{2}$ such that for all $g\in G$, $I\rho _{1}(g)=\rho _{2}(g)I$. If there is an invertible intertwiner between $(\rho _{1},\mathcal {H}_{1})$ and $(\rho _{2},\mathcal {H}_{2})$, we say $(\rho _{1},\mathcal {H}_{1})$ and $(\rho _{2},\mathcal {H}_{2})$ are *linearly isomorphic. *If there is an invertible isometric intertwiner between the two, we say they are unitarily equivalent or isomorphic as unitary representations.

In many cases, the maps $\rho _{1}$ and $\rho _{2}$ will be tacitly inferred from the group *G* and the spaces $\mathcal {H}_{1}$ and $\mathcal {H}_{2}$. When this is the case, we write

$$\begin{aligned} \mathrm {Hom}_{G}(\mathcal {H}_{1},\mathcal {H}_{2}) \end{aligned}$$

for the vector space of linear intertwiners between $(\rho _{1},\mathcal {H}_{1})$ and $(\rho _{2},\mathcal {H}_{2})$.

For $(\rho ,W)$ a unitary representation of a compact Lie group *G*, and $H\le G$ a compact Lie subgroup with unitary representation $(\pi ,V)$, we define the $(\pi ,V)$*-isotypic subspace of* *W* *for the subgroup* *H* to be the subspace spanned by the images of all elements of

$$\begin{aligned} \mathrm {Hom}_{H}(V,W){\mathop {=}\limits ^{\mathrm {def}}}\mathrm {Hom}_{H}(V,\mathrm {Res}_{H}^{G}W) \end{aligned}$$

where $\mathrm {Res}_{H}^{G}W$ is the *restriction* of $\rho$ to *H*. Any such isotypic space is itself a unitary subrepresentation of $\mathrm {Res}_{H}^{G}W$ (for the group *H*). If *V* is an irreducible representation of *H*, the dimension of $\mathrm {Hom}_{H}(V,W)$ is the *multiplicity *with which *V* appears in $\mathrm {Res}_{H}^{G}W$.

Let $(\pi ,W)$, $(\pi _{1},W_{1})$ and $(\pi _{2},W_{2})$ be finite-dimensional unitary representations of a compact Lie group. We explain some basic constructions.

There is a dual unitary representation on the space of complex linear functionals on *W* with inner product induced by that on *W*; this representation is also irreducible if $(\pi ,W)$ is. The action of *g* on a linear functional $\varphi$ on *W* is by $g[\varphi ](w)=\varphi (g^{-1}w)$. If $w\in W$, we write ${\check{w}}\in {\check{W}}$ for the linear functional

$$\begin{aligned} {\check{w}}:v\mapsto \langle v,w\rangle . \end{aligned}$$

We denote the dual representation by $({\check{\pi }},{\check{W}})$ or simply ${\check{W}}$. Given two finite-dimensional unitary representations $(\pi _{1},W_{1})$ and $(\pi _{2},W_{2})$ of a compact Lie group *G*, the tensor product $W_{1}\otimes W_{2}{\mathop {=}\limits ^{\mathrm {def}}}W_{1}\otimes _{\mathbf {C}}W_{2}$ has an action of *G* that is ‘diagonal’ by $\pi _{1}$ on the first factor and $\pi _{2}$ on the second factor. The tensor product inherits a Hermitian inner product from that on $W_{1}$ and $W_{2}$ where $\langle w_{1}\otimes w_{2},w'_{1}\otimes w'_{2}\rangle {\mathop {=}\limits ^{\mathrm {def}}}\langle w_{1},w'_{1}\rangle \langle w_{2},w'_{2}\rangle$ that makes $W_{1}\otimes W_{2}$ a unitary representation of *G* under the diagonal action. This extends to tensor powers of *W*; any $W^{\otimes k}$ for $k\in \mathbf {N}$ is in this way a unitary representation of *G* under the diagonal action.

There is a canonical isomorphism

2.1 $$\begin{aligned} W_{1}\otimes \check{W_{2}}\cong \mathrm {Hom}(W_{2},W_{1}) \end{aligned}$$

of linear representations, where the right-hand side is the vector space of linear maps from $W_{2}$ to $W_{1}$, where *G* acts ‘diagonally’ on the left-hand side, and by conjugation ($g:A\mapsto \pi _{1}(g)A\pi _{2}(g)^{-1}$) on the right-hand side. If $W_{1}',W_{2}'$ are subrepresentations of $W_{1},W_{2}$, then we view $\mathrm {Hom}(W'_{2},W'_{1})$ as a subrepresentation of $\mathrm {Hom}(W_{2},W_{1})$ via (2.1). This corresponds to extending linear maps by 0 on the orthogonal complement of $W'_{2}$ in $W_{2}$.

In the case $W_{1}=W_{2}=W$ (2.1) is moreover an isomorphism of unitary representations $W\otimes {\check{W}}\cong \mathrm {End}(W)$ if $\mathrm {End}(W)$ is given the Hilbert–Schmidt inner product $\langle A,B\rangle {\mathop {=}\limits ^{\mathrm {def}}}\mathrm {tr} (AB^{*})$, $B^{*}$ standing for the Hermitian transpose of *B*.

### Representation theory of symmetric groups

Although the problems of this paper are not initially posed in a way that involves symmetric groups, the representation theory of unitary groups is intimately connected via Schur–Weyl duality (see $\hbox { \char "0A7}$2.4) to the representation theory of symmetric groups, so it plays a large part in this paper.

We write $S_{k}$ for the symmetric group of permutations of the set [*k*], and $\mathbf {C}[S_{k}]$ for the group algebra of $S_{k}$. As a technicality, the group $S_{0}$ is the group with one element.

The equivalence classes of irreducible representations of $S_{k}$ are in one-to-one correspondence with YDs $\uplambda \vdash k$ [FH91, §4.2]. The irreducible unitary representation of $S_{k}$ corresponding to $\uplambda \vdash k$ will be denoted by $(\pi _{\uplambda },V^{\uplambda })$ and simply referred to as $V^{\uplambda }$. We write $\chi _{\uplambda }$ for the character of $V^{\uplambda }$, i.e.,

$$\begin{aligned} \chi _{\uplambda }(\sigma ){\mathop {=}\limits ^{\mathrm {def}}}\mathrm {tr} (\pi _{\uplambda }(\sigma )),\quad \sigma \in S_{k}. \end{aligned}$$

All characters of irreducible representations of symmetric groups are real-valued (e.g., by [FH91, Frobenius Formula 4.10]).

The dimension of $V^{\uplambda }$ is given by the Frame–Robinson–Thrall hook length formula as follows. The *hook *of a box $\square$ in $\uplambda \vdash k$ is the collection of boxes either to the right of, or below, $\uplambda$, including the box itself. We write $h_{\uplambda }(\square )$ for the number of boxes in the hook of $\square$. The hook length formula [FRT54] states

2.2 $$\begin{aligned} d_{\uplambda }{\mathop {=}\limits ^{\mathrm {def}}}\dim V^{\uplambda }=\frac{k!}{\prod _{\square \in \uplambda }h_{\uplambda }(\square )}. \end{aligned}$$

Before proceeding, we fix some notation. If we refer to $S_{\ell }\le S_{k}$ with $\ell \le k$ we always view $S_{\ell }$ as the subgroup of permutations that fix every element of $[\ell +1,k]$. As a consequence, we obtain fixed inclusions $\mathbf {C}[S_{\ell }]\subset \mathbf {C}[S_{k}]$ for $\ell$ and *k* as above. When we write $S_{\ell }\times S_{k-\ell }\le S_{k}$, the first factor is $S_{\ell }$ as defined above and the second factor is $S'_{k-\ell }$ which is our notation for the subgroup of permutations that fix every element of $[\ell ]$.

Given $\uplambda \vdash \ell$, the element

$$\begin{aligned} \mathfrak {p}_{\uplambda }{\mathop {=}\limits ^{\mathrm {def}}}\frac{d_{\uplambda }}{\ell !}\sum _{\sigma \in S_{\ell }}\chi _{\uplambda }(\sigma )\sigma \in \mathbf {C}[S_{\ell }] \end{aligned}$$

is a central idempotent in $\mathbf {C}[S_{\ell }]$ with the following important property. If $(\pi ,V)$ is any unitary irreducible representation of $S_{k}$ with $k\ge \ell$, then by linear extension $\pi :\mathbf {C}[S_{k}]\rightarrow \mathrm {End}(V)$. Under the fixed inclusion $\mathbf {C}[S_{\ell }]\subset \mathbf {C}[S_{k}]$, $\pi (\mathfrak {p}_{\uplambda })$ is the orthogonal projection onto the $V^{\uplambda }$-isotypic subspace of *V* for the subgroup $S_{k}$.

Suppose that $\ell \le k$, $\ell ,k\in \mathbf {N}_{0}$, with $\uplambda \vdash k$ and $\mu \vdash \ell$. We write

$$\begin{aligned} d_{\uplambda /\mu }{\mathop {=}\limits ^{\mathrm {def}}}\dim \mathrm {Hom}_{S_{\ell }}(V^{\mu },V^{\uplambda }). \end{aligned}$$

The branching rules for $S_{k}$ imply that $d_{\uplambda /\mu }=0$ unless $\mu \subset \uplambda$. Applying Frobenius reciprocity to the pair $(V^{\mu },V^{\uplambda })$ then taking the dimension of the induced representation $\mathrm {Ind}_{S_{\ell }}^{S_{k}}V^{\mu }$ gives the formula, for $\mu \vdash \ell$ fixed and $b=k-\ell$,

2.3 $$\begin{aligned} \sum _{\mu \subset _{b}\uplambda }d_{\uplambda /\mu }d_{\uplambda }=\frac{(\ell +b)!}{\ell !}d_{\mu }. \end{aligned}$$

Suppose that $\ell _{1},\ell _{2}\in \mathbf {N}_{0}$ and $\ell _{1}+\ell _{2}=k$. The irreducible representations of $S_{\ell _{1}}\times S_{\ell _{2}}$ are of the form $V^{\mu _{1}}\otimes V^{\mu _{2}}$ with $\mu _{i}\vdash \ell _{i}$ for $i=1,2$; $S_{\ell _{1}}$ acts on the first factor and $S'_{\ell _{2}}$ acts on the second factor. The numbers

$$\begin{aligned} \mathsf {LR}_{\mu _{1},\mu _{2}}^{\uplambda }{\mathop {=}\limits ^{\mathrm {def}}}\dim \mathrm {Hom}_{S_{\ell _{1}}\times S_{\ell _{2}}}(V^{\mu _{1}}\otimes V^{\mu _{2}},V^{\uplambda })\in \mathbf {N}_{0} \end{aligned}$$

are called *Littlewood–Richardson coefficients.* They are notoriously difficult to work with, but thankfully, in this paper the most detail we need about them is the following:

#### Lemma 2.1

(Recast of Pieri’s formula). Suppose that $\ell _{1},\ell _{2}\in \mathbf {N}_{0}$ and $\ell _{1}+\ell _{2}=k$. Let $\mu \vdash \ell _{1}$ and $\uplambda \vdash k$ with $\mu \subset ^{1}\uplambda$. We have

$$\begin{aligned} \mathsf {LR}_{\mu ,(\ell _{2})}^{\uplambda }=\dim \mathrm {Hom}_{S_{\ell _{1}}\times S_{\ell _{2}}}(V^{\mu }\otimes \mathrm {triv}_{S_{\ell _{2}}},V^{\uplambda })=1. \end{aligned}$$

#### Proof

This is a standard fact but normally presented slightly differently. To obtain this version, one needs to know that $\mathsf {LR}_{\mu ,(\ell _{2})}^{\uplambda }$ is also the coefficient of the Schur polynomial $s_{\mu }$ in the expansion of $s_{\uplambda }s_{(\ell _{2})}$ in Schur polynomials (see [FH91, eq. (A.8), Ex. 4.43] for the equivalence of these definitions of Littlewood–Richardson coefficients). Then the lemma follows from the version of Pieri’s formula for $s_{\uplambda }s_{(\ell _{2})}$ given in [FH91, eq. (A.7)]. $\quad \square$

### Representation theory of $\mathsf {U}(n)$ and $\mathsf {SU}(n)$

Here we give a brief account of the representation theory of $\mathsf {U}(n)$ and $\mathsf {SU}(n)$. The details as well as more background can be found in [FH91].

The equivalence classes of irreducible representations of $\mathsf {U}(n)$ are in one-to-one correspondence with their highest weights by the theorem of the highest weight. These highest weights are given by linear combinations

$$\begin{aligned} \uplambda _{1}{\hat{\omega }}_{1}+\cdots +\uplambda _{n}{\hat{\omega }}_{n} \end{aligned}$$

where $(\uplambda _{1},\ldots ,\uplambda _{n})\in \mathbf {Z}^{n}$ is a non-increasing sequence of integers and ${\hat{\omega }}_{1},\ldots ,{\hat{\omega }}_{n}$ is a system of fundamental weights for $\mathsf {U}(n)$. The sequence $(\uplambda _{1},\ldots ,\uplambda _{n})$ is called the *signature* of the representation. If all $\uplambda _{i}\in \mathbf {Z}_{\ge 0}$, then the signature corresponds to a Young diagram $\uplambda$ with $\ell (\uplambda )\le n$, and conversely, any YD $\uplambda$ with $\ell (\uplambda )\le n$ gives rise to an equivalence class of irreducible representation of $\mathsf {U}(n)$ denoted by $({\tilde{\rho }}_{n}^{\uplambda },W_{n}^{\uplambda })$.

Every irreducible representation of $\mathsf {U}(n)$ restricts to an irreducible representation of $\mathsf {SU}(n)$, and every irreducible representation of $\mathsf {SU}(n)$ arises by restriction from $\mathsf {U}(n)$. Two irreducible representations of $\mathsf {U}(n)$ restrict to equivalent representations of $\mathsf {SU}(n)$ if and only if their signatures differ by an integer multiple of $(1,1,\ldots ,1)$. This immediately shows that the equivalence classes of irreducible representations of $\mathsf {SU}(n)$ are in one-to-one correspondence with YDs $\uplambda$ such that $\ell (\uplambda )\le n-1$, since there is a unique signature $(\uplambda _{1},\ldots ,\uplambda _{n})$ whose representation restricts to the given one of $\mathsf {SU}(n)$ with $\uplambda _{n}=0$ and this corresponds to a YD by deleting trailing zeros. For such a $\uplambda ,$ we write $(\rho _{n}^{\uplambda },W_{n}^{\uplambda })$ for the restriction of this representation of $\mathsf {SU}(n)$.

*To be less verbose, in the rest of the paper we will tend to refer to representations simply by the vector space whenever the module structure can be inferred.*

The trace of $g\in \mathsf {U}(n)$ on $W_{n}^{\uplambda }$ is given by $s_{\uplambda }(e(\theta _{1}),\ldots ,e(\theta _{n}))$ where $s_{\uplambda }$ is the *Schur polynomial* associated with the YD $\uplambda$ and $e(\theta _{1}),\ldots ,e(\theta _{n})$ are the eigenvalues of *g*. As such, the dimension of $W_{n}^{\uplambda }$ is given by the specialization of the Schur polynomial, letting $1^{n}{\mathop {=}\limits ^{\mathrm {def}}}(\underbrace{1,1,\ldots ,1}_{n})$

2.4 $$\begin{aligned} D_{\uplambda }(n){\mathop {=}\limits ^{\mathrm {def}}}\dim W_{n}^{\uplambda }=s_{\uplambda }(1^{n}). \end{aligned}$$

There is a formula for $s_{\uplambda }(1^{n})$ due to Stanley called the *hook content *formula. Given a YD $\uplambda$, for any box $\square$ of $\uplambda$, we define the *content* of the box to be

$$\begin{aligned} c(\square ){\mathop {=}\limits ^{\mathrm {def}}}j(\square )-i(\square ) \end{aligned}$$

where $i(\square )$ is the row number (starting at 1, counting from top to bottom) of the box and $j(\square )$ is the column number (starting at 1, counting from left to right). Recall the quantities $h_{\uplambda }(\square )$ from §§2.3. The hook content formula [Sta99, Cor. 7.21.4] says

2.5 $$\begin{aligned} D_{\uplambda }(n)=s_{\uplambda }(1^{n})=\frac{\prod _{\square \in \uplambda }(n+c(\square ))}{\prod _{\square \in \uplambda }h_{\uplambda }(\square )}. \end{aligned}$$

By a slight abuse of notation, if *g* is any element of $\mathsf {U}(n)$, we also write $s_{\uplambda }(g)$ for $\mathrm {tr} _{W_{n}^{\uplambda }}({\tilde{\rho }}_{\uplambda }(g))$; strictly speaking this is the Schur polynomial evaluated at the eigenvalues of *g*. The hook content formula (2.5) implies that for a fixed YD $\uplambda$, $D_{\uplambda }(n)$ is a polynomial function of *n* with coefficients in $\mathbf {Q}$.

There is an important formula expressing $s_{\uplambda }(g)$ in terms of power sum symmetric functions. Given a partition $\uplambda =(\uplambda _{1},\ldots ,\uplambda _{r})$, and $g\in \mathsf {U}(n)$, we define

$$\begin{aligned} p_{\uplambda }(g){\mathop {=}\limits ^{\mathrm {def}}}\mathrm {tr} (g^{\uplambda _{1}})\mathrm {tr} (g^{\uplambda _{2}})\cdots \mathrm {tr} (g^{\uplambda _{r}}). \end{aligned}$$

Given $\uplambda \vdash k$, the change of base formula is [FH91, Ex. A.29]

2.6 $$\begin{aligned} s_{\uplambda }(g)=\frac{1}{k!}\sum _{\pi \in S_{k}}\chi _{\uplambda }(\pi )p_{\mu (\pi )}(g) \end{aligned}$$

where $\mu (\pi )\vdash k$ is the partition given by the cycle type of $\pi \in S_{k}$.

Besides the *polynomial *families of representations of $\mathsf {U}(n)$, as *n* varies, obtained by fixing $\uplambda$ and varying *n*, there are similar *rational *families that we explain now*. *Let $\mu \vdash k$ and $\nu \vdash \ell$ be fixed Young diagrams. Then for every $n\ge \ell (\mu )+\ell (\nu )$, there is an irreducible unitary representation of $\mathsf {U}(n)$ with signature

$$\begin{aligned} (\mu _{1},\mu _{2},\ldots ,\mu _{\ell (\mu )},\underbrace{0,\ldots ,0}_{n-\ell (\mu )-\ell (\nu )},-\nu _{\ell (\nu )},-\nu _{\ell (\nu )-1},\ldots ,-\nu _{1}). \end{aligned}$$

We write $W_{n}^{[\mu ,\nu ]}$ for this representation of $\mathsf {U}(n)$, $D_{[\mu ,\nu ]}(n)$ for its dimension, and $s_{[\mu ,\nu ]}(g)$ for the character of this representation at $g\in \mathsf {U}(n)$. The representations $W_{n}^{[\mu ,\nu ]}$ directly generalize the case of $W_{n}^{\uplambda }$ with $\uplambda$ fixed by taking $\mu =\uplambda$ and $\nu$ to be the empty Young diagram. The character $s_{[\uplambda ,\mu ]}(g)$ can be written in terms of Schur polynomials by a result of Koike.

#### Theorem 2.2

([Koi89, eq.(0.2)]). For $\mu \vdash k$ and $\nu \vdash \ell$, $n\ge \ell (\mu )+\ell (\nu )$, $g\in \mathsf {U}(n)$, we have

$$\begin{aligned} s_{[\mu ,\nu ]}(g)=\sum _{\begin{array}{c} p_{1},p_{2},p_{3}\in \mathbf {N}_{0}\\ p_{1}+p_{2}=k,\quad p_{1}+p_{3}=\ell \end{array} }\sum _{\begin{array}{c} \nu _{1}\vdash p_{1},\nu _{2}\vdash p_{2},\nu _{3}\vdash p_{3}\\ \nu _{2}\subset \mu ,\nu _{3}\subset \nu \end{array} }\mathsf {LR}_{\nu _{1},\nu _{2}}^{\mu }\mathsf {LR}_{\check{\nu _{1}},\nu _{3}}^{\nu }s_{\nu _{2}}(g)s_{\nu _{3}}(g^{-1}) \end{aligned}$$

where $\check{\nu _{1}}$ denotes the transposed YD of $\nu _{1}$ (obtained from $\nu _{1}$ by switching rows and columns).

Inspection of Theorem 2.2 shows that $D_{[\mu ,\nu ]}(n)=s_{[\mu ,\nu ]}(1^{n})$ is a finite linear combination, with integer coefficients, of

2.7 $$\begin{aligned} s_{\nu _{2}}(1^{n})s_{\nu _{3}}(1^{n}),\quad \nu _{2}\vdash p_{2}\le k,\nu _{3}\vdash p_{3}\le \ell . \end{aligned}$$

The linear combination itself does not depend on *n*, and by the hook content formula (2.5), each of the terms in (2.7) is a polynomial function of *n* with coefficients in $\mathbf {Q}$ for $n\ge \ell (\mu )+\ell (\nu )$. Moreover, the term in (2.7) is $\asymp n^{p_{2}+p_{3}}$ so the contribution to $s_{[\uplambda ,\mu ]}(1^{n})$ of maximal growth corresponds to $p_{2}=k$ and $p_{3}=\ell$ hence is a unique term with nonzero (in fact, unity) coefficient. These arguments yield the following corollary to Theorem 2.2.

#### Corollary 2.3

Let $\mu \vdash k$ and $\nu \vdash \ell$. For $n\ge \ell (\mu )+\ell (\nu )$, $D_{[\mu ,\nu ]}(n)$ is given by a polynomial function of *n* with coefficients in $\mathbf {Q}$ and

$$\begin{aligned} D_{[\mu ,\nu ]}(n)\asymp n^{k+\ell } \end{aligned}$$

as $$n\rightarrow \infty $$.

Corollary 2.3 shows that in any organization of the irreducible representations of $\mathsf {U}(n)$ or $\mathsf {SU}(n)$ into families of ‘small’- or ‘large’-dimensional representations, the representations $W_{n}^{[\mu ,\nu ]}$ with $\mu ,\nu$ fixed should be considered alongside the representations $W_{n}^{\uplambda }$ with $\uplambda$ fixed.

#### Schur–Weyl duality

We always understand that $\mathbf {C}^{n}$ has the standard Hermitian inner product and $\mathsf {U}(n)$ acts unitarily on $\mathbf {C}^{n}$ in its defining representation. This representation coincides with $({\tilde{\rho }}_{\uplambda },W_{n}^{\uplambda })$ where $\uplambda$ consists of one box in the formalism of $\hbox { \char "0A7}$2.4. We write $\{e_{1},\ldots ,e_{n}\}$ for the standard basis of $\mathbf {C}^{n}$. The tensor power $(\mathbf {C}^{n})^{\otimes k}{\mathop {=}\limits ^{\mathrm {def}}}\underbrace{\mathbf {C}^{n}\otimes \cdots \otimes \mathbf {C}^{n}}_{k}$ has an induced inner product that makes it into a unitary representation of $\mathsf {U}(n)$, where $\mathsf {U}(n)$ acts diagonally. The space $(\mathbf {C}^{n})^{\otimes k}$ is also a unitary representation of $S_{k}$ where $S_{k}$ acts by permuting indices. The actions of $\mathsf {U}(n)$ and $S_{k}$ commute, and hence, $(\mathbf {C}^{n})^{\otimes k}$ is a unitary representation of $\mathsf {U}(n)\times S_{k}$. We write $\pi _{n}^{k}:\mathsf {U}(n)\rightarrow \mathrm {End}((\mathbf {C}^{n})^{\otimes k})$ for the diagonal action of $\mathsf {U}(n)$ and $\rho _{n}^{k}:S_{k}\rightarrow \mathrm {End}((\mathbf {C}^{n})^{\otimes k})$ for the action that permutes coordinates. Schur–Weyl duality gives the following full description of the decomposition of $(\mathbf {C}^{n})^{\otimes k}$ into irreducible representations of $\mathsf {U}(n)\times S_{k}$, which are a priori tensor products $W\otimes V$ where *W* (resp. *V*) is an irreducible representation of $\mathsf {U}(n)$ (resp. $S_{k}$).

##### Proposition 2.4

(Schur–Weyl duality [Wey39, Chapt. IV]). There is an isomorphism $\mathcal {F}$ of linear representations of $\mathsf {U}(n)\times S_{k}$

$$\begin{aligned} \mathcal {F}:(\mathbf {C}^{n})^{\otimes k}\xrightarrow {\sim }\bigoplus _{\begin{array}{c} \uplambda \vdash k\\ \ell (\uplambda )\le n \end{array} }W_{n}^{\uplambda }\otimes V^{\uplambda }. \end{aligned}$$

#### Branching rules

For any $n>r\ge 0$ we view $\mathsf {U}(n-r)$ as the subgroup of $\mathsf {U}(n)$ of elements that fix pointwise the standard basis elements $e_{n-r+1},\ldots ,e_{n}$ of $\mathbf {C}^{n}$ in the defining representation of $\mathsf {U}(n)$. We have [FH91, Ex. 6.12] for $\ell (\uplambda )\le n$

2.8 $$\begin{aligned} \mathrm {Res}_{\mathsf {U}(n-1)}^{\mathsf {U}(n)}W_{n}^{\uplambda }\cong \bigoplus _{\begin{array}{c} \mu \,:\,\mu \subset ^{1}\uplambda \\ \ell (\mu )\le n-1 \end{array} }W_{n-1}^{\mu }. \end{aligned}$$

By iterating (2.8), we obtain a orthogonal direct sum decomposition

$$\begin{aligned} W_{n}^{\uplambda }&=\bigoplus _{\begin{array}{c} \emptyset \subset ^{1}\mu _{1}\subset ^{1}\ldots \subset ^{1}\mu _{n-1}\subset ^{1}\uplambda \\ \ell (\mu _{i})\le i \end{array} }W_{n,(\mu _{1},\ldots ,\mu _{n-1})}^{\uplambda } \end{aligned}$$

where

$$\begin{aligned} W_{n,(\mu _{1},\ldots ,\mu _{n-1})}^{\uplambda }{\mathop {=}\limits ^{\mathrm {def}}}\bigcap _{i=1}^{n-1}(W_{n}^{\uplambda })_{\mu _{i}} \end{aligned}$$

is one-dimensional and $(W_{n}^{\uplambda })_{\mu _{i}}$ denotes the $W_{i}^{\mu _{i}}$-isotypic subspace of $W_{n}^{\uplambda }$ for $\mathsf {U}(i)$. We make the observation that a sequence

2.9 $$\begin{aligned} \emptyset {\mathop {=}\limits ^{\mathrm {def}}}\mu _{0}\subset ^{1}\mu _{1}\subset ^{1}\ldots \subset ^{1}\mu _{n-1}\subset ^{1}\mu _{n}{\mathop {=}\limits ^{\mathrm {def}}}\uplambda \end{aligned}$$

gives a semistandard tableau of shape $\uplambda$ by filling in the boxes of $\mu _{i+1}/\mu _{i}$ with the number *i*. This gives a one-to-one correspondence $(\mu _{_{1}},\ldots ,\mu _{n-1})\mapsto T(\mu _{1},\ldots ,\mu _{n-1})$ between such sequences of YDs as in (2.9) and $\mathcal {SST}_{[n]}(\uplambda )$; for $T=T(\mu _{1},\ldots ,\mu _{n-1})\in \mathcal {SST}_{[n]}(\uplambda )$ we define

$$\begin{aligned} W_{n,T}^{\uplambda }{\mathop {=}\limits ^{\mathrm {def}}}W_{n,(\mu _{1},\ldots ,\mu _{n-1})}^{\uplambda } \end{aligned}$$

and pick a unit-norm vector $w_{T}$ in each $W_{n,T}^{\uplambda }$ (this is unique up to a unit complex number). We do this now once and for all for all $\uplambda$ and $T\in \mathcal {SST}_{[n]}(\uplambda )$. The resulting orthonormal basis

$$\begin{aligned} \{\,w_{T}\,:\,T\in \mathcal {SST}_{[n]}(\uplambda )\,\} \end{aligned}$$

of $W_{n}^{\uplambda }$ is called a *Gelfand–Tsetlin basis. *

Given $m,n\in \mathbf {N}_{0}$ with $m\le n$, YDs $\uplambda ,\mu$ with $\mu \subset ^{n-m}\uplambda$, $\ell (\mu )\le m$, $\ell (\uplambda )\le n$, and $R_{1},R_{2}\in \mathcal {SST}_{[m+1,n]}(\uplambda /\mu )$ we define

2.10 $$\begin{aligned} \mathcal {E}_{R_{1},R_{2}}^{\uplambda ,\mu ,m}{\mathop {=}\limits ^{\mathrm {def}}}\frac{1}{\sqrt{D_{\mu }(m)}}\sum _{T\in \mathcal {SST}_{[m]}(\mu )}w_{T\sqcup R_{1}}\otimes {\check{w}}_{T\sqcup R_{2}}\in \mathrm {End}(W_{n}^{\uplambda }). \end{aligned}$$

We have use for the following fact, analogous to [MP20, Lemma 2.4].

##### Lemma 2.5

Let $\uplambda$ be a Young diagram with $\ell (\uplambda )\le n$. Suppose $m\in [n]$. Let $Z(\uplambda ,m)\subset \mathrm {End}(W_{n}^{\uplambda })$ denote the algebra generated by $\mathsf {U}(m)$ acting on $W_{n}^{\uplambda }$. The commutant of $Z(\uplambda ,m)$ in $\mathrm {End}(W_{n}^{\uplambda })$ has an orthonormal basis given by

$$\begin{aligned} \left\{ \mathcal {E}_{R_{1},R_{2}}^{\uplambda ,\mu ,m}\,:\,\mu \subset ^{n-m}\uplambda ,\,\ell (\mu )\le m,\,R_{1},R_{2}\in \mathcal {SST}_{[m+1,n]}(\uplambda /\mu )\right\} . \end{aligned}$$

##### Proof

The proof is essentially the same as that of [MP20, Lemma 2.4]. $\quad \square$

### The Weingarten calculus

The *Weingarten calculus* is a method of calculating integrals of the form

2.11 $$\begin{aligned} \int _{u\in \mathsf {U}(n)}u_{i_{1}j_{1}}\cdots u_{i_{k}j_{k}}{\bar{u}}_{i'_{1}j'_{1}}\cdots {\bar{u}}_{i'_{k}j'_{k}}d\mu _{\mathsf {U}(n)}^{\mathrm {Haar}}(u) \end{aligned}$$

where $d\mu _{\mathsf {U}(n)}^{\mathrm {Haar}}$ is the probability Haar measure on $\mathsf {U}(n)$. A large-*n* asymptotic estimate for (2.11) was first obtained by Weingarten in [Wei78] and this was expanded upon by Xu in [Xu97] where a full large-*n* asymptotic expansion was given. A new method of evaluating (2.11) in terms of characters of symmetric groups was developed by Collins [Col03] and Collins and Śniady [CŚ06] and this is what we refer to here as the Weingarten calculus. The work [CŚ06] involves reinterpreting (2.11) as a problem in calculating the orthogonal projection onto the $\mathsf {U}(n)$-invariant vectors in $\mathrm {End}((\mathbf {C}^{n})^{\otimes k})$, where $u\in \mathsf {U}(n)$ acts on $A\in \mathrm {End}((\mathbf {C}^{n})^{\otimes k})$ by $A\mapsto \pi _{n}^{k}(u)A\pi _{n}^{k}(u^{-1})$, $\pi _{n}^{k}:\mathsf {U}(n)\rightarrow \mathrm {End}((\mathbf {C}^{n})^{\otimes k})$ the diagonal action. This is actually the point of view that will be relevant to this paper.

The *Weingarten function, *depending on parameters *n*, *k* is the following element of $\mathbf {C}[S_{k}]$

2.12 $$\begin{aligned} \mathrm {Wg}_{n,k}{\mathop {=}\limits ^{\mathrm {def}}}\frac{1}{(k!)^{2}}\sum _{\begin{array}{c} \uplambda \vdash k\\ \ell (\uplambda )\le n \end{array} }\frac{d_{\uplambda }^{2}}{D_{\uplambda }(n)}\sum _{\sigma \in S_{k}}\chi _{\uplambda }(\sigma )\sigma =\frac{1}{k!}\sum _{\begin{array}{c} \uplambda \vdash k\\ \ell (\uplambda )\le n \end{array} }\frac{d_{\uplambda }}{D_{\uplambda }(n)}\mathfrak {p}_{\uplambda }. \end{aligned}$$

Write $P_{n,k}$ for the orthogonal projection in $\mathrm {End}((\mathbf {C}^{n})^{\otimes k})$ onto the $\mathsf {U}(n)$-invariant vectors. Henceforth, $\mathrm {tr}$ will always denote the usual trace of a linear map from a finite-dimensional vector space to itself. We have the following proposition of Collins and Śniady [CŚ06, Prop. 2.3].

#### Proposition 2.6

(Collins-Śniady). Let $n,k\in \mathbf {N}$. Suppose $A\in \mathrm {End}((\mathbf {C}^{n})^{\otimes k})$. Then

$$\begin{aligned} P_{n,k}[A]=\rho _{n}^{k}\left( \Phi [A]\cdot \mathrm {Wg}_{n,k}\right) \end{aligned}$$

where

$$\begin{aligned} \Phi [A]{\mathop {=}\limits ^{\mathrm {def}}}\sum _{\sigma \in S_{k}}\mathrm {tr} (A\rho _{n}^{k}(\sigma ^{-1}))\sigma . \end{aligned}$$

### Free groups and surface groups

Recall that $\Sigma _{g}$ is a closed topological surface of genus *g* with base point $x_{0}$ and we have identified $\pi _{1}(\Sigma _{g},x_{0})\cong \Gamma _{g}=\langle a_{1},b_{1},\ldots ,a_{g},b_{g}|[a_{1},b_{1}]\cdots [a_{g},b_{g}]\rangle$.

Given $w\in \mathrm {\mathbf {F}} _{2g}$, we view *w* as a combinatorial word in $a_{1},a_{1}^{-1},b_{1},b_{1}^{-1},\ldots ,a_{g},a_{g}^{-1},b_{g},b_{g}^{-1}$ by writing it in reduced (shortest) form; i.e., $a_{1}$ does not follow $a_{1}^{-1}$, etc.

The commutator subgroup $$[\mathrm {\mathbf {F}} _{2g},\mathrm {\mathbf {F}} _{2g}]$$ (resp. $$[\Gamma _{g},\Gamma _{g}]$$) is the group generated by all elements of the form $$[h_{1},h_{2}]{\mathop {=}\limits ^{\mathrm {def}}}h_{1}h_{2}h_{1}^{-1}h_{2}^{-1}$$ with $$h_{1},h_{2}\in \mathrm {\mathbf {F}} _{2g}$$ (resp. $$\Gamma _{g}$$). A simple fact is that $$w\in [\mathrm {\mathbf {F}} _{2g},\mathrm {\mathbf {F}} _{2g}]$$ if and only if $$a_{i}$$ (resp. $$b_{i})$$ appears the same number of times as $$a_{i}^{-1}$$ (resp. $$b_{i}^{-1}$$) in the reduced word of *w*, for each $$1\le i\le g$$. The abelianization of $$\Gamma _{g}$$ coincides with the first singular homology group of $$\Sigma _{g}$$ and is isomorphic to $$\mathbf {Z}^{2g}$$, generated by the images of $$a_{1},b_{1},\ldots ,a_{g},b_{g}$$. As such, the map induced by $$q_{w}$$ from $$\mathrm {\mathbf {F}} _{2g}$$ to the abelianization of $$\Gamma _{g}$$ has kernel $$[\mathrm {\mathbf {F}} _{2g},\mathrm {\mathbf {F}} _{2g}]$$; hence, $$w\in [\mathrm {\mathbf {F}} _{2g},\mathrm {\mathbf {F}} _{2g}]$$ if and only if $$q_{g}(w)\in [\Gamma _{g},\Gamma _{g}]$$. Therefore, if $$\gamma \in [\Gamma _{g},\Gamma _{g}]$$, any *w* representing $$\gamma $$, or the conjugacy class of $$\gamma ,$$must be in $$[\mathrm {\mathbf {F}} _{2g},\mathrm {\mathbf {F}} _{2g}]$$.

### The Atiyah–Bott–Goldman measure

We first follow Goldman [Gol84] to define the Atiyah-Bott–Goldman measure on $\mathcal {M}_{g,n}$. Consider the word map

$$\begin{aligned} R_{g}&:\mathsf {SU}(n)^{2g}\rightarrow \mathsf {SU}(n)\\ R_{g}&:(A_{1},B_{1},\ldots ,A_{g},B_{g})\mapsto [A_{1},B_{1}]\cdots [A_{g},B_{g}]. \end{aligned}$$

We view $\mathrm {Hom}(\Gamma _{g},\mathsf {SU}(n))$ as $R_{g}^{-1}(\mathrm {id})\subset \mathsf {SU}(n)^{2g}$ via the embedding (1.1). The diagonal action of $\mathsf {SU}(n)$ on $\mathsf {SU}(n)^{2g}$ by conjugation factors through an action of $\mathsf {PSU}(n)$. This action preserves $\mathrm {Hom}(\Gamma _{g},\mathsf {SU}(n))$.

Let $\mathrm {Hom}(\Gamma _{g},\mathsf {SU}(n))^{\mathrm {irr}}$ denote the set of $\phi \in \mathrm {Hom}(\Gamma _{g},\mathsf {SU}(n))$ that are *irreducible *as linear representations*. *The conjugation action of $\mathsf {PSU}(n)$ on $\mathrm {Hom}(\Gamma _{g},\mathsf {SU}(n))^{\mathrm {irr}}$ is clearly free and the action is also proper [Gol84, pg. 205]. Hence,

$$\begin{aligned} \mathcal {M}_{g,n}{\mathop {=}\limits ^{\mathrm {def}}}\mathrm {Hom}(\Gamma _{g},\mathsf {SU}(n))^{\mathrm {irr}}/\mathsf {PSU}(n) \end{aligned}$$

is a smooth real manifold of dimension

$$\begin{aligned} \dim \mathcal {M}_{g,n}=(2g-2)\dim \mathsf {SU}(n) \end{aligned}$$

[Gol84, §1.3]. The complement of $\mathcal {M}_{g,n}$ in $\mathrm {Hom}(\Gamma _{g},\mathsf {SU}(n))/\mathsf {PSU}(n)$ is the union of finitely many manifolds of strictly lower dimension than $\mathcal {M}_{g,n}$.

By [Gol84, §1.4] for $[\phi ]\in \mathcal {M}_{g,n}$, there is an identification of the tangent fiber

2.13 $$\begin{aligned} T_{[\phi ]}(\mathcal {M}_{g,n})\cong H^{1}(\Gamma _{g},\mathrm {Ad}[\phi ]) \end{aligned}$$

where $\mathrm {Ad}[\phi ]$ is $\mathfrak {su}(n)$ with the action of $\Gamma _{g}$ given by composing $\phi$ with the adjoint action of $\mathsf {SU}(n)$ on $\mathfrak {su}(n)$, and $H^{1}(\Gamma _{g},\mathrm {Ad}[\phi ])$ denotes group cohomology with coefficients in a module (see, e.g., [Bro82, Chapt. 3 §1] for a definition of group cohomology with coefficients). A key property of group cohomology of $\Gamma _{g}$ with coefficients in $\mathrm {Ad}[\phi ]$ is that it identifies naturally with the singular cohomology of $\Gamma _{g}$ with coefficients in the local system corresponding to $\mathrm {Ad}[\phi ]$ (for the homology version of this statement see [Bro82, Chapt. 7, Prop. 7.3 and Thm. 7.9, Rmk.]; the cohomology version follows from analogous results).

We normalize the Killing form on $\mathfrak {su}(n)$ so that the induced Riemannian volume on $\mathsf {SU}(n)$ has unit total mass, i.e., it gives the probability Haar measure on $\mathsf {SU}(n)$. Cup product together with the Killing form on $\mathfrak {su}(n)$ and Poincaré duality induces an alternating non-degenerate bilinear form

$$\begin{aligned} H^{1}(\Gamma _{g},\mathrm {Ad}[\phi ])\times H^{1}(\Gamma _{g},\mathrm {Ad}[\phi ])\rightarrow H^{0}(\Gamma _{g},\mathrm {Ad}[\phi ])\cong \mathbf {R}\end{aligned}$$

where the last isomorphism used that $\phi$ is irreducible (see [Gol84, §1.4] for more details on this argument). This yields via (2.13) a differential two-form $\omega _{g,n}^{\mathrm {ABG}}$ on $\mathcal {M}_{g,n}$ that turns out to be closed and hence symplectic ([Gol84, pg. 208]; the closedness of the form was originally proved by Atiyah and Bott [AB83]).

The Atiyah–Bott–Goldman (ABG) measure $\mu _{g,n}^{\mathrm {ABG}}$ is the probability measure on $\mathcal {M}_{g,n}$ induced by the symplectic volume form:

$$\begin{aligned} d\mathrm {Vol}_{\mathcal {M}_{g,n}}{\mathop {=}\limits ^{\mathrm {def}}}\frac{\wedge ^{\frac{1}{2}\dim \mathcal {M}_{g,n}}(\omega _{g,n}^{\mathrm {ABG}})}{(\frac{1}{2}\dim \mathcal {M}_{g,n})!}. \end{aligned}$$

If *f* is a continuous function on $\mathcal {M}_{g,n}$ then the expected value of *f* with respect to $\mu _{g,n}^{\mathrm {ABG}}$ is given by

2.14 $$\begin{aligned} \mathbb {E}_{g,n}[f]{\mathop {=}\limits ^{\mathrm {def}}}\int fd\mu _{g,n}^{\mathrm {ABG}}{\mathop {=}\limits ^{\mathrm {def}}}\frac{\int _{\mathcal {M}_{g,n}}fd\mathrm {Vol}_{\mathcal {M}_{g,n}}}{\int _{\mathcal {M}_{g,n}}d\mathrm {Vol}_{\mathcal {M}_{g,n}}}. \end{aligned}$$

Having defined the ABG measure, we now explain how to deduce Corollary 1.6 from Sengupta’s work [Sen03]. For mostly technical reasons, this involves the introduction of the *heat kernel*^5^ on $\mathsf {SU}(n)$. The heat kernel is a one parameter family of functions $Q_{t}:\mathsf {SU}(n)\rightarrow \mathbf {R}$ for $t\in (0,\infty )$ that, as a function on $(0,\infty )\times \mathsf {SU}(n)$, is a fundamental solution to the heat equation on $\mathsf {SU}(n)$ (cf. [Sen03, §§1.2] for the precise definition). What is important here is that the heat kernel has the expansion

2.15 $$\begin{aligned} Q_{t}(h)=\sum _{\begin{array}{c} {{(\rho ,W)\in \widehat{\mathsf {SU}(n)}}} \end{array} }e^{-\frac{C(\rho )t}{2}}\dim W\mathrm {tr} (\rho (h)) \end{aligned}$$

where $C(\rho )\ge 0$ is the eigenvalue of the Casimir operator of $\mathsf {SU}(n)$ on the representation $(\rho ,W)$. This expansion is uniformly convergent on $\mathsf {SU}(n)$ for each fixed $t>0$ [Bus10, Thm. 7.2.6].

The following theorem was proved by Sengupta [Sen03, Thm. 1].

#### Theorem 2.7

(Sengupta) Let $g\ge 2$ and suppose *f* is a continuous $\mathsf {SU}(n)$-conjugation-invariant function on $\mathsf {SU}(n)^{2g}$, and ${\tilde{f}}$ the function induced by *f* on $\mathcal {M}_{g,n}$. Then

$$\begin{aligned} \lim _{t\rightarrow 0_{+}}\int _{\mathsf {SU}(n)^{2g}}f(x)\overline{Q_{t}(R_{g}(x))}d\mu _{\mathsf {SU}(n)^{2g}}^{\mathrm {Haar}}(x)=\frac{1}{n}\int _{\mathcal {M}_{g,n}}{\tilde{f}}d\mathrm {Vol}_{\mathcal {M}_{g,n}}. \end{aligned}$$

The notation $\lim _{t\rightarrow 0_{+}}$ means the limit is taken along positive values of *t*.

#### Proof

(Deduction of Corollary 1.6 from Theorem 2.7)

Under the same assumptions as Theorem 2.7, using (2.15), one may write for fixed $t>0$

2.16 $$\begin{aligned}&\int _{\mathsf {SU}(n)^{2g}}f(x)\overline{Q_{t}(R_{g}(x))}d\mu _{\mathsf {SU}(n)^{2g}}^{\mathrm {Haar}}(x)\nonumber \\ =&\int _{\mathsf {SU}(n)^{2g}}f(x)\left( \sum _{\begin{array}{c} {{(\rho ,W)\in \widehat{\mathsf {SU}(n)}}} \end{array} }e^{-\frac{C(\rho )t}{2}}\dim W\overline{\mathrm {tr} (\rho (R_{g}(x)))}\right) d\mu _{\mathsf {SU}(n)^{2g}}^{\mathrm {Haar}}\nonumber \\&=\sum _{\begin{array}{c} {{(\rho ,W)\in \widehat{\mathsf {SU}(n)}}} \end{array} }e^{-\frac{C_{\uplambda }(\rho )t}{2}}\dim W\int f(x)\overline{\mathrm {tr} (\rho (R_{g}(x)))}d\mu _{\mathsf {SU}(n)^{2g}}^{\mathrm {Haar}}(x) \end{aligned}$$

where the interchange of sum and integral is valid by uniform convergence of the heat kernel expansion. We are given $\gamma \in \Gamma _{g}$ and $w\in \mathrm {\mathbf {F}} _{2g}$ representing the conjugacy class of $\gamma$. We take $f=\mathrm {tr} \circ w$ (*w* denoting the word map of *w*) so that ${\tilde{f}}=\mathrm {tr} _{\gamma }$. Recall the notation $\mathcal {I}(w,\rho )$ from 1.7, in this case, Theorem 2.7 together with (2.16) gives

2.17 $$\begin{aligned} \frac{1}{n}\int _{\mathcal {M}_{g,n}}\mathrm {tr} _{\gamma }\,d\mathrm {Vol}_{\mathcal {M}_{g,n}}&=\lim _{t\rightarrow 0_{+}}\sum _{\begin{array}{c} {{(\rho ,W)\in \widehat{\mathsf {SU}(n)}}} \end{array} }e^{-\frac{C(\rho )t}{2}}\dim W\,\mathcal {I}(w,\rho ). \end{aligned}$$

Since we assume the hypothesis that $$\sum _{\begin{array}{c} {(\rho ,W)\in \widehat{\mathsf {SU}(n)}} \end{array} }\dim W\,\mathcal {I}(w,\rho )$$ is absolutely convergent, using dominated convergence in (2.17) proves

$$\begin{aligned} \int _{\mathcal {M}_{g,n}}\mathrm {tr} _{\gamma }\,d\mathrm {Vol}_{\mathcal {M}_{g,n}}=n\sum _{\begin{array}{c} {{(\rho ,W)\in \widehat{\mathsf {SU}(n)}}} \end{array} }\dim W\,\mathcal {I}(w,\rho ). \end{aligned}$$

Combining this with Witten’s formula (Theorem 1.5) we obtain Corollary 1.6. $\quad \square$

## Organization of Representations

### Models of representations

In light of Corollary 1.6, we must evaluate $\mathcal {I}(w,\rho )$ for $(\rho ,W)\in \widehat{\mathsf {SU}(n)}$. We use different models of $(\rho ,W)$ throughout the paper: recalling the definition of $\Omega (B;n)$ from (1.9), viewing *B* as fixed and assuming *n* is large enough depending on *B*

- If $(\rho ,W)\in \Omega (B;n)$, we identify $(\rho ,W)\cong (\rho _{n}^{[\mu ,\nu ]},W_{n}^{[\mu ,\nu ]})$ for some $\mu ,\nu$ with $|\mu |,|\nu |\le B^{3}$ uniquely determined by $(\rho ,W)$. Hence,
  $$\begin{aligned} \mathcal {I}(w,\rho )=\mathcal {I}(w,[\mu ,\nu ]){\mathop {=}\limits ^{\mathrm {def}}}\int \mathrm {tr} (w(x))\overline{s_{[\mu ,\nu ]}(R_{g}(x))}d\mu _{\mathsf {SU}(n)^{2g}}^{\mathrm {Haar}}(x). \end{aligned}$$
- If $(\rho ,W)\in \widehat{\mathsf {SU}(n)}\backslash \Omega (B;n)$, then we identify $(\rho ,W)\cong (\rho _{n}^{\uplambda },W_{n}^{\uplambda })$ for some $\uplambda$ with $\ell (\uplambda )\le n-1$ uniquely determined by $(\rho ,W)$. Hence,
  3.1 $$\begin{aligned} \mathcal {I}(w,\rho )=\mathcal {I}(w,\uplambda ){\mathop {=}\limits ^{\mathrm {def}}}\int \mathrm {tr} (w(x))\overline{s_{\uplambda }(R_{g}(x))}d\mu _{\mathsf {SU}(n)^{2g}}^{\mathrm {Haar}}(x). \end{aligned}$$

### Integrating over $\mathsf {SU}(n)^{2g}$ vs $\mathsf {U}(n)^{2g}$

At various points, it is convenient to execute the integral defining ${\mathcal {I}}(w,\uplambda )$ or ${\mathcal {I}}(w,[\mu ,\nu ])$ as an integral over $\mathsf {U}(n)^{2g}$ rather than $\mathsf {SU}(n)^{2g}$. This is indeed possible in all cases that the large-*n* behavior of $\mathbb {E}_{g,n}[\mathrm {tr} _{\gamma }]$ is interesting. Recall that $[\mathrm {\mathbf {F}} _{2g},\mathrm {\mathbf {F}} _{2g}]$ is the commutator subgroup of $\mathrm {\mathbf {F}} _{2g}$.

#### Proposition 3.1

 

1. If $$w\in [\mathrm {\mathbf {F}} _{2g},\mathrm {\mathbf {F}} _{2g}]$$, then
  $$\begin{aligned} {\mathcal {I}}(w,\uplambda )&=\int _{x\in \mathsf {U}(n)^{2g}}\mathrm {tr} (w(x))\overline{s_{\uplambda }(R_{g}(x))}d\mu _{\mathsf {U}(n)^{2g}}^{\mathrm {Haar}}(x),\\ \mathcal {I}(w,[\mu ,\nu ])&=\int _{x\in \mathsf {U}(n)^{2g}}\mathrm {tr} (w(x))\overline{s_{[\mu ,\nu ]}(R_{g}(x))}d\mu _{\mathsf {U}(n)^{2g}}^{\mathrm {Haar}}(x). \end{aligned}$$
  In other words, the integrals can be computed using $\mathsf {U}(n)$ instead of $\mathsf {SU}(n)$.
2. On the other hand, if $w\in \mathrm {\mathbf {F}} _{2g}$ and $w\notin [\mathrm {\mathbf {F}} _{2g},\mathrm {\mathbf {F}} _{2g}]$, then there is $n_{0}=n_{0}(w)$ such that for $n\ge n_{0}$, for any $(\rho ,W)\in \widehat{\mathsf {SU}(n)}$,
  $$\begin{aligned} {\mathcal {I}}(w,\rho )&=0. \end{aligned}$$

#### Proof

By uniqueness of the Haar measure on $\mathsf {U}(n)^{2g}$, we have

3.2 $$\begin{aligned} \mu _{\mathsf {U}(n)^{2g}}^{\mathrm {Haar}}=\mu _{Z(\mathsf {U}(n)^{2g})}^{\mathrm {Haar}}*\mu _{\mathsf {SU}(n)^{2g}}^{\mathrm {Haar}} \end{aligned}$$

where $*$ denotes convolution of measures. Therefore,

$$\begin{aligned}&\int _{x\in \mathsf {U}(n)^{2g}}\mathrm {tr} (w(x))\overline{s_{\uplambda }(R_{g}(x))}d\mu _{\mathsf {U}(n)^{2g}}^{\mathrm {Haar}}(x)\\&\quad = \int _{z\in Z(\mathsf {U}(n)^{2g})}\int _{x\in \mathsf {SU}(n)^{2g}}\mathrm {tr} (w(zx))\overline{s_{\uplambda }(R_{g}(zx))}d\mu _{\mathsf {SU}(n)^{2g}}^{\mathrm {Haar}}(x)d\mu _{Z(\mathsf {U}(n)^{2g})}^{\mathrm {Haar}}(z)\\&\quad = \int _{z\in Z(\mathsf {U}(n)^{2g})}\int _{x\in \mathsf {SU}(n)^{2g}}\mathrm {tr} (w(zx))\overline{s_{\uplambda }(R_{g}(x))}d\mu _{\mathsf {SU}(n)^{2g}}^{\mathrm {Haar}}(x)d\mu _{Z(\mathsf {U}(n)^{2g})}^{\mathrm {Haar}}(z)\\&\quad = \int _{x\in \mathsf {SU}(n)^{2g}}\left( \int _{z\in Z(\mathsf {U}(n)^{2g})}\mathrm {tr} (w(zx))d\mu _{Z(\mathsf {U}(n)^{2g})}^{\mathrm {Haar}}(z)\right) \overline{s_{\uplambda }(R_{g}(x))}d\mu _{\mathsf {SU}(n)^{2g}}^{\mathrm {Haar}}(x). \end{aligned}$$

The first equality used (3.2), the second used $R_{g}(zx)=R_{g}(x)$ for all $x\in \mathsf {U}(n)^{2g}$ and $z\in Z(\mathsf {U}(n)^{2g})$, and the last used Fubini’s theorem. If $w\in [\mathrm {\mathbf {F}} _{2g},\mathrm {\mathbf {F}} _{2g}]$, then by the discussion in §§2.6 the letters of *w* are ‘balanced’ and the inner integrand is $\mathrm {tr} (w(zx))=\mathrm {tr} (w(x))$. Thus, we obtain

$$\begin{aligned} {\mathcal {I}}(w,\uplambda )=\int _{x\in \mathsf {U}(n)^{2g}}\mathrm {tr} (w(x))\overline{s_{\uplambda }(R_{g}(x))}d\mu _{\mathsf {U}(n)^{2g}}^{\mathrm {Haar}}(x). \end{aligned}$$

In other words, the Fourier coefficient can be computed using $\mathsf {U}(n)$ instead of $\mathsf {SU}(n)$. The proof for $\mathcal {I}(w,[\mu ,\nu ])$ is exactly the same. *This proves Part*3.1.

For the second part, suppose *w* is not in $[\mathrm {\mathbf {F}} _{2g},\mathrm {\mathbf {F}} _{2g}]$. Then the letters of *w* are not balanced, and for $z=\left( e\left( \frac{k_{1}}{n}\right) \mathrm {id},\ldots ,e\left( \frac{k_{2g}}{n}\right) \mathrm {id}\right) \in Z(\mathsf {SU}(n)^{2g})$, we have

$$\begin{aligned} \mathrm {tr} (w(zx))=e\left( m_{1}\frac{k_{1}}{n}+\cdots +m_{2g}\frac{k_{2g}}{n}\right) \mathrm {tr} (w(x)) \end{aligned}$$

where all $m_{i}\in \mathbf {Z}$ and *at least one* $m_{i}\ne 0$. On the other hand, $R_{g}(zx)=R_{g}(x)$ as before. Suppose without loss of generality in the following that $m_{1}\ne 0$. Let $z_{0}=\left( e\left( \frac{1}{n}\right) \mathrm {id},\ldots ,\mathrm {id}\right)$. We have by left invariance of Haar measure

$$\begin{aligned} {\mathcal {I}}(w,\rho )&=\int _{x\in \mathsf {SU}(n)^{2g}}\mathrm {tr} (w(x))\overline{\mathrm {tr} (\rho (R_{g}(x)))}d\mu _{\mathsf {SU}(n)^{2g}}^{\mathrm {Haar}}(x)\\&=\int _{x\in \mathsf {SU}(n)^{2g}}\mathrm {tr} (w(z_{0}x))\overline{\mathrm {tr} (\rho (R_{g}(z_{0}x)))}d\mu _{\mathsf {SU}(n)^{2g}}^{\mathrm {Haar}}(x)\\&=e\left( \frac{m_{1}}{n}\right) \int _{x\in \mathsf {SU}(n)^{2g}}\mathrm {tr} (w(x))\overline{\mathrm {tr} (\rho (R_{g}(z_{0}x)))}d\mu _{\mathsf {SU}(n)^{2g}}^{\mathrm {Haar}}(x)\\&=e\left( \frac{m_{1}}{n}\right) \int _{x\in \mathsf {SU}(n)^{2g}}\mathrm {tr} (w(x))\overline{\mathrm {tr} (\rho (R_{g}(x)))}d\mu _{\mathsf {SU}(n)^{2g}}^{\mathrm {Haar}}(x)=e\left( \frac{m_{1}}{n}\right) {\mathcal {I}}(w,\rho ). \end{aligned}$$

Hence, for $n>m_{1}(w)$ we obtain ${\mathcal {I}}(w,\rho )=0$ for any $(\rho ,W)\in \widehat{\mathsf {SU}(n)}$. $\quad \square$

This means the main theorem is proved when $\gamma \notin [\Gamma _{g},\Gamma _{g}]$.

#### Proof of Proposition 1.7

We observe that the series defining $\zeta (2g-2;n)$ converges to a nonzero value when $n\ge 2$ [LL08, Thm. 1.5]. Assume $\gamma \notin [\Gamma _{g},\Gamma _{g}]$. Pick *w* representing the conjugacy class of $\gamma$ as above. Since $\gamma \notin [\Gamma _{g},\Gamma _{g}]$, this implies $w\notin [\mathrm {\mathbf {F}} _{2g},\mathrm {\mathbf {F}} _{2g}]$ by the discussion in §§2.6. Hence, Corollary 1.6 together with Proposition 3.1 Part 3.1 shows

$$\begin{aligned} \mathbb {E}_{g,n}[\mathrm {tr} _{\gamma }]=0 \end{aligned}$$

when $$n\ge n_{0}(w)$$. $$\quad \square $$

*As Theorem* 1.1*has now been proved for* $\gamma \notin [\Gamma _{g},\Gamma _{g}]$, *in the rest of the paper, we may assume* $\gamma \in [\Gamma _{g},\Gamma _{g}]$, *and hence, any* *w* *representing the conjugacy class of* $\gamma$ *is in* $[\mathrm {\mathbf {F}} _{2g},\mathrm {\mathbf {F}} _{2g}]$.

### Rationality of the contribution from $\Omega (B;n)$

Here we prove the following theorem. Let $\mathbf {Q}(t)$ denote the ring of rational functions in an indeterminate *t* with coefficients in $\mathbf {Q}$. For $f\in \mathbf {Q}(t)$ and $t_{0}\in \mathbf {Q}$ we write $f(t_{0})$ for the evaluation of the rational function at $t_{0}$, provided that $t_{0}$ is not a pole of *f*. Let $w\in [\mathrm {\mathbf {F}} _{2g},\mathrm {\mathbf {F}} _{2g}]$ represent the conjugacy class of $\gamma \in [\Gamma _{g},\Gamma _{g}]$.

#### Theorem 3.2

There is a rational function $Q_{B,w}\in \mathbf {Q}(t)$ such that for $n\ge |w|+2B^{3}$

$$\begin{aligned} \sum _{\begin{array}{c} (\rho ,W)\in \Omega (B;n) \end{array} }(\dim W){\mathcal {I}}(w,\rho )=Q_{B,w}(n). \end{aligned}$$

#### Proof

Fix $B\ge 0$. Following the discussion in §§3.1, we have

$$\begin{aligned} \sum _{\begin{array}{c} (\rho ,W)\in \Omega (B;n) \end{array} }(\dim W){\mathcal {I}}(w,\rho )=\sum _{\mu ,\nu \,:\,\ell (\mu ),\ell (\nu )\le B,\mu _{1},\nu _{1}\le B^{2}}D_{[\mu ,\nu ]}(n)\mathcal {I}(w,[\mu ,\nu ]). \end{aligned}$$

Since the index set of the sum is finite and does not depend on *n*, it now suffices to prove that each $D_{[\mu ,\nu ]}(n)\mathcal {I}(w,[\mu ,\nu ])$ agrees with a rational function of *n* for fixed $\mu ,\nu$ as above. By Corollary 2.3, each $D_{[\mu ,\nu ]}(n)$ agrees with a polynomial function of *n* with coefficients in $\mathbf {Q}$ when $n\ge 2B.$ Therefore, the proof of the theorem is reduced to the following:

*Claim: For each* $(\mu ,\nu )\in \Omega (B)$, *when* $n\ge |w|+2B^{3}$, $\mathcal {I}(w,[\mu ,\nu ])$ *agrees with a rational function of* *n* *with coefficients in* $\mathbf {Q}$.

*Proof of claim. *We first use Proposition 3.1 to write

$$\begin{aligned} \mathcal {I}(w,[\mu ,\nu ])=\int _{x\in \mathsf {U}(n)^{2g}}\mathrm {tr} (w(x))\overline{s_{[\mu ,\nu ]}(R_{g}(x))}d\mu _{\mathsf {U}(n)^{2g}}^{\mathrm {Haar}}(x). \end{aligned}$$

For $x\in \mathsf {U}(n)^{2g}$, we use Theorem 2.2 followed by the formula (2.6) to obtain with $k=|\mu |,\ell =|\nu |$

$$\begin{aligned} s_{[\mu ,\nu ]}(R_{g}(x))=\sum _{{{\begin{array}{c} \text {YDs }\mu ^{1},\mu ^{2}\\ |\mu ^{1}|\le \ell ,|\mu ^{2}|\le k \end{array} }}}\alpha _{\mu ^{1},\mu ^{2}}^{\mu ,\nu }p_{\mu ^{1}}(R_{g}(x))p_{\mu ^{2}}(R_{g}(x)^{-1}) \end{aligned}$$

where the $\alpha _{\mu ^{1},\mu ^{2}}^{\mu ,\nu }\in \mathbf {Q}$. Therefore,

$$\begin{aligned} \mathcal {I}(w,[\mu ,\nu ])=\sum _{{{\begin{array}{c} \text {YDs},\mu ^{2}\\ |\mu ^{1}|\le \ell ,|\mu ^{2}|\le k \end{array} }}}\alpha _{\mu ^{1},\mu ^{2}}^{\mu ,\nu }\int _{x\in \mathsf {U}(n)^{2g}}\mathrm {tr} (w(x))p_{\mu ^{1}}(R_{g}(x))p_{\mu ^{2}}(R_{g}(x)^{-1})d\mu _{\mathsf {U}(n)^{2g}}^{\mathrm {Haar}}(x). \end{aligned}$$

For every fixed $\mu ^{1},\mu ^{2}$ appearing in the finite sum above,

3.3 $$\begin{aligned} \int _{x\in \mathsf {U}(n)^{2g}}\mathrm {tr} (w(x))p_{\mu ^{1}}(R_{g}(x))p_{\mu ^{2}}(R_{g}(x)^{-1})d\mu _{\mathsf {U}(n)^{2g}}^{\mathrm {Haar}}(x) \end{aligned}$$

agrees with a rational function of *n* by^6^ [MP19a, Prop. 1.1], for $n\ge |w|+2B^{3}\ge |w|+k+\ell$. This proves the claim and hence the theorem. $\quad \square$

## The Contribution from a Single Family of Representations

### Statement of main sectional result and setup

For $n,\ell \in \mathbf {N}$, let $[n]^{\ell }{\mathop {=}\limits ^{\mathrm {def}}}\underbrace{[n]\times \cdots \times [n]}_{\ell }$; this set has a diagonal action of $S_{n}$ and we write $S_{n}\backslash [n]^{\ell }$ for the quotient set. The main theorem of this $\hbox { \char "0A7}$4 is the following key estimate that will be used for $(\rho _{n}^{\uplambda },W_{n}^{\uplambda })\in \widehat{\mathsf {SU}(n)}\backslash \Omega (B;n)$.

#### Theorem 4.1

For $$w\in [\mathrm {\mathbf {F}} _{2g},\mathrm {\mathbf {F}} _{2g}]$$

$$\begin{aligned} |\mathcal {I}(w,\uplambda )|\le&\sum _{[I]\in S_{_{n}}\backslash [n]^{|w|}}(n)_{\mathfrak {D}(I)}\frac{1}{D_{\uplambda }(n)^{2g}}\sum _{\begin{array}{c} \mu \subset ^{\mathfrak {D}(I)}\uplambda \\ \ell (\mu )\le n-\mathfrak {D}(I) \end{array} }\\&D_{\mu }(n-\mathfrak {D}(I))\left( |\uplambda /\mu |+|w|\right) ^{4g|w|}|\mathcal {SST}_{[n-\mathfrak {D}(I)+1,n]}(\uplambda /\mu )|^{4g} \end{aligned}$$

where for $I=(i_{1},i_{2},\ldots ,i_{|w|})\in [n]^{|w|}$, $\mathfrak {D}(I)$ denotes the number of distinct entries of *I*.

In the rest of this $\hbox { \char "0A7}$4, we assume $g=2$ for simplicity of exposition. The proofs extend in a straightforward way to $g\ge 3$. We write $\{a,b,c,d\}$ for the generators of $\mathrm {\mathbf {F}} _{4}$ and $R=[a,b][c,d]$. We write *w* in reduced form:

4.1 $$\begin{aligned} w=f_{1}^{\varepsilon _{1}}f_{2}^{\varepsilon _{2}}\ldots f_{|w|}^{\varepsilon _{|w|}},\quad \varepsilon _{u}\in \{\pm 1\},\,f_{u}\in \{a,b,c,d\}, \end{aligned}$$

The expression (4.1) implies that for $h{\mathop {=}\limits ^{\mathrm {def}}}(h_{a},h_{b},h_{c},h_{d})\in \mathsf {U}(n)^{4}$

$$\begin{aligned} \mathrm {tr} (w(h))=\sum _{i_{1},\cdots ,i_{|w|}\in [n]}(h_{f_{1}}^{\epsilon _{1}})_{i_{1}i_{2}}(h_{f_{2}}^{\epsilon _{2}})_{i_{2}i_{3}}\cdots (h_{f_{|w|}}^{\epsilon _{|w|}})_{i_{|w|}i_{1}}. \end{aligned}$$

Define for $$I=(i_{1},i_{2},\ldots ,i_{|w|})\in [n]^{|w|}$$

$$\begin{aligned} w_{I}(h){\mathop {=}\limits ^{\mathrm {def}}}(h_{f_{1}}^{\epsilon _{1}})_{i_{1}i_{2}}(h_{f_{2}}^{\epsilon _{2}})_{i_{2}i_{3}}\cdots (h_{f_{|w|}}^{\epsilon _{|w|}})_{i_{|w|}i_{1}}. \end{aligned}$$

After interchanging summation and integration in (3.1), we obtain

4.2 $$\begin{aligned} {\mathcal {I}}(w,\uplambda )&=\sum _{I\in [n]^{|w|}}{\mathcal {I}}^{*}(w_{I},\uplambda ). \end{aligned}$$

where

$$\begin{aligned} \mathcal {I}^{*}(w_{I},\uplambda ){\mathop {=}\limits ^{\mathrm {def}}}\int _{h\in \mathsf {U}(n)^{4}}w_{I}(h)\overline{s_{\uplambda }(R(h))}d\mu _{\mathsf {U}(n)^{4}}^{\mathrm {Haar}}. \end{aligned}$$

Since we have an inclusion $S_{n}\subset \mathsf {U}(n)$ via 0-1 matrices, for $\sigma \in S_{n}$ we can change variables

$$\begin{aligned} (h_{a},h_{b},h_{c},h_{d})\mapsto (h'_{a},h'_{b},h'_{c},h'_{d}){\mathop {=}\limits ^{\mathrm {def}}}(\sigma h_{a}\sigma ^{-1},\sigma h_{b}\sigma ^{-1},\sigma h_{c}\sigma ^{-1},\sigma h_{d}\sigma ^{-1}). \end{aligned}$$

The measure $d\mu _{\mathsf {U}(n)^{4}}^{\mathrm {Haar}}$ is invariant by this change of variables and

$$\begin{aligned} \overline{s_{\uplambda }(R(h'_{a},h'_{b},h'_{c},h'_{d}))}&=\overline{s_{\uplambda }(\sigma R(h{}_{a},h{}_{b},h{}_{c},h{}_{d})\sigma ^{-1})}\\&=\overline{s_{\uplambda }(R(h_{a},h_{b},h_{c},h_{d}))}. \end{aligned}$$

For $I=(i_{1},i_{2},\ldots ,i_{|w|})$ and $\sigma \in S_{n}$, we define $\sigma (I)=(\sigma (i_{1}),\sigma (i_{2}),\ldots ,\sigma (i_{|w|}))$ and we have

$$\begin{aligned} w_{I}(h')=w_{\sigma (I)}(h) \end{aligned}$$

so we obtain in total

$$\begin{aligned} {\mathcal {I}}^{*}(w_{I},\uplambda )=&\int _{h\in \mathsf {U}(n)^{4}}w_{_{I}}(h)\overline{s_{\uplambda }(R(h))}d\mu _{\mathsf {U}(n)^{4}}^{\mathrm {Haar}}\\ =&\int _{h\in \mathsf {U}(n)^{4}}w_{_{\sigma (I)}}(h)\overline{s_{\uplambda }(R(h))}d\mu _{\mathsf {U}(n)^{4}}^{\mathrm {Haar}}\\ =&{\mathcal {I}}^{*}(w_{\sigma (I)},\uplambda ). \end{aligned}$$

We can therefore rewrite (4.2) as

4.3 $$\begin{aligned} {\mathcal {I}}(w,\uplambda )&=\sum _{[I]\in S_{_{n}}\backslash [n]^{|w|}}|S_{n}.I|{\mathcal {I}}^{*}(w_{I},\uplambda )\nonumber \\&=\sum _{[I]\in S_{_{n}}\backslash [n]^{|w|}}(n)_{\mathfrak {D}(I)}{\mathcal {I}}^{*}(w_{I},\uplambda ) \end{aligned}$$

where $\mathfrak {D}(I)$ is the number of distinct entries in *I*. Most of the rest of the section will be devoted to estimating ${\mathcal {I}}^{*}(w_{I},\uplambda )$; the point of the previous calculations is that we can assume $I\in [n-\mathfrak {D}(I)+1,n]^{|w|}$ and this will be exploited in §§4.2.

### First integrating over a large subgroup

We keep the assumptions of the previous section and also assume

$$\begin{aligned} I\in [n-\mathfrak {D}(I)+1,n]^{|w|}. \end{aligned}$$

We fix *I* and hence write $\mathfrak {D}=\mathfrak {D}(I)$. This assumption means that the function $w_{I}:\mathsf {U}(n)^{4}\rightarrow \mathbf {C}$ is bi-invariant for $\mathsf {U}(m)^{4}$ where

$$\begin{aligned} m{\mathop {=}\limits ^{\mathrm {def}}}n-\mathfrak {D}. \end{aligned}$$

To simplify notation, *all integrals over groups are done with respect to the probability Haar measure* and this will be denoted by *dg* where *g* is the group element.

Our goal is to calculate ${\mathcal {I}}^{*}(w_{I},\uplambda )$. The bi-invariance of $d\mu _{\mathsf {U}(n)^{4}}^{\mathrm {Haar}}$ and the $\mathsf {U}(m)^{4}$-bi-invariance of $w_{I}$ means we can write

4.4 $$\begin{aligned} {\mathcal {I}}^{*}(w_{I},\uplambda )&=\int _{h\in \mathsf {U}(n)^{4}}w_{_{I}}(h)\overline{s_{\uplambda }(R(h))}dh\nonumber \\&=\int _{h_{1},h_{2}\in \mathsf {U}(m)^{4}}\left( \int _{h\in \mathsf {U}(n)^{4}}w_{_{I}}(h_{1}hh_{2})\overline{s_{\uplambda }(R(h_{1}hh_{2}))}dh\right) dh_{1}dh_{2}\nonumber \\&=\int _{h_{1},h_{2}\in \mathsf {U}(m)^{4}}\int _{h\in \mathsf {U}(n)^{4}}w_{_{I}}(h)\overline{s_{\uplambda }(R(h_{1}hh_{2}))}dh\,dh_{1}\,dh_{2}\nonumber \\&=\int _{h\in \mathsf {U}(n)^{4}}w_{I}(h)\left( \int _{h_{1},h_{2}\in \mathsf {U}(m)^{4}}\overline{s_{\uplambda }(R(h_{1}hh_{2}))}dh_{1}dh_{2}\right) dh. \end{aligned}$$

The first cancelation we will obtain comes from the integral

4.5 $$\begin{aligned} \int _{h_{1},h_{2}\in \mathsf {U}(m)^{4}}\overline{s_{\uplambda }(R(h_{1}hh_{2}))}dh_{1}dh_{2},\quad h\in \mathsf {U}(n)^{4}. \end{aligned}$$

Our approach to this integral follows the same lines as [MP20, §4.4]. We consider the vector space

4.6 $$\begin{aligned} \mathcal {W}_{R}^{\uplambda }{\mathop {=}\limits ^{\mathrm {def}}}W_{a}^{\uplambda }\otimes \check{W_{a}^{\uplambda }}\otimes W_{b}^{\uplambda }\otimes \check{W_{b}^{\uplambda }}\otimes W_{c}^{\uplambda }\otimes \check{W_{c}^{\uplambda }}\otimes W_{d}^{\uplambda }\otimes \check{W_{d}^{\uplambda }} \end{aligned}$$

which is a unitary representation of $\mathsf {U}(n)^{4}$, the subscripts indicating which elements of $(h_{a},h_{b},h_{c},h_{d})$ act on which factor, so each $h_{f}$ acts diagonally on two factors.

Let $B_{\uplambda }\in \mathrm {End}(\mathcal {W}_{R}^{\uplambda })$ be defined via matrix coefficients by the formula

4.7 $$\begin{aligned}&\left\langle B_{\uplambda }\left( v_{1}\otimes {\check{v}}_{2}\otimes v_{3}\otimes \check{v_{4}}\otimes v_{5}\otimes {\check{v}}_{6}\otimes v_{7}\otimes \check{v_{8}}\right) ,w_{1}\otimes {\check{w}}_{2}\otimes w_{3}\otimes \check{w_{4}}\right. \nonumber \\&\quad \left. \otimes w_{5}\otimes {\check{w}}_{6}\otimes w_{7}\otimes \check{w_{8}}\right\rangle {\mathop {=}\limits ^{\mathrm {def}}}\nonumber \\&~~~~~~~~~\left\langle v_{5},w_{7}\right\rangle \left\langle w_{5},w_{8}\right\rangle \left\langle w_{6},v_{8}\right\rangle \left\langle v_{3},v_{6}\right\rangle \left\langle v_{1},w_{3}\right\rangle \left\langle w_{1},w_{4}\right\rangle \left\langle w_{2},v_{4}\right\rangle \left\langle v_{7},v_{2}\right\rangle . \end{aligned}$$

We have the following lemma analogous to [MP20, Lemma 4.7].

#### Lemma 4.2

For any $h=(h_{a},h_{b},h_{c},h_{d})\in \mathsf {U}(n)^{4}$, we have

$$\begin{aligned} \mathrm {tr}_{\mathcal {W}_{R}^{\uplambda }}\left( B_{\uplambda }\circ (h_{a},h_{b},h_{c},h_{d})\right) =\overline{s_{\uplambda }\left( [h_{a},h_{b}][h_{c},h_{d}]\right) }=s_{\uplambda }([h_{d},h_{c}][h_{b},h_{a}]). \end{aligned}$$

The purpose of Lemma 4.2 is that it turns the integral in (4.5) into a *projection operator. *Indeed, let *Q* denote the orthogonal projection in $\mathcal {W}_{R}^{\uplambda }$ onto the $\mathsf {U}(m)^{4}$-invariant vectors.

#### Lemma 4.3

We have

$$\begin{aligned} \int _{h_{1},h_{2}\in \mathsf {U}(m)^{4}}\overline{s_{\uplambda }(R(h_{1}hh_{2}))}dh_{1}dh_{2}&=\mathrm {tr}_{\mathcal {W}_{R}^{\uplambda }}\left( hQB_{\uplambda }Q\right) \end{aligned}$$.

#### Proof

By Lemma 4.2

$$\begin{aligned} \int _{h_{1},h_{2}\in \mathsf {U}(m)^{4}}\overline{s_{\uplambda }(R(h_{1}hh_{2}))}dh_{1}dh_{2}&=\int _{h_{1},h_{2}\in \mathsf {U}(m)^{4}}\mathrm {tr}_{\mathcal {W}_{R}^{\uplambda }}\left( B_{\uplambda }\circ h_{1}hh_{2}\right) dh_{1}dh_{2}\\&=\mathrm {tr}_{\mathcal {W}_{R}^{\uplambda }}\left( B_{\uplambda }\circ \left( \int _{h_{1}\in \mathsf {U}(m)^{4}}h_{1}\right) h\left( \int _{h_{2}\in \mathsf {U}(m)^{4}}h_{2}\right) \right) \\&=\mathrm {tr}_{\mathcal {W}_{R}^{\uplambda }}\left( B_{\uplambda }QhQ\right) =\mathrm {tr}_{\mathcal {W}_{R}^{\uplambda }}\left( hQB_{\uplambda }Q\right) . \end{aligned}$$

$$\square $$

Recalling the definition of $\mathcal {E}_{R_{1},R_{2}}^{\uplambda ,\mu ,m}$ from (2.10), we are able to calculate $\mathrm {tr}_{\mathcal {W}_{R}^{\uplambda }}\left( hQB_{\uplambda }Q\right)$ as follows.

#### Proposition 4.4

We have

$$\begin{aligned} \mathrm {tr}_{\mathcal {W}_{R}^{\uplambda }}\left( hQB_{\uplambda }Q\right)&=\sum _{\begin{array}{c} \mu \subset ^{\mathfrak {D}}\uplambda \\ \ell (\mu )\le m \end{array} }\frac{1}{D_{\mu }(m)^{3}}\sum _{S_{1},S_{2},S_{3},S_{4},T_{2},T_{4},s_{1},s_{2}\in \mathcal {SST}_{[m+1,n]}(\uplambda /\mu )}\\&\langle h[\mathcal {E}_{s_{1},T_{2}}^{\uplambda ,\mu ,m}\otimes \mathcal {E}_{S_{1},s_{1}}^{\uplambda ,\mu ,m}\otimes \mathcal {E}_{s_{2},T_{4}}^{\uplambda ,\mu ,m}\otimes \mathcal {E}_{S_{3},s_{2}}^{\uplambda ,\mu ,m}],\mathcal {E}_{S_{1},S_{4}}^{\uplambda ,\mu ,m}\otimes \mathcal {E}_{S_{2},T_{2}}^{\uplambda ,\mu ,m}\\&\qquad \otimes \mathcal {E}_{S_{3},S_{2}}^{\uplambda ,\mu ,m}\otimes \mathcal {E}_{S_{4},T_{4}}^{\uplambda ,\mu ,m}\rangle . \end{aligned}$$

#### Proof

An orthonormal basis for the $\mathsf {U}(m)^{4}$-invariant vectors in $\mathcal {W}_{R}^{\uplambda }$ is given by

$$\begin{aligned} \mathcal {E}_{S_{1},T_{1}}^{\uplambda ,\mu _{1},m}\otimes \mathcal {E}_{S_{2},T_{2}}^{\uplambda ,\mu _{2},m}\otimes \mathcal {E}_{S_{3},T_{3}}^{\uplambda ,\mu _{3},m}\otimes \mathcal {E}_{S_{4},T_{4}}^{\uplambda ,\mu _{4},m} \end{aligned}$$

where the $\mu _{i}$ range over all $\mu _{i}\subset ^{\mathfrak {D}}\uplambda$ with $\ell (\mu _{i})\le m$ and each $S_{i},T_{i}\in \mathcal {SST}_{[m+1,n]}(\uplambda /\mu _{i}).$ This can be extended to a full orthonormal basis of $\mathcal {W}_{R}^{\uplambda }$, and hence,

4.8 $$\begin{aligned} \mathrm {tr}_{\mathcal {W}_{R}^{\uplambda }}\left( hQB_{\uplambda }Q\right) =&\sum _{\begin{array}{c} \mu _{i}\subset ^{\mathfrak {D}}\uplambda \\ \ell (\mu _{i})\le m \end{array} }\sum _{S_{i},T_{i}\in \mathcal {SST}_{[m+1,n]}(\uplambda /\mu _{i})}\langle hQB_{\uplambda }\mathcal {E}_{S_{1},T_{1}}^{\uplambda ,\mu _{1},m}\otimes \mathcal {E}_{S_{2},T_{2}}^{\uplambda ,\mu _{2},m}\otimes \mathcal {E}_{S_{3},T_{3}}^{\uplambda ,\mu _{3},m}\nonumber \\&\otimes \mathcal {E}_{S_{4},T_{4}}^{\uplambda ,\mu _{4},m}, \mathcal {E}_{S_{1},T_{1}}^{\uplambda ,\mu _{1},m}\otimes \mathcal {E}_{S_{2},T_{2}}^{\uplambda ,\mu _{2},m}\otimes \mathcal {E}_{S_{3},T_{3}}^{\uplambda ,\mu _{3},m}\otimes \mathcal {E}_{S_{4},T_{4}}^{\uplambda ,\mu _{4},m}\rangle . \end{aligned}$$

The matrix coefficient

$$\begin{aligned}&\langle hQB_{\uplambda }\mathcal {E}_{S_{1},T_{1}}^{\uplambda ,\mu _{1},m}\otimes \mathcal {E}_{S_{2},T_{2}}^{\uplambda ,\mu _{2},m}\otimes \mathcal {E}_{S_{3},T_{3}}^{\uplambda ,\mu _{3},m}\otimes \mathcal {E}_{S_{4},T_{4}}^{\uplambda ,\mu _{4},m},\mathcal {E}_{S_{1},T_{1}}^{\uplambda ,\mu _{1},m}\\&\quad \otimes \mathcal {E}_{S_{2},T_{2}}^{\uplambda ,\mu _{2},m}\otimes \mathcal {E}_{S_{3},T_{3}}^{\uplambda ,\mu _{3},m}\otimes \mathcal {E}_{S_{4},T_{4}}^{\uplambda ,\mu _{4},m}\rangle \end{aligned}$$

will now be calculated in stages. Firstly

$$\begin{aligned}&B_{\uplambda }\mathcal {E}_{S_{1},T_{1}}^{\uplambda ,\mu _{1},m}\otimes \mathcal {E}_{S_{2},T_{2}}^{\uplambda ,\mu _{2},m}\otimes \mathcal {E}_{S_{3},T_{3}}^{\uplambda ,\mu _{3},m}\otimes \mathcal {E}_{S_{4},T_{4}}^{\uplambda ,\mu _{4},m}\\&\quad = \frac{1}{\sqrt{D_{\mu _{1}}(m)D_{\mu _{2}}(m)D_{\mu _{3}}(m)D_{\mu _{4}}(m)}}B_{\uplambda }\\&\qquad \sum _{R_{i}\in \mathcal {SST}_{[m]}(\mu _{i})}w_{R_{1}\sqcup S_{1}}\otimes {\check{w}}_{R_{1}\sqcup T_{1}}\otimes w_{R_{2}\sqcup S_{2}}\otimes {\check{w}}_{R_{2}\sqcup T_{2}}\\&\qquad \quad \otimes w_{R_{3}\sqcup S_{3}}\otimes {\check{w}}_{R_{3}\sqcup T_{3}}\otimes w_{R_{4}\sqcup S_{4}}\otimes {\check{w}}_{R_{4}\sqcup T_{4}}\\&\quad = \frac{1}{\sqrt{D_{\mu _{1}}(m)D_{\mu _{2}}(m)D_{\mu _{3}}(m)D_{\mu _{4}}(m)}}\\&\qquad \sum _{R_{i}\in \mathcal {SST}_{[m]}(\mu _{i})}{\mathbf {1}}\{R_{2}\sqcup S_{2}=R_{3}\sqcup T_{3},R_{1}\sqcup T_{1}=R_{4}\sqcup S_{4}\}\\&\qquad \sum _{t_{1},t_{2}\in \mathcal {SST}_{[n]}(\uplambda )}w_{t_{1}}\otimes {\check{w}}_{R_{2}\sqcup T_{2}}\otimes w_{R_{1}\sqcup S_{1}}\otimes {\check{w}}_{t_{1}}\otimes w_{t_{2}}\otimes {\check{w}}_{R_{4}\sqcup T_{4}}\otimes w_{R_{3}\sqcup S_{3}}\otimes {\check{w}}_{t_{2}}. \end{aligned}$$

We have

$$\begin{aligned}&Q[w_{t_{1}}\otimes {\check{w}}_{R_{2}\sqcup T_{2}}\otimes w_{R_{1}\sqcup S_{1}}\otimes {\check{w}}_{t_{1}}\otimes w_{t_{2}}\otimes {\check{w}}_{R_{4}\sqcup T_{4}}\otimes w_{R_{3}\sqcup S_{3}}\otimes {\check{w}}_{t_{2}}]\\&\quad = \frac{{\mathbf {1}}\{t_{1}|_{\le m}=R_{2}=R_{1},t_{2}|_{\le m}=R_{4}=R_{3}\}}{\sqrt{D_{\mu _{1}}(m)D_{\mu _{2}}(m)D_{\mu _{3}}(m)D_{\mu _{4}}(m)}}\\&\qquad \mathcal {E}_{t_{1}|_{>m},T_{2}}^{\uplambda ,\mu _{2},m}\otimes \mathcal {E}_{S_{1},t_{1}|_{>m}}^{\uplambda ,\mu _{1},m}\otimes \mathcal {E}_{t_{2}|_{>m},T_{4}}^{\uplambda ,\mu _{4},m}\otimes \mathcal {E}_{S_{3},t_{2}|_{>m}}^{\uplambda ,\mu _{3},m}. \end{aligned}$$

Combining the previous two calculations, we obtain

$$\begin{aligned}&QB_{\uplambda }[\mathcal {E}_{S_{1},T_{1}}^{\uplambda ,\mu _{1},m}\otimes \mathcal {E}_{S_{2},T_{2}}^{\uplambda ,\mu _{2},m}\otimes \mathcal {E}_{S_{3},T_{3}}^{\uplambda ,\mu _{3},m}\otimes \mathcal {E}_{S_{4},T_{4}}^{\uplambda ,\mu _{4},m}]\\&\quad = \frac{1}{D_{\mu _{1}}(m)D_{\mu _{2}}(m)D_{\mu _{3}}(m)D_{\mu _{4}}(m)}\\&\qquad \sum _{R_{i}\in \mathcal {SST}_{[m]}(\mu _{i})}{\mathbf {1}}\{R_{2}\sqcup S_{2}=R_{3}\sqcup T_{3},R_{1}\sqcup T_{1}=R_{4}\sqcup S_{4}\}\\&\qquad \sum _{t_{1},t_{2}\in \mathcal {SST}_{[n]}(\uplambda )}{\mathbf {1}}\{t_{1}|_{\le m}=R_{2}=R_{1},t_{2}|_{\le m}=R_{4}=R_{3}\}\mathcal {E}_{t_{1}|_{>m},T_{2}}^{\uplambda ,\mu _{2},m}\otimes \mathcal {E}_{S_{1},t_{1}|_{>m}}^{\uplambda ,\mu _{1},m}\\&\qquad \otimes \mathcal {E}_{t_{2}|_{>m},T_{4}}^{\uplambda ,\mu _{4},m}\otimes \mathcal {E}_{S_{3},t_{2}|_{>m}}^{\uplambda ,\mu _{3},m}\\&\quad = \frac{{\mathbf {1}}\{S_{2}=T_{3},T_{1}=S_{4},\mu _{1}=\mu _{2}=\mu _{3}=\mu _{4}\}}{D_{\mu _{1}}(m)^{3}}\sum _{s_{1},s_{2}\in \mathcal {SST}_{[m+1,n]}(\uplambda /\mu _{1})}\\&\qquad \mathcal {E}_{s_{1},T_{2}}^{\uplambda ,\mu _{2},m}\otimes \mathcal {E}_{S_{1},s_{1}}^{\uplambda ,\mu _{1},m}\otimes \mathcal {E}_{s_{2},T_{4}}^{\uplambda ,\mu _{4},m}\otimes \mathcal {E}_{S_{3},s_{2}}^{\uplambda ,\mu _{3},m}. \end{aligned}$$

Therefore, from (4.8)

$$\begin{aligned} \mathrm {tr}_{\mathcal {W}_{R}^{\uplambda }}\left( hQB_{\uplambda }Q\right) =&\sum _{\begin{array}{c} \mu \subset ^{\mathfrak {D}}\uplambda \\ \ell (\mu )\le m \end{array} }\frac{1}{D_{\mu }(m)^{3}}\\&\quad \sum _{S_{i},T_{i}\in \mathcal {SST}_{[m+1,n]}(\uplambda /\mu )}{\mathbf {1}}\{S_{2}=T_{3},T_{1}=S_{4}\}\sum _{s_{1},s_{2}\in \mathcal {SST}_{[m+1,n]}(\uplambda /\mu )}\\&\quad \langle h[\mathcal {E}_{s_{1},T_{2}}^{\uplambda ,\mu ,m}\otimes \mathcal {E}_{S_{1},s_{1}}^{\uplambda ,\mu ,m}\otimes \mathcal {E}_{s_{2},T_{4}}^{\uplambda ,\mu ,m}\otimes \mathcal {E}_{S_{3},s_{2}}^{\uplambda ,\mu ,m}],\mathcal {E}_{S_{1},T_{1}}^{\uplambda ,\mu ,m}\otimes \mathcal {E}_{S_{2},T_{2}}^{\uplambda ,\mu ,m}\\&\quad \otimes \mathcal {E}_{S_{3},T_{3}}^{\uplambda ,\mu ,m}\otimes \mathcal {E}_{S_{4},T_{4}}^{\uplambda ,\mu ,m}\rangle . \end{aligned}$$

$$\square $$

Now combining (4.4), Lemma 4.3, and Proposition 4.4, we obtain:

#### Proposition 4.5

For $$I\in [m+1,n]^{|w|}$$

$$\begin{aligned} {\mathcal {I}}^{*}(w_{I},\uplambda )&=\sum _{\begin{array}{c} \mu \subset ^{\mathfrak {D}}\uplambda \\ \ell (\mu )\le m \end{array} }\frac{1}{D_{\mu }(m)^{3}}\sum _{S_{1},S_{2},S_{3},S_{4},T_{2},T_{4},s_{1},s_{2}\in \mathcal {SST}_{[m+1,n]}(\uplambda /\mu )}\\&\int _{h\in \mathsf {U}(n)^{4}}w_{I}(h) \langle h[\mathcal {E}_{s_{1},T_{2}}^{\uplambda ,\mu ,m}\otimes \mathcal {E}_{S_{1},s_{1}}^{\uplambda ,\mu ,m}\otimes \mathcal {E}_{s_{2},T_{4}}^{\uplambda ,\mu ,m}\otimes \mathcal {E}_{S_{3},s_{2}}^{\uplambda ,\mu ,m}],\mathcal {E}_{S_{1},T_{1}}^{\uplambda ,\mu ,m}\\&\quad \otimes \mathcal {E}_{S_{2},T_{2}}^{\uplambda ,\mu ,m}\otimes \mathcal {E}_{S_{3},T_{3}}^{\uplambda ,\mu ,m}\otimes \mathcal {E}_{S_{4},T_{4}}^{\uplambda ,\mu ,m}\rangle dh. \end{aligned}$$

This is progress because now the integrand is $\mathsf {U}(m)^{4}$-bi-invariant, and hence, the integral is now essentially over $\mathsf {U}(m)^{4}\backslash \mathsf {U}(n)^{4}/\mathsf {U}(m)^{4}$ rather than the full $\mathsf {U}(n)^{4}$. In the process of integrating over $\mathsf {U}(m)^{4}$, by exploiting the structure of the relator *R* of $\Gamma _{g}$ we picked up a helpful factor of $\frac{1}{D_{\mu }(m)^{3}}$ and forced $\mu$ to be the same in each tensor factor of the equation stated in Proposition 4.5. We show how to proceed further in the next section.

### Second integration: strategy

The only method we really have to proceed from Proposition 4.5 is to use the Weingarten calculus from §§2.5. The caveat is that this works well when integrating functions that are finite products of matrix coefficients in the standard representation of $\mathsf {U}(n)$. Here we are concerned with the integral

4.9 $$\begin{aligned}&\int _{h\in \mathsf {U}(n)^{4}}w_{I}(h)\langle h[\mathcal {E}_{s_{1},T_{2}}^{\uplambda ,\mu ,m}\otimes \mathcal {E}_{S_{1},s_{1}}^{\uplambda ,\mu ,m}\otimes \mathcal {E}_{s_{2},T_{4}}^{\uplambda ,\mu ,m}\otimes \mathcal {E}_{S_{3},s_{2}}^{\uplambda ,\mu ,m}],\mathcal {E}_{S_{1},T_{1}}^{\uplambda ,\mu ,m}\nonumber \\&\qquad \otimes \mathcal {E}_{S_{2},T_{2}}^{\uplambda ,\mu ,m}\otimes \mathcal {E}_{S_{3},T_{3}}^{\uplambda ,\mu ,m}\otimes \mathcal {E}_{S_{4},T_{4}}^{\uplambda ,\mu ,m}\rangle dh \end{aligned}$$

appearing in Proposition 4.5. So to use the Weingarten calculus we must write the integrand of (4.9) as a product of matrix coefficients in some $\mathrm {End}((\mathbf {C}^{n})^{\otimes K})$.

Since we assume $w\in [\mathrm {\mathbf {F}} _{4},\mathrm {\mathbf {F}} _{4}]$, by the observation about the exponents of letters in the reduced word of *w* from §§2.6, (4.9) is equal to the product of four independent integrals over $\mathsf {U}(n)$ of the form

4.10 $$\begin{aligned} \int _{\mathsf {U}(n)}h_{i_{1}j_{1}}\cdots h_{i_{p}j_{p}}{\bar{h}}_{i'_{1}j'_{1}}\cdots {\bar{h}}_{i'_{p}j'_{p}}\langle h[\mathcal {E}_{s,t}^{\uplambda ,\mu ,m}],\mathcal {E}_{s',t'}^{\uplambda ,\mu ,m}\rangle dh, \end{aligned}$$

one for each *a*, *b*, *c*, *d*, and where

4.11 $$\begin{aligned} i_{k},j_{k},i'_{k},j'_{k}&\in [m+1,n],\quad \forall k\in [p]\nonumber \\ s,s',t,t'&\in \mathcal {SST}_{[m+1,n]}(\uplambda /\mu ). \end{aligned}$$

So we will estimate (4.10) under the assumptions (4.11) and later take the product of the four resulting bounds to estimate (4.9).

We now outline the strategy that we will follow to estimate the integral (4.10).

1. We can write
  $$\begin{aligned} h_{i_{1}j_{1}}\cdots h_{i_{p}j_{p}}{\bar{h}}_{i'_{1}j'_{1}}\cdots {\bar{h}}_{i'_{p}j'_{p}}=\langle \pi _{n}^{p,p}(h)X,X'\rangle \end{aligned}$$
  where *X*, $X'\in (\mathbf {C}^{n})^{\otimes p}\otimes (\check{\mathbf {C}^{n}})^{\otimes p}$ are pure tensors of the standard basis elements and dual standard basis elements; moreover, they only involve
  $$\begin{aligned} e_{k},{\check{e}}_{\ell },\quad k,l\in [m+1,n]. \end{aligned}$$
  This means that
  $$\begin{aligned} h_{i_{1}j_{1}}\cdots h_{i_{p}j_{p}}{\bar{h}}_{i'_{1}j'_{1}}\cdots {\bar{h}}_{i'_{p}j'_{p}}\langle h[\mathcal {E}_{s,t}^{\uplambda ,\mu ,m}],\mathcal {E}_{s',t'}^{\uplambda ,\mu ,m}\rangle =\langle h[\mathcal {E}_{s,t}^{\uplambda ,\mu ,m}\otimes X],\mathcal {E}_{s',t'}^{\uplambda ,\mu ,m}\otimes X'\rangle . \end{aligned}$$
  The implication for this to our key integral (4.10) is that
  $$\begin{aligned} (4.10)=\langle P[\mathcal {E}_{s,t}^{\uplambda ,\mu ,m}\otimes X],\mathcal {E}_{s',t'}^{\uplambda ,\mu ,m}\otimes X'\rangle =\langle P[\mathcal {E}_{s,t}^{\uplambda ,\mu ,m}\otimes X],P[\mathcal {E}_{s',t'}^{\uplambda ,\mu ,m}\otimes X']\rangle \end{aligned}$$
  where *P* is the self-adjoint orthogonal projection on $\mathrm {End}(V^{\uplambda })\otimes (\mathbf {C}^{n})^{\otimes p}\otimes (\check{\mathbf {C}^{n}})^{\otimes p}$ onto the $\mathsf {U}(n)$-invariant vectors. Here, we accept some loss of accuracy by using the Schwarz inequality to deduce
  4.12 $$\begin{aligned} |(4.10)|\le \Vert P[\mathcal {E}_{s,t}^{\uplambda ,\mu ,m}\otimes X]\Vert \Vert P[\mathcal {E}_{s',t'}^{\uplambda ,\mu ,m}\otimes X']\Vert . \end{aligned}$$
2. For any $s,t\in \mathcal {SST}_{[m+1,n]}(\uplambda /\mu )$ we will construct a vector $A_{s,t}^{\uplambda ,\mu }\in \mathrm {End}((\mathbf {C}^{n})^{\otimes B})$ in §§4.4 such that under the Schur–Weyl intertwiner (cf. Proposition 2.4) we have
  $$\begin{aligned} (\mathcal {F}\otimes {\check{\mathcal {F}}})[A_{s,t}^{\uplambda ,\mu }]=\mathcal {E}_{s,t}^{\uplambda ,\mu ,m}\otimes z_{s,t}^{\uplambda ,\mu }\in \mathrm {End}(W^{\uplambda })\otimes \mathrm {End}(V^{\uplambda }), \end{aligned}$$
  for some $z_{s,t}^{\uplambda ,\mu }\in \mathrm {End}(V^{\uplambda })$. This property is established in Proposition 4.7.
3. In Proposition 4.8, we calculate $\Vert A_{s,t}^{\uplambda ,\mu }\Vert$. As $\mathcal {E}_{s,t}^{\uplambda ,\mu ,m}$ has unit norm, this is the same as $\Vert z_{s,t}^{\uplambda ,\mu }\Vert$.
4. We now calculate ${\hat{P}}[A_{s,t}^{\uplambda ,\mu }\otimes X]$ where ${\hat{P}}$ is the projection onto the $\mathsf {U}(n)$-invariant vectors in $\mathrm {End}((\mathbf {C}^{n})^{\otimes B})\otimes (\mathbf {C}^{n})^{\otimes p}\otimes (\check{\mathbf {C}^{n}})^{\otimes p}$. We are able to do this using the Weingarten calculus; this is the reason for the construction of $A_{s,t}^{\uplambda ,\mu }$. This calculation takes place in §§4.6.
5. We estimate $\Vert {\hat{P}}[A_{s,t}^{\uplambda ,\mu }\otimes X]\Vert$ in Proposition 4.9.
6. On the other hand, after suitable identifications, we have
  $$\begin{aligned} (\mathcal {F}\otimes {\check{\mathcal {F}}}\otimes \mathrm {Id}_{(\mathbf {C}^{n})^{\otimes p}\otimes (\check{\mathbf {C}^{n}})^{\otimes p}})[{\hat{P}}[A_{s,t}^{\uplambda ,\mu }\otimes X]]=P[\mathcal {E}_{s,t}^{\uplambda ,\mu ,m}\otimes X]\otimes z_{s,t}^{\uplambda ,\mu }, \end{aligned}$$
  and hence,
  4.13 $$\begin{aligned} \Vert P[\mathcal {E}_{s,t}^{\uplambda ,\mu ,m}\otimes X]\Vert =\frac{\Vert {\hat{P}}[A_{s,t}^{\uplambda ,\mu }\otimes X]\Vert }{\Vert A_{s,t}^{\uplambda ,\mu }\Vert }. \end{aligned}$$
  Since we have calculated the denominator on the right-hand side above, and bounded the numerator, we have bounded $\Vert P[\mathcal {E}_{s,t}^{\uplambda ,\mu ,m}\otimes X]\Vert$. We can use exactly the same method to bound $\Vert P[\mathcal {E}_{s',t'}^{\uplambda ,\mu ,m}\otimes X']\Vert$, and putting these estimates into (4.12), we obtain an upper bound on |(4.10) |, as desired.

#### Remark 4.6

It does not seem possible to exactly evaluate (4.9) using this method. Indeed one might hope that

$$\begin{aligned} (4.9)=\frac{\langle {\hat{P}}[A_{s,t}^{\uplambda ,\mu }\otimes X],{\hat{P}}[A_{s',t'}^{\uplambda ,\mu }\otimes X']\rangle }{\Vert A_{s,t}^{\uplambda ,\mu }\Vert \Vert A_{s',t'}^{\uplambda ,\mu }\Vert }. \end{aligned}$$

The construction of $A_{s,t}^{\uplambda ,\mu },A_{s',t'}^{\uplambda ,\mu }$ we give here means that the numerator above does not in general coincide with $\langle P[\mathcal {E}_{s,t}^{\uplambda ,\mu ,m}\otimes X],P[\mathcal {E}_{s',t'}^{\uplambda ,\mu ,m}\otimes X']\rangle$. The use of the Schwarz inequality bypasses this problem, at the cost of introducing an inequality.

The components of this strategy will be carried out in the following sections.

### Construction of $A_{s,t}^{\uplambda ,\mu }$

Now fix $m,\mathfrak {D},b,B\in \mathbf {N}_{0}$, $n=m+\mathfrak {D}$, $b\le B$. Also fix $\uplambda \vdash B$ and $\mu \vdash b$ with $\ell (\uplambda )\le n$ and $\mu \subset ^{\mathfrak {D}}\uplambda$ with $\ell (\mu )\le m$. We also fix

$$\begin{aligned} s,t\in \mathcal {SST}_{[m+1,n]}(\uplambda /\mu ). \end{aligned}$$

From these data, we will construct a special vector in $(\mathbf {C}^{n})^{\otimes B}\otimes (\check{\mathbf {C}^{n}})^{\otimes B}\cong \mathrm {End}((\mathbf {C}^{n})^{\otimes B})$. This will be done in stages.

Recall the weight functions $\omega _{s},\omega _{t}:[m+1,n]\rightarrow \mathbf {N}_{0}$ from $\hbox { \char "0A7}$2.1. For $z\in \mathbf {C}[S_{k}]$, we will write $\rho _{n}^{k}(z)$ for the resulting element of $\mathrm {End}((\mathbf {C}^{n})^{\otimes k})$ according to the representation of $S_{k}$ on $(\mathbf {C}^{n})^{\otimes k}$. We also have fixed linear embeddings for $M\le n$

$$\begin{aligned} \mathbf {C}^{M}=\langle e_{1},\ldots ,e_{M}\rangle \subset \mathbf {C}^{n}=\langle e_{1},\ldots ,e_{n}\rangle ; \end{aligned}$$

similarly, ${\check{\mathbf {C}}}^{M}\subset {\check{\mathbf {C}}}^{n}$, hence using the type of isomorphism as in (2.1) for $M_{1},M_{2}\le k$ we obtain a fixed linear embedding

$$\begin{aligned} \mathrm {Hom}((\mathbf {C}^{n})^{\otimes M_{1}},(\mathbf {C}^{n})^{\otimes M_{2}})\subset \mathrm {End}((\mathbf {C}^{n})^{\otimes k}). \end{aligned}$$

The skew tableaux *s* yields a sequence of tableaux

$$\begin{aligned} \mu =\nu _{0}(s)\subset ^{1}\nu _{1}(s)\subset ^{1}\cdots \subset ^{1}\nu _{\mathfrak {D}}(s)=\uplambda \end{aligned}$$

where $\nu _{j}(s)$ is the YD given by $\mu$ along with the boxes of *s* containing numbers $\le m+j$. Let $B_{j}(s){\mathop {=}\limits ^{\mathrm {def}}}|\nu _{j}(s)|$ and similarly define $\nu _{j}(t),B_{j}(t)$ for $0\le j\le \mathfrak {D}$ using the boxes of *t*. We have

$$\begin{aligned} b=B_{0}(s)\le \cdots \le B_{\mathfrak {D}}(s)=B. \end{aligned}$$

Recall the projection operators $\mathfrak {p}_{\nu }$ from §§2.3. We use the natural inclusions, e.g., $S_{B_{i}(s)}\subset S_{B}$ to view, e.g., $\mathfrak {p}_{\nu _{i}(s)}\subset \mathbf {C}[S_{B}]$.

Let

$$\begin{aligned} A_{0}&{\mathop {=}\limits ^{\mathrm {def}}}\rho _{m}^{b}(\mathfrak {p}_{\mu })\in \mathrm {End}((\mathbf {C}^{m})^{\otimes b})\\&\cdots \\ A_{i}&{\mathop {=}\limits ^{\mathrm {def}}}\rho _{m+i}^{B_{i}(t)}(\mathfrak {p}_{\nu _{i}(t)})\left( A_{i-1}\otimes e_{m+i}^{\otimes \omega _{t}(m+i)}\otimes {\check{e}}_{m+i}^{\otimes \omega _{s}(m+i)}\right) \rho _{m+i}^{B_{i}(s)}(\mathfrak {p}_{\nu _{i}(s)})\quad i\ge 1\\ A_{i}&\in \mathrm {Hom}((\mathbf {C}^{m+i})^{\otimes B_{i}(s)},(\mathbf {C}^{m+i})^{\otimes B_{i}(t)})\\&\cdots \\ A_{s,t}^{\uplambda ,\mu }&{\mathop {=}\limits ^{\mathrm {def}}}A_{\mathfrak {D}}\in \mathrm {Hom}((\mathbf {C}^{n})^{\otimes B_{\mathfrak {D}}(s)},(\mathbf {C}^{n})^{\otimes B_{\mathfrak {D}}(t)})\cong \mathrm {End}((\mathbf {C}^{n})^{\otimes B}). \end{aligned}$$

We have the following proposition. Recalling the intertwiner $\mathcal {F}$ from Schur–Weyl duality (Proposition 2.4), we obtain a map

4.14 $$\begin{aligned} \mathcal {F}\otimes {\check{\mathcal {F}}}:\mathrm {End}((\mathbf {C}^{n})^{\otimes B})&\rightarrow \sum _{\begin{array}{c} \uplambda _{1},\uplambda _{2}\vdash B\\ \ell (\uplambda _{i})\le n \end{array} }W_{n}^{\uplambda _{1}}\otimes \check{W_{n}^{\uplambda _{2}}}\otimes V^{\uplambda _{1}}\otimes \check{V^{\uplambda _{2}}}\nonumber \\&\cong \sum _{\begin{array}{c} \uplambda _{1},\uplambda _{2}\vdash B\\ \ell (\uplambda _{i})\le n \end{array} }\mathrm {Hom}(W_{n}^{\uplambda _{2}},W_{n}^{\uplambda _{1}})\otimes \mathrm {Hom}(V^{\uplambda _{2}},V^{\uplambda _{1}}); \end{aligned}$$

this map is an intertwiner for $\mathsf {U}(n)\times \mathsf {U}(n)\times S_{B}\times S_{B}$. The second isomorphism is the canonical one explained in §§2.2.

#### Proposition 4.7

Under $\mathcal {F}\otimes {\check{\mathcal {F}}}$, $A_{s,t}^{\uplambda ,\mu }$ maps to

$$\begin{aligned} (\mathcal {F}\otimes {\check{\mathcal {F}}})[A_{s,t}^{\uplambda ,\mu }]=\mathcal {E}_{s,t}^{\uplambda ,\mu ,m}\otimes z_{s,t}^{\uplambda ,\mu }\in \mathrm {End}(W_{n}^{\uplambda })\otimes \mathrm {End}(V^{\uplambda }) \end{aligned}$$

for some $$z_{s,t}^{\uplambda ,\mu }\in \mathrm {End}(V^{\uplambda })$$.

#### Proof

The proof relies on the fact that up to scalar multiplication, $\mathcal {E}_{s,t}^{\uplambda ,\mu ,m}\in \mathrm {End}(W_{n}^{\uplambda })$ is uniquely characterized by the following properties:

- For $0\le i\le \mathfrak {D}$, $\mathcal {E}_{s,t}^{\uplambda ,\mu ,m}$ is in the $W_{m+i}^{\nu _{i}(t)}\otimes {\check{W}}_{m+i}^{\nu _{i}(s)}$-isotypic component of $\mathrm {End}(W^{\uplambda })$ for $\mathsf {U}(m+i)\times \mathsf {U}(m+i)$.
- $\mathcal {E}_{s,t}^{\uplambda ,\mu }$ commutes with $\mathsf {U}(m)$, or in other words, is invariant under the diagonal subgroup $\Delta _{\mathsf {U}(m)}\le \mathsf {U}(m)\times \mathsf {U}(m)$.

Consider the linear map by which $A_{i+1}$ is obtained from $A_{i}$ for $i\ge 0$, that is,

$$\begin{aligned} f_{i}(A_{i}){\mathop {=}\limits ^{\mathrm {def}}}\rho _{m+i+1}^{B_{i+1}(t)}(\mathfrak {p}_{\nu _{i+1}(t)})\left( A_{i}\otimes e_{m+i+1}^{\otimes \omega _{t}(m+i+1)}\otimes {\check{e}}_{m+i+1}^{\otimes \omega _{s}(m+i+1)}\right) \rho _{m+i+1}^{B_{i+1}(s)}(\mathfrak {p}_{\nu _{i+1}(s)}). \end{aligned}$$

This is a linear intertwiner for the group $\mathsf {U}(m+i)\times \mathsf {U}(m+i)\times S_{B_{i}(t)}\times S_{B_{i}(s)}$.

*Claim A. For all* $0\le i\le \mathfrak {D}$, $A_{s,t}^{\uplambda ,\mu }=A_{\mathfrak {D}}$ *is in the* $W_{m+i}^{\nu _{i}(t)}\otimes {\check{W}}_{m+i}^{\nu _{i}(s)}$-isotypic subspace of $\mathrm {End}((\mathbf {C}^{n})^{\otimes B})$ for $\mathsf {U}(m+i)\times \mathsf {U}(m+i)$.

*Proof of Claim A. *We prove by induction on *j* the following statement:

*S*(*i*, *j*) :  For $0\le i\le j\le \mathfrak {D}$, $A_{j}$ is in the $W_{n}^{\nu _{i}(t)}\otimes {\check{W}}_{n}^{\nu _{i}(s)}$-isotypic subspace of $\mathrm {Hom}((\mathbf {C}^{m+j})^{\otimes B_{j}(s)},(\mathbf {C}^{m+j})^{\otimes B_{j}(t)})$ for the group $\mathsf {U}(m+i)\times \mathsf {U}(m+i)$.

Consider the base cases $j=i$. By Schur–Weyl duality, and in particular (4.14), the fact that $A_{i}$ has the form

$$\begin{aligned} \rho _{m+i}^{B_{i}(t)}(\mathfrak {p}_{\nu _{i}(t)})Y_{i}\rho _{m+i}^{B_{i}(s)}(\mathfrak {p}_{\nu _{i}(s)}),\quad Y_{i}\in \mathrm {Hom}((\mathbf {C}^{m+i})^{\otimes B_{i}(s)},(\mathbf {C}^{m+i})^{\otimes B_{i}(t)}) \end{aligned}$$

implies that it is in the $V^{\nu _{i}(t)}\otimes {\check{V}}^{\nu _{i}(s)}$-isotypic component for $S_{B_{i}(t)}\times S_{B_{i}(s)}$, but this is the same as the $W_{n}^{\nu _{i}(t)}\otimes {\check{W}}_{n}^{\nu _{i}(s)}$-isotypic subspace for $\mathsf {U}(m+i)\times \mathsf {U}(m+i)$. For the inductive step, by the intertwining properties of $f_{i}$ stated above, if **S(***i* **,** *j***)** is true for some $i\le j,$ it is true for all $(i,j')$ with $j'\ge j$. Now taking $j=\mathfrak {D}$ proves Claim A. *The proof of Claim A is complete.*

*Claim B.* $A_{s,t}^{\uplambda ,\mu }$ *commutes with* $\mathsf {U}(m)$, *or in other words, is invariant under the diagonal subgroup* $\Delta _{\mathsf {U}(m)}\le \mathsf {U}(m)\times \mathsf {U}(m).$

*Proof of Claim B. *Using the intertwining properties of the maps $f_{i}$ again, we have for $u\in \mathsf {U}(m)$

$$\begin{aligned} \pi _{n}^{B}(u)A_{s,t}^{\uplambda ,\mu }\pi _{n}^{B}(u^{-1})&=\pi _{n}^{B}(u)f_{\mathfrak {D}-1}(f_{\mathfrak {D}-2}(\cdots (f_{0}(\rho _{m}^{b}(\mathfrak {p}_{\mu }))\cdots ))\pi _{n}^{B}(u^{-1})\\&=f_{\mathfrak {D}-1}(f_{\mathfrak {D}-2}(\cdots (f_{0}(\pi _{n}^{b}(u)\rho _{m}^{b}(\mathfrak {p}_{\mu })\pi _{n}^{b}(u^{-1}))\cdots ))\\&=f_{\mathfrak {D}-1}(f_{\mathfrak {D}-2}(\cdots (f_{0}(\rho _{m}^{b}(\mathfrak {p}_{\mu }))\cdots ))\\&=A_{s,t}^{\uplambda ,\mu }. \end{aligned}$$

*This completes the proof of Claim B.*

Now by Claim A with $i=\mathfrak {D}$ we have that, in reference to (4.14),

$$\begin{aligned} (\mathcal {F}\otimes {\check{\mathcal {F}}})[A_{s,t}^{\uplambda ,\mu }]\in \mathrm {End}(W_{n}^{\uplambda })\otimes \mathrm {End}(V^{\uplambda }), \end{aligned}$$

and the analogs of Claim A and Claim B hold for this element as $\mathcal {F}\otimes {\check{\mathcal {F}}}$ is a $\mathsf {U}(n)\times \mathsf {U}(n)$ intertwiner. Therefore, by the fact that $\mathcal {E}_{s,t}^{\uplambda ,\mu ,m}$ is uniquely characterized by **a **and **b**, we must have

$$\begin{aligned} (\mathcal {F}\otimes {\check{\mathcal {F}}})[A_{s,t}^{\uplambda ,\mu }]\in \mathbf {C}\mathcal {E}_{s,t}^{\uplambda ,\mu ,m}\otimes \mathrm {End}(V^{\uplambda }). \end{aligned}$$

This proves Proposition 4.7. $\quad \square$

### Normalization of $A_{s,t}^{\uplambda ,\mu }$

The goal of this section is to calculate $\Vert A_{s,t}^{\uplambda ,\mu }\Vert$, where the norm is the standard norm on $\mathrm {End}((\mathbf {C}^{n})^{\otimes B})$.

#### Proposition 4.8

We have

$$\begin{aligned} \Vert A_{s,t}^{\uplambda ,\mu }\Vert ^{2}=D_{\mu }(m)\frac{d_{\uplambda }^{2}(b!)^{2}}{d_{\mu }(B!)^{2}}\prod _{i=1}^{\mathfrak {D}}\omega _{t}(m+i)!\omega _{s}(m+i)!\ne 0. \end{aligned}$$

#### Proof

We do this calculation in stages. Firstly, the norm of $A_{0}$ coincides with the Hilbert–Schmidt norm on $\mathrm {End}((\mathbf {C}^{n})^{\otimes b})$ so is given by

$$\begin{aligned} \Vert A_{0}\Vert ^{2}=\mathrm {tr} (A_{0}A_{0}^{*})=\mathrm {tr} _{(\mathbf {C}^{m})^{\otimes b}}(\rho _{m}^{b}(\mathfrak {p}_{\mu })\rho _{m}^{b}(\mathfrak {p}_{\mu })^{*})=\mathrm {tr} _{(\mathbf {C}^{m})^{\otimes b}}(\rho _{m}^{b}(\mathfrak {p}_{\mu })^{2})=\mathrm {tr} _{(\mathbf {C}^{m})^{\otimes b}}(\rho _{m}^{b}(\mathfrak {p}_{\mu })) \end{aligned}$$

since $\rho _{m}^{b}(\mathfrak {p}_{\mu })$ is self-adjoint and idempotent. Using Schur–Weyl duality (Proposition 2.4), we obtain

$$\begin{aligned} \Vert A_{0}\Vert ^{2}=\mathrm {tr} _{(\mathbf {C}^{m})^{\otimes b}}(\rho _{m}(\mathfrak {p}_{\mu }))=D_{\mu }(m)d_{\mu }. \end{aligned}$$

We proceed to $A_{1}$. We have

4.15 $$\begin{aligned} \Vert A_{1}\Vert ^{2}&=\Vert \rho _{m+1}^{B_{1}(t)}(\mathfrak {p}_{\nu _{1}(t)})\left( A_{0}\otimes e_{m+1}^{\otimes \omega _{t}(m+1)}\otimes {\check{e}}_{m+1}^{\otimes \omega _{s}(m+1)}\right) \rho _{m+1}^{B_{1}(s)}(\mathfrak {p}_{\nu _{1}(s)})\Vert ^{2}\nonumber \\&=\langle \rho _{m+1}^{B_{1}(t)}(\mathfrak {p}_{\nu _{1}(t)})\left( A_{0}\otimes e_{m+1}^{\otimes \omega _{t}(m+1)}\otimes {\check{e}}_{m+1}^{\otimes \omega _{s}(m+1)}\right) \rho _{m+1}^{B_{1}(s)}(\mathfrak {p}_{\nu _{1}(s)}),\nonumber \\&\quad \left( A_{0}\otimes e_{m+1}^{\otimes \omega _{t}(m+1)}\otimes {\check{e}}_{m+1}^{\otimes \omega _{s}(m+1)}\right) \rangle \nonumber \\&=\frac{d_{\nu _{1}(t)}d_{\nu _{1}(s)}}{B_{1}(s)!B_{1}(t)!}\sum _{g_{t}\in S_{B_{1}(t)},g_{s}\in S_{B_{1}(s)}}\chi _{\nu _{1}(t)}(g_{t})\chi _{\nu _{1}(s)}(g_{s})\nonumber \\&\times \langle \rho _{m+1}^{B_{1}(t)}(g_{t})\left( A_{0}\otimes e_{m+1}^{\otimes \omega _{t}(m+1)}\otimes {\check{e}}_{m+1}^{\otimes \omega _{s}(m+1)}\right) \rho _{m+1}^{B_{1}(s)}(g_{s}),\nonumber \\&\left( A_{0}\otimes e_{m+1}^{\otimes \omega _{t}(m+1)}\otimes {\check{e}}_{m+1}^{\otimes \omega _{s}(m+1)}\right) \rangle \nonumber \\&=\frac{d_{\nu _{1}(t)}d_{\nu _{1}(s)}}{B_{1}(s)!B_{1}(t)!}\sum _{g_{t}\in S_{m}\times S_{\omega _{t}(m+1)},g_{s}\in S_{m}\times S_{\omega _{s}(m+1)}}\chi _{\nu _{1}(t)}(g_{t})\chi _{\nu _{1}(s)}(g_{s})\nonumber \\&\times \langle \rho _{m+1}^{B_{1}(t)}(g_{t})\left( A_{0}\otimes e_{m+1}^{\otimes \omega _{t}(m+1)}\otimes {\check{e}}_{m+1}^{\otimes \omega _{s}(m+1)}\right) \rho _{m+1}^{B_{1}(s)}(g_{s}),\nonumber \\&\left( A_{0}\otimes e_{m+1}^{\otimes \omega _{t}(m+1)}\otimes {\check{e}}_{m+1}^{\otimes \omega _{s}(m+1)}\right) \rangle \nonumber \\&=\frac{d_{\nu _{1}(t)}d_{\nu _{1}(s)}}{B_{1}(s)!B_{1}(t)!}\sum _{\begin{array}{c} g_{t}\in S_{b}\times S_{\omega _{t}(m+1)},g_{s}\in S_{b}\times S_{\omega _{s}(m+1)}\\ g_{t}=(g_{t}^{1},g_{t}^{2}),g_{s}=(g_{s}^{1},g_{s}^{2}) \end{array} }\chi _{\nu _{1}(t)}(g_{t})\chi _{\nu _{1}(s)}(g_{s})\nonumber \\&\langle \rho _{m}^{b}(g_{t}^{1})A_{0}\rho _{m}^{b}(g_{s}^{1}),A_{0}\rangle \end{aligned}$$

The second equality used that $\rho _{m+1}^{B_{1}(t)}(\mathfrak {p}_{\nu _{1}(t)}),\rho _{m+1}^{B_{1}(s)}(\mathfrak {p}_{\nu _{1}(s)})$ are self-adjoint projections and the fourth equality used that $A_{0}$ is a linear combination of basis elements involving only $e_{i},\check{e_{i}}$ with $i\le m$ and hence orthogonal to $e_{m+1}^{\otimes \omega _{t}(m+1)}\otimes {\check{e}}_{m+1}^{\otimes \omega _{s}(m+1)}$.

Now for each $g_{r}=(g_{r}^{1},g_{r}^{2})\in S_{b}\times S_{\omega _{r}(m+1)}$, $r\in \{s,t\}$ write

4.16 $$\begin{aligned} \chi _{\nu _{1}(r)}(g_{r})=\sum _{\tau _{1}(r)\vdash b,\tau _{2}(r)\vdash \omega _{r}(m+1)}\mathsf {LR}_{\tau _{1}(r),\tau _{2}(r)}^{\nu _{1}(r)}\chi _{\tau _{1}(r)}(g_{r}^{1})\chi _{\tau _{2}(r)}(g_{r}^{2}). \end{aligned}$$

Note that

$$\begin{aligned} \langle \rho _{m}^{b}(g_{t}^{1})A_{0}\rho _{m}^{b}(g_{s}^{1}),A_{0}\rangle \end{aligned}$$

is a matrix coefficient in $\mathrm {End}(V^{\mu })$ as a representation of $S_{b}\times S_{b}$. Thus, when we insert (4.16) into (4.15), the only terms that survive, by orthogonality of matrix coefficients and Schur orthogonality, have

$$\begin{aligned} \tau _{1}(t)=\tau _{1}(s)=\mu ,\quad \tau _{2}(t)=(\omega _{t}(m+1)),\tau _{2}(s)=(\omega _{s}(m+1)). \end{aligned}$$

By Pieri’s formula (Lemma 2.1) $\mathsf {LR}_{\mu ,(\omega _{r}(m+1))}^{\nu _{1}(r)}=1$ as $\mu \subset ^{1}\nu _{1}(r)$. So (4.15) together with these observations gives

$$\begin{aligned} \Vert A_{1}\Vert ^{2}&=\frac{d_{\nu _{1}(t)}d_{\nu _{1}(s)}}{B_{1}(t)!B_{1}(s)!}\sum _{\begin{array}{c} g_{t}\in S_{b}\times S_{\omega _{t}(m+1)},g_{s}\in S_{b}\times S_{\omega _{s}(m+1)}\\ g_{t}=(g_{t}^{1},g_{t}^{2}),g_{s}=(g_{s}^{1},g_{s}^{2}) \end{array} }\chi _{\mu }(g_{_{t}}^{1})\chi _{\mu }(g_{s}^{1})\langle \rho _{m}^{b}(g_{t}^{1})A_{0}\rho _{m}^{b}(g_{s}^{1}),A_{0}\rangle \\&=\frac{d_{\nu _{1}(t)}d_{\nu _{1}(s)}(b!)^{2}}{B_{1}(t)!B_{1}(s)!d_{\mu }^{2}}\omega _{t}(m+1)!\omega _{s}(m+1)!\langle \rho _{m}^{b}(\mathfrak {p}_{\mu })A_{0}\rho _{m}^{b}(\mathfrak {p}_{\mu }),A_{0}\rangle \\&=\frac{d_{\nu _{1}(t)}d_{\nu _{1}(s)}(b!)^{2}}{B_{1}(t)!B_{1}(s)!d_{\mu }^{2}}\omega _{t}(m+1)!\omega _{s}(m+1)!\Vert A_{0}\Vert ^{2}. \end{aligned}$$

Repeating these arguments, mutatis mutandis, gives

$$\begin{aligned} \Vert A_{2}\Vert ^{2}&=\frac{d_{\nu _{2}(t)}d_{\nu _{2}(s)}}{B_{2}(t)!B_{2}(s)!}\frac{\omega _{t}(m+2)!\omega _{s}(m+2)!B_{1}(t)!B_{1}(s)!}{d_{\nu _{1}(t)}d_{\nu _{1}(s)}}\Vert A_{1}\Vert \\&=\frac{d_{\nu _{2}(t)}d_{\nu _{2}(s)}(b!)^{2}}{d_{\mu }^{2}B_{2}(t)!B_{2}(s)!}\omega _{t}(m+2)!\omega _{s}(m+2)!\omega _{t}(m+1)!\omega _{s}(m+1)!\Vert A_{0}\Vert ^{2}. \end{aligned}$$

Continuing to iterate this up to $A_{\mathfrak {D}}=A_{s,t}^{\uplambda ,\mu }$ gives the required

$$\begin{aligned} \Vert A_{s,t}^{\uplambda ,\mu }\Vert ^{2}&=\Vert A_{0}\Vert ^{2}\frac{d_{\uplambda }^{2}(b!)^{2}}{d_{\mu }^{2}(B!)^{2}}\prod _{i=1}^{\mathfrak {D}}\omega _{t}(m+i)!\omega _{s}(m+i)!\\&=D_{\mu }(m)\frac{d_{\uplambda }^{2}(b!)^{2}}{d_{\mu }(B!)^{2}}\prod _{i=1}^{\mathfrak {D}}\omega _{t}(m+i)!\omega _{s}(m+i)!. \end{aligned}$$

$$\square $$

### Projection of $A_{s,t}^{\uplambda ,\mu }\otimes X$ to invariant vectors

We now calculate ${\hat{P}}[A_{s,t}^{\uplambda ,\mu }\otimes X]$ where ${\hat{P}}$ is the projection onto the $\mathsf {U}(n)$-invariant vectors in $\mathrm {End}((\mathbf {C}^{n})^{\otimes B})\otimes \mathrm {End}((\mathbf {C}^{n})^{\otimes p})$, and

$$\begin{aligned} X=e_{i_{1}}\otimes \cdots \otimes e_{i_{p}}\otimes {\check{e}}_{j_{1}}\otimes \cdots \otimes {\check{e}}_{j_{p}}\in \mathrm {End}(\left( \mathbf {C}^{n}\right) ^{\otimes p}) \end{aligned}$$

with

$$\begin{aligned} i_{r},j_{r}\in [m+1,n],\quad r\in [p]. \end{aligned}$$

We identify $\mathrm {End}((\mathbf {C}^{n})^{\otimes B})\otimes \mathrm {End}((\mathbf {C}^{n})^{\otimes p})\cong \mathrm {End}((\mathbf {C}^{n})^{\otimes (B+p)})$ via canonical isomorphisms as in (2.1) and the map

4.17 $$\begin{aligned}&e_{i_{1}}\otimes \cdots \otimes e_{i_{B}}\otimes {\check{e}}_{j_{1}}\otimes \cdots \otimes {\check{e}}_{j_{B}}\otimes e_{i_{B+1}}\otimes \cdots \otimes e_{i_{B+p}}\otimes {\check{e}}_{j_{B+1}}\otimes \cdots \otimes {\check{e}}_{j_{B+p}}\nonumber \\ \mapsto \,&e_{i_{1}}\otimes \cdots \otimes e_{i_{B}}\otimes e_{i_{B+1}}\otimes \cdots \otimes e_{i_{B+p}}\otimes {\check{e}}_{j_{1}}\otimes \cdots \otimes {\check{e}}_{j_{B}}\otimes {\check{e}}_{j_{B+1}}\otimes \cdots \otimes {\check{e}}_{j_{B+p}}. \end{aligned}$$

Using this identification, we view $\mathrm {End}((\mathbf {C}^{n})^{\otimes B})\otimes \mathrm {End}((\mathbf {C}^{n})^{\otimes p})$ as a unitary representation of $\mathsf {U}(n)\times S_{B+p}\times \mathsf {U}(n)\times S_{B+p}$; the map ${\hat{P}}$ is a $\mathbf {C}[S_{B+p}]$-bimodule morphism. Hence,

$$\begin{aligned} {\hat{P}}[A_{s,t}^{\uplambda ,\mu }\otimes X]&={\hat{P}}[\rho _{n}^{B}(\mathfrak {p}_{\nu _{\mathfrak {D}}(t)})\left( A_{\mathfrak {D}-1}\otimes e_{m+\mathfrak {D}}^{\otimes \omega _{t}(m+\mathfrak {D})}\otimes {\check{e}}_{m+\mathfrak {D}}^{\otimes \omega _{s}(m+\mathfrak {D})}\right) \rho _{n}^{B}(\mathfrak {p}_{\nu _{\mathfrak {D}}(s)})\otimes X]\\&=\rho _{n}^{B}(\mathfrak {p}_{\nu _{\mathfrak {D}}(t)}){\hat{P}}[\left( A_{\mathfrak {D}-1}\otimes e_{m+\mathfrak {D}}^{\otimes \omega _{t}(m+\mathfrak {D})}\otimes {\check{e}}_{m+\mathfrak {D}}^{\otimes \omega _{s}(m+\mathfrak {D})}\right) \otimes X]\rho _{n}^{B}(\mathfrak {p}_{\nu _{\mathfrak {D}}(s)})\\&=\rho _{n}^{B}(\mathfrak {p}_{\nu _{\mathfrak {D}}(t)}){\hat{P}}[A_{\mathfrak {D}-1}\otimes X_{1}]\rho _{n}^{B}(\mathfrak {p}_{\nu _{\mathfrak {D}}(s)}) \end{aligned}$$

where $X_{1}{\mathop {=}\limits ^{\mathrm {def}}}e_{m+\mathfrak {D}}^{\otimes \omega _{t}(m+\mathfrak {D})}\otimes {\check{e}}_{m+\mathfrak {D}}^{\otimes \omega _{s}(m+\mathfrak {D})}\otimes X$. Repeating this argument gives

4.18 $$\begin{aligned} {\hat{P}}[A_{s,t}^{\uplambda ,\mu }\otimes X]&=\rho _{n}^{B}(\mathfrak {p}_{\nu _{\mathfrak {D}}(t)}){\hat{P}}[A_{\mathfrak {D}-1}\otimes X_{1}]\rho _{n}(\mathfrak {p}_{\nu _{\mathfrak {D}}(s)})\nonumber \\&=\rho _{n}^{B}(\mathfrak {p}_{\nu _{\mathfrak {D}}(t)}\mathfrak {p}_{\nu _{\mathfrak {D}-1}(t)}){\hat{P}}[A_{\mathfrak {D}-2}\otimes X_{2}]\rho _{n}^{B}(\mathfrak {p}_{\nu _{\mathfrak {D}-1}(s)}\mathfrak {p}_{\nu _{\mathfrak {D}}(s)})\nonumber \\&=\cdots \end{aligned}$$

4.19 $$\begin{aligned}&=\rho _{n}^{B}(\mathfrak {p}_{\nu _{\mathfrak {D}}(t)}\cdots \mathfrak {p}_{\nu _{1}(t)}){\hat{P}}[A_{0}\otimes X_{\mathfrak {D}}]\rho _{n}^{B}(\mathfrak {p}_{\nu _{1}(s)}\cdots \mathfrak {p}_{\nu _{\mathfrak {D}}(s)})\nonumber \\&=\rho _{n}^{B}(\mathfrak {p}_{\nu _{\mathfrak {D}}(t)}\cdots \mathfrak {p}_{\nu _{1}(t)}){\hat{P}}[\rho _{m}^{b}(\mathfrak {p}_{\mu })\otimes X_{\mathfrak {D}}]\rho _{n}^{B}(\mathfrak {p}_{\nu _{1}(s)}\cdots \mathfrak {p}_{\nu _{\mathfrak {D}}(s)}) \end{aligned}$$

where

$$\begin{aligned} X_{\mathfrak {D}}{\mathop {=}\limits ^{\mathrm {def}}}e_{m+1}^{\otimes \omega _{t}(m+1)}\otimes {\check{e}}_{m+1}^{\otimes \omega _{s}(m+1)}\otimes \cdots \otimes e_{m+\mathfrak {D}}^{\otimes \omega _{t}(m+\mathfrak {D})}\otimes {\check{e}}_{m+\mathfrak {D}}^{\otimes \omega _{s}(m+\mathfrak {D})}\otimes X. \end{aligned}$$

So it remains to calculate ${\hat{P}}[\rho _{m}^{b}(\mathfrak {p}_{\mu })\otimes X_{\mathfrak {D}}]$.

The Weingarten calculus (Proposition 2.6) yields

4.20 $$\begin{aligned} {\hat{P}}[\rho _{m}^{b}(\mathfrak {p}_{\mu })\otimes X_{\mathfrak {D}}]=\rho _{n}^{B+p}(\Phi [\rho _{m}^{b}(\mathfrak {p}_{\mu })\otimes X_{\mathfrak {D}}]\mathrm {Wg}_{n,B+p}) \end{aligned}$$

where

$$\begin{aligned} \Phi [\rho _{m}^{b}(\mathfrak {p}_{\mu })\otimes X_{\mathfrak {D}}]=\sum _{\sigma \in S_{B+p}}\mathrm {tr} [\rho _{n}^{B+p}(\sigma )^{-1}(\rho _{m}(\mathfrak {p}_{\mu })\otimes X'_{\mathfrak {D}})]\sigma \end{aligned}$$

and $X'_{\mathfrak {D}}\in \mathrm {End}(\langle e_{m+1},\ldots ,e_{n}\rangle ^{\otimes B-b+p})$ is the element corresponding to $X_{\mathfrak {D}}$ under the fixed isomorphism

$$\begin{aligned}&\left( \mathbf {C}^{n}\right) ^{\otimes \omega _{t}(m+1)}\otimes \check{\left( \mathbf {C}^{n}\right) }^{\otimes \omega _{s}(m+1)}\otimes \cdots \otimes \left( \mathbf {C}^{n}\right) {}^{\otimes \omega _{t}(m+\mathfrak {D})}\\&\qquad \otimes \check{\left( \mathbf {C}^{n}\right) }^{\otimes \omega _{s}(m+\mathfrak {D})}\otimes \mathrm {End}(\left( \mathbf {C}^{n}\right) ^{\otimes p})\\&\quad \cong \mathrm {End}(\left( \mathbf {C}^{n}\right) ^{\otimes B-b+p}) \end{aligned}$$

that preserves the order of the factors in both

$$\begin{aligned}&\left( \mathbf {C}^{n}\right) ^{\otimes \omega _{t}(m+1)}\otimes \cdots \otimes \left( \mathbf {C}^{n}\right) {}^{\otimes \omega _{t}(m+\mathfrak {D})}\otimes (\mathbf {C}^{n})^{\otimes p},\,\check{\left( \mathbf {C}^{n}\right) }^{\otimes \omega _{s}(m+1)}\\&\quad \otimes \cdots \otimes \check{\left( \mathbf {C}^{n}\right) }^{\otimes \omega _{s}(m+\mathfrak {D})}\otimes \left( \check{\mathbf {C}^{n}}\right) {}^{\otimes p}, \end{aligned}$$

so that

4.21 $$\begin{aligned}&X'_{\mathfrak {D}}{\mathop {=}\limits ^{\mathrm {def}}}e_{m+1}^{\otimes \omega _{t}(m+1)}\otimes \cdots \otimes e_{m+\mathfrak {D}}^{\otimes \omega _{t}(m+\mathfrak {D})}\otimes e_{i_{1}}\otimes \cdots \otimes e_{i_{p}}\otimes {\check{e}}_{m+1}^{\otimes \omega _{s}(m+1)}\otimes \cdots \nonumber \\&\quad \otimes {\check{e}}_{m+\mathfrak {D}}^{\otimes \omega _{s}(m+\mathfrak {D})}\otimes {\check{e}}_{j_{1}}\otimes \cdots \otimes {\check{e}}_{j_{p}}. \end{aligned}$$

We make an observation that if $q_{r}$ denotes the number of $\ell \in [p]$ such that $j_{\ell }=m+r$, and $p_{r}$ denotes the number of $\ell \in [p]$ such that $i_{\ell }=m+r$, if we do **not** have

4.22 $$\begin{aligned}&(\omega _{s}(m+1)+q_{1},\omega _{s}(m+2)+q_{2},\ldots ,\omega _{s}(m+\mathfrak {D})+q_{\mathfrak {D}}) \nonumber \\ =&(\omega _{t}(m+1)+p_{1},\omega _{t}(m+2)+p_{2},\ldots ,\omega _{t}(m+\mathfrak {D})+p_{\mathfrak {D}}) \end{aligned}$$

then $\mathrm {tr} [\rho _{n}^{B+p}(\sigma )^{-1}(\rho _{m}^{b}(\mathfrak {p}_{\mu })\otimes X'_{\mathfrak {D}})]=0$ for all $\sigma \in S_{B+p}$; hence, $\Phi [\rho _{m}^{b}(\mathfrak {p}_{\mu })\otimes X_{\mathfrak {D}}]=0$, ${\hat{P}}[\rho _{m}^{b}(\mathfrak {p}_{\mu })\otimes X_{\mathfrak {D}}]=0$, and ${\hat{P}}[A_{s,t}^{\uplambda ,\mu }\otimes X]=0$.

*So now assume* (4.22) *holds.*

To calculate $\Phi [\rho _{m}^{b}(\mathfrak {p}_{\mu })\otimes X_{\mathfrak {D}}]$, we note that since $X'_{\mathfrak {D}}$ is a tensor of vectors orthogonal to $\mathbf {C}^{m}$,

$$\begin{aligned} \mathrm {tr} [\rho _{n}^{B+p}(\sigma )^{-1}(\rho _{m}^{b}(\mathfrak {p}_{\mu })\otimes X'_{\mathfrak {D}})]=0 \end{aligned}$$

unless $\sigma =(\sigma _{1},\sigma _{2})\in S_{b}\times S_{B-b+p}$, and in this case, by Schur–Weyl duality (Proposition 2.4)

$$\begin{aligned} \mathrm {tr} [\rho _{n}^{B+p}(\sigma )^{-1}(\rho _{m}^{b}(\mathfrak {p}_{\mu })\otimes X'_{\mathfrak {D}})]&=\mathrm {tr} [\rho _{m}^{b}(\sigma _{1}^{-1}\mathfrak {p}_{\mu })\otimes (\rho _{n}^{B-b+p}(\sigma _{2}^{-1})X'_{\mathfrak {D}})]\\&=\mathrm {tr} [\rho _{n}^{B-b+p}(\sigma _{2}^{-1})X'_{\mathfrak {D}})]\mathrm {tr} [\rho _{m}^{b}(\sigma _{1}^{-1}\mathfrak {p}_{\mu })]\\&=\mathrm {tr} [\rho _{n}^{B-b+p}(\sigma _{2}^{-1})X'_{\mathfrak {D}})]\sum _{\begin{array}{c} \mu '\vdash b\\ \ell (\mu ')\le m \end{array} }D_{\mu '}(m)\chi _{\mu '}(\sigma _{1}^{-1}\mathfrak {p}_{\mu })\\&=\mathrm {tr} [\rho _{n}^{B-b+p}(\sigma _{2}^{-1})X'_{\mathfrak {D}})]D_{\mu }(m)\chi _{\mu }(\sigma _{1}^{-1}). \end{aligned}$$

Therefore,

4.23 $$\begin{aligned} \Phi [\rho _{m}^{b}(\mathfrak {p}_{\mu })\otimes X_{\mathfrak {D}}]&=\sum _{(\sigma _{1},\sigma _{2})\in S_{b}\times S_{B-b+p}}\mathrm {tr} [\rho _{n}^{B+p}(\sigma )^{-1}(\rho _{m}^{b}(\mathfrak {p}_{\mu })\otimes X'_{\mathfrak {D}})]\sigma \nonumber \\&=D_{\mu }(m)\sum _{(\sigma _{1},\sigma _{2})\in S_{b}\times S_{B-b+p}}\mathrm {tr} [\rho _{n}^{B-b+p}(\sigma _{2}^{-1})X'_{\mathfrak {D}}]\chi _{\mu }(\sigma _{1}^{-1})(\sigma _{1},\sigma _{2}). \end{aligned}$$

Here we write $(\sigma _{1},\sigma _{2})$ for the element of $S_{B+p}$ corresponding to $(\sigma _{1},\sigma _{2})\in S_{b}\times S_{B-b+p}$.

Given (4.21), the value of $\mathrm {tr} [\rho _{n}^{B-b+p}(\sigma _{2}^{-1})X'_{\mathfrak {D}}]$ is either 1 or 0. The values of $\sigma _{2}$ for which $\mathrm {tr} [\rho _{n}^{B-b+p}(\sigma _{2}^{-1})X'_{\mathfrak {D}}]=1$ are a right coset

$$\begin{aligned} \pi _{0}S_{\Pi } \end{aligned}$$

where $\pi _{0}\in S_{B-b+p}$ and $S_{\Pi }\le S_{B-b+p}$ is the subgroup of elements respecting a certain partition $\Pi$ of $[B-b+p]$ dictated by the indices of $X'_{\mathfrak {D}}$. The blocks of $\Pi$ have sizes given by either side of (4.22). Note $\sum _{i=1}^{\mathfrak {D}}p_{i}=\sum _{i=1}^{\mathfrak {D}}q_{i}=p$. Moreover, we have

$$\begin{aligned} S_{s}{\mathop {=}\limits ^{\mathrm {def}}}S_{\omega _{s}(m+1)}\times S_{\omega _{s}(m+2)}\times \cdots \times S_{\omega _{s}(m+\mathfrak {D}-1)}\times S_{\omega _{s}(n)}\le S_{\Pi }. \end{aligned}$$

From (4.23), we now have

4.24 $$\begin{aligned} \Phi [\rho _{m}^{b}(\mathfrak {p}_{\mu })\otimes X_{\mathfrak {D}}]&=D_{\mu }(m)\sum _{(\sigma _{1},\sigma _{2})\in S_{b}\times S_{B-b+p}}\mathrm {tr} [\rho _{n}^{B-b+p}(\sigma _{2}^{-1})X'_{\mathfrak {D}}]\chi _{\mu }(\sigma _{1}^{-1})(\sigma _{1},\sigma _{2})\nonumber \\&=D_{\mu }(m)\sum _{(\sigma _{1},\sigma _{2})\in S_{b}\times S_{\Pi }}\chi _{\mu }(\sigma _{1}^{-1})(\sigma _{1},\pi _{0}\sigma _{2})\nonumber \\&=D_{\mu }(m)\sum _{[\tau ]\in S_{\Pi }/S_{s}}\sum _{(\sigma _{1},\sigma _{2})\in S_{b}\times S_{s}}\chi _{\mu }(\sigma _{1}^{-1})(\sigma _{1},\pi _{0}\tau \sigma _{2})\nonumber \\&=D_{\mu }(m)\sum _{[\tau ]\in S_{\Pi }/S_{s}}(\mathrm {id}_{S_{b}},\pi _{0}\tau )\sum _{(\sigma _{1},\sigma _{2})\in S_{b}\times S_{s}}\chi _{\mu }(\sigma _{1}^{-1})(\sigma _{1},\sigma _{2})\nonumber \\&=\frac{b!}{d_{\mu }}|S_{s}|D_{\mu }(m)\sum _{[\tau ]\in S_{\Pi }/S_{s}}(\mathrm {id}_{S_{b}},\pi _{0}\tau )\mathfrak {q}_{s} \end{aligned}$$

where

$$\begin{aligned} \mathfrak {q}_{s}{\mathop {=}\limits ^{\mathrm {def}}}\frac{d_{\mu }}{b!}\frac{1}{|S_{s}|}\sum _{(\sigma _{1},\sigma _{2})\in S_{b}\times S_{s}}\chi _{\mu }(\sigma _{1}^{-1})(\sigma _{1},\sigma _{2})\in \mathbf {C}[S_{B}]\subset \mathbf {C}[S_{B+p}] \end{aligned}$$

is an element which in any unitary representation of $S_{B+p}$ gives the orthogonal projection onto the $V^{\mu }\otimes \mathrm {triv}_{S_{s}}$-isotypic subspace for $S_{b}\times S_{s}$.

Combining (4.20) and (4.24) and recalling the definition of $\mathrm {Wg}_{n,B+p}$ from (2.12) give

$$\begin{aligned} {\hat{P}}[\rho _{m}^{b}(\mathfrak {p}_{\mu })\otimes X_{\mathfrak {D}}]&=\rho _{n}^{B+p}(\Phi [\rho _{m}^{b}(\mathfrak {p}_{\mu })\otimes X_{\mathfrak {D}}]\mathrm {Wg}_{n,B+p})\\&=\frac{b!}{d_{\mu }}|S_{s}|D_{\mu }(m)\sum _{[\tau ]\in S_{\Pi }/S_{s}}\rho _{n}^{B+p}(\mathrm {id}_{S_{b}},\pi _{0}\tau )\rho _{n}^{B+p}(\mathfrak {q}_{s})\rho _{n}^{B+p}(\mathrm {Wg}_{n,B+p})\\&=\frac{b!}{d_{\mu }}\frac{|S_{s}|}{(B+p)!}D_{\mu }(m)\sum _{\begin{array}{c} \uplambda '\vdash B+p\\ \ell (\uplambda ')\le n \end{array} }\frac{d_{\uplambda '}}{D_{\uplambda '}(n)}\\&\quad \sum _{[\tau ]\in S_{\Pi }/S_{s}}\rho _{n}^{B+p}(\mathrm {id}_{S_{b}},\pi _{0}\tau )\rho _{n}(\mathfrak {q}_{s}\mathfrak {p}_{\uplambda '}). \end{aligned}$$

Combined with (4.19) this gives

4.25 $$\begin{aligned}&{\hat{P}}[A_{s,t}^{\uplambda ,\mu }\otimes X] =\frac{b!}{d_{\mu }}\frac{|S_{s}|}{(B+p)!}D_{\mu }(m)\nonumber \\&\qquad \sum _{\begin{array}{c} \uplambda '\vdash B+p\\ \ell (\uplambda ')\le n \end{array} }\frac{d_{\uplambda '}}{D_{\uplambda '}(n)}\rho _{n}^{B+p}\left( \mathfrak {p}_{\nu _{\mathfrak {D}}(t)}\cdots \mathfrak {p}_{\nu _{1}(t)}\sum _{[\tau ]\in S_{\Pi }/S_{s}}(\mathrm {id}_{S_{b}},\pi _{0}\tau )\mathfrak {q}_{s}\mathfrak {p}_{\uplambda '}\mathfrak {p}_{\nu _{1}(s)}\cdots \mathfrak {p}_{\nu _{\mathfrak {D}}(s)}\right) \end{aligned}$$

that is the main result of this section.

### The norm of the projection of $A_{s,t}^{\uplambda ,\mu }\otimes X$

We now turn to the calculation of $\Vert {\hat{P}}[A_{s,t}^{\uplambda ,\mu }\otimes X]\Vert$ where the norm is the natural one induced by the standard inner product on $\mathbf {C}^{n}$. We identify $\mathrm {End}((\mathbf {C}^{n})^{\otimes B+p})\cong \mathrm {End}((\mathbf {C}^{n})^{\otimes B})\otimes \mathrm {End}(\left( \mathbf {C}^{n}\right) ^{\otimes p})$ using the isomorphism (4.17); after this identification the norm on $\mathrm {End}((\mathbf {C}^{n})^{\otimes B})\otimes \mathrm {End}(\left( \mathbf {C}^{n}\right) ^{\otimes p})$ coincides with the Hilbert–Schmidt norm on $\mathrm {End}((\mathbf {C}^{n})^{\otimes B+p})$. The main result of this section is:

#### Proposition 4.9

If (4.22) holds, we have

$$\begin{aligned} \Vert {\hat{P}}[A_{s,t}^{\uplambda ,\mu }\otimes X]\Vert ^{2}&\le \frac{(b!)^{2}|S_{\Pi }|^{2}D_{\mu }(m)^{2}}{d_{\mu }((B+p)!)^{2}}\sum _{\begin{array}{c} \uplambda '\vdash B+p\\ \ell (\uplambda ')\le n \end{array} }\frac{d_{\uplambda '}^{2}d_{\uplambda '/\uplambda }}{D_{\uplambda '}(n)}. \end{aligned}$$

Otherwise, $$\Vert {\hat{P}}[A_{s,t}^{\uplambda ,\mu }\otimes X]\Vert =0$$.

We view $(\mathbf {C}^{n})^{\otimes B+p}$ with its given inner product as a unitary representation of $\mathsf {U}(n)\times S_{B+p}$ in the usual way involved in Schur–Weyl duality. For $\uplambda '\vdash m+p$ with $\ell (\uplambda ')\le n$, we let $Z^{\uplambda '}$ denote the $W_{n}^{\uplambda '}\times V^{\uplambda '}$-isotypic subspace of $(\mathbf {C}^{n})^{\otimes B+p}$ for $\mathsf {U}(n)\times S_{B+p}$. By Schur–Weyl duality (Proposition 2.4), this space is itself an irreducible representation of $\mathsf {U}(n)\times S_{B+p}$ isomorphic to $W_{n}^{\uplambda '}\times V^{\uplambda '}$. Since ${\hat{P}}[A_{s,t}^{\uplambda ,\mu }\otimes X]\in \rho _{n}^{B+p}(\mathbf {C}[S_{B+p}])$, it preserves each $Z^{\uplambda '}$. Therefore, we have

4.26 $$\begin{aligned} \Vert {\hat{P}}[A_{s,t}^{\uplambda ,\mu }\otimes X]\Vert ^{2}=\sum _{\begin{array}{c} \uplambda '\vdash B+p \\ \ell (\uplambda ')\le n \end{array} }\Vert {\hat{P}}[A_{s,t}^{\uplambda ,\mu }\otimes X]\Vert _{\uplambda '}^{2} \end{aligned}$$

where $\Vert {\hat{P}}[A_{s,t}^{\uplambda ,\mu }\otimes X]\Vert _{\uplambda '}$ is the Hilbert–Schmidt norm of ${\hat{P}}[A_{s,t}^{\uplambda ,\mu }\otimes X]$ acting on $Z^{\uplambda '}$.

We may assume (4.22) holds, otherwise ${\hat{P}}[A_{s,t}^{\uplambda ,\mu }\otimes X]=0$ and Proposition 4.9 is proved. In this case, using that $\mathfrak {p}_{\uplambda '}$ is central in $\mathbf {C}[S_{B+p}]$, inspection of (4.25) gives that

4.27 $$\begin{aligned}&\Vert {\hat{P}}[A_{s,t}^{\uplambda ,\mu }\otimes X]\Vert _{\uplambda '}=\frac{b!}{d_{\mu }}\frac{|S_{s}|}{(B+p)!}D_{\mu }(m)\frac{d_{\uplambda '}}{\sqrt{D_{\uplambda '}(n)}}\Vert \mathfrak {p}_{\nu _{\mathfrak {D}}(t)}\cdots \mathfrak {p}_{\nu _{1}(t)}\nonumber \\&\quad \sum _{[\tau ]\in S_{\Pi }/S_{s}}(\mathrm {id}_{S_{b}},\pi _{0}\tau )\mathfrak {q}_{s}\mathfrak {p}_{\nu _{1}(s)}\cdots \mathfrak {p}_{\nu _{\mathfrak {D}}(s)}\Vert _{V^{\uplambda '}} \end{aligned}$$

where for $z\in \mathbf {C}[S_{B+p}]$ we write $\Vert z\Vert _{V^{\uplambda '}}$ for the Hilbert–Schmidt norm of *z* acting on $V^{\uplambda '}$. Notice that we obtained a factor $\sqrt{D_{\uplambda '}(n)}$ from the multiplicity of $V^{\uplambda '}$ in $Z^{\uplambda '}$. Due to the presence of $\mathfrak {p}_{\nu _{\mathfrak {D}}(s)}=\mathfrak {p}_{\uplambda }$ in (4.27), the right-hand side of (4.27) is zero unless $\uplambda \subset _{p}\uplambda '$, and hence,

4.28 $$\begin{aligned} \Vert {\hat{P}}[A_{s,t}^{\uplambda ,\mu }\otimes X]\Vert ^{2}=\sum _{\begin{array}{c} \uplambda \subset _{p}\uplambda '\\ \ell (\uplambda ')\le n \end{array} }\Vert {\hat{P}}[A_{s,t}^{\uplambda ,\mu }\otimes X]\Vert _{\uplambda '}^{2}. \end{aligned}$$

To calculate the Hilbert–Schmidt norm in (4.27), we study in detail the operator

$$\begin{aligned} Q{\mathop {=}\limits ^{\mathrm {def}}}\mathfrak {q}_{s}\mathfrak {p}_{\nu _{1}(s)}\cdots \mathfrak {p}_{\nu _{\mathfrak {D}}(s)}. \end{aligned}$$

Let $\pi _{\uplambda '}$ denote the representation of $S_{B+p}$ on $V^{\uplambda '}$.

#### Lemma 4.10

With notation as above, $\pi _{\uplambda '}(Q)$ is a self-adjoint idempotent element of $\mathrm {End}(V^{\uplambda '})$. Similarly, $\pi _{\uplambda '}(\mathfrak {p}_{\nu _{1}(t)}\cdots \mathfrak {p}_{\nu _{\mathfrak {D}}(t)})$ is a self-adjoint idempotent.

#### Proof

Recall that $\mathfrak {q}_{s}$ is the orthogonal projection onto the $V^{\mu }\otimes \mathrm {triv}_{S_{s}}$-isotypic subspace for

$$\begin{aligned} S_{b}\times S_{s}&=S_{b}\times S_{\omega _{s}(m+1)}\times S_{\omega _{s}(m+2)}\times \cdots \times S_{\omega _{s}(m+\mathfrak {D}-1)}\times S_{\omega _{s}(n)}\le S_{B}\le S_{B+p}. \end{aligned}$$

One see that this commutes with all $\mathfrak {p}_{\nu _{1}(s)},\ldots ,\mathfrak {p}_{\nu _{\mathfrak {D}}(s)}$ in $\mathbf {C}[S_{B+p}]$, because for any $i\in [\mathfrak {D}]$, we can write

$$\begin{aligned} \mathfrak {q}_{s}=Q_{1}Q_{2} \end{aligned}$$

where $Q_{1},Q_{2}$ are commuting projections supported, respectively, on the two factors of $S_{B}=S_{B_{i}(s)}\times S_{B-B_{i}(s)}$. But $\mathfrak {p}_{\nu _{i}(s)}$ commutes with all elements of $S_{B}$ in the $S_{B-B_{i}(s)}$ factor, so commutes with $Q_{2}$. It also commutes with $Q_{1}$ since $\mathfrak {p}_{\nu _{i}(s)}$ is a central element of $\mathbf {C}[S_{B_{i}(s)}]$. The $\mathfrak {p}_{\nu _{1}(s)},\ldots ,\mathfrak {p}_{\nu _{\mathfrak {D}}(s)}$ are easily seen to commute with each other: $\mathfrak {p}_{\nu _{i}(s)}$ is central in $\mathbf {C}[S_{B_{i}(s)}]$ and hence commutes with all elements $\mathfrak {p}_{\nu _{j}(s)}$ with $j\le i$. Thus, $\pi _{\uplambda '}(Q)$ is a product of commuting self-adjoint idempotents and so is itself a self-adjoint idempotent. The same arguments show that $\pi _{\uplambda '}(\mathfrak {p}_{\nu _{1}(t)}\cdots \mathfrak {p}_{\nu _{\mathfrak {D}}(t)})$ is a self-adjoint idempotent. $\quad \square$

We now calculate the dimension of $\pi _{\uplambda '}(Q)V^{\uplambda '}$. This is the eigenspace of $\pi _{\uplambda '}(Q)$ with eigenvalue 1 and also the orthogonal complement to the kernel of $\pi _{\uplambda '}(Q)$.

#### Lemma 4.11

With notation as above, if $\uplambda \subset _{p}\uplambda '\vdash B+p$, $\dim \left( \pi _{\uplambda '}(Q)V^{\uplambda '}\right) =d_{\uplambda '/\uplambda }d_{\mu }.$

#### Proof

Firstly, as $Q\in \mathbf {C}[S_{B}]$ and contains the factor

$$\begin{aligned} \mathfrak {p}_{\nu _{\mathfrak {D}}(s)}=\mathfrak {p}_{\uplambda }, \end{aligned}$$

we have

4.29 $$\begin{aligned} \dim \left( \pi _{\uplambda '}(Q)V^{\uplambda '}\right)= & {} \dim \mathrm {Hom}_{\mathbf {C}[S_{B}]}(V^{\uplambda },V^{\uplambda '})\dim \left( \pi _{\uplambda }(Q)V^{\uplambda }\right) \nonumber \\= & {} d_{\uplambda '/\uplambda }\dim \left( \pi _{\uplambda }(Q)V^{\uplambda }\right) . \end{aligned}$$

Given this, we now consider *Q* acting on $V^{\uplambda }$. This is the same as

$$\begin{aligned} \pi _{\uplambda }(Q_{1}),\quad Q_{1}{\mathop {=}\limits ^{\mathrm {def}}}\mathfrak {q}_{s}\mathfrak {p}_{\nu _{1}(s)}\cdots \mathfrak {p}_{\nu _{\mathfrak {D}-1}(s)}. \end{aligned}$$

We have $\pi _{\uplambda }(Q_{1})$ is a self-adjoint idempotent by the same proof as that of Lemma 4.10. Then $Q_{1}$ is supported on, and preserves, the $V^{\nu _{\mathfrak {D}-1}(s)}\otimes \mathrm {triv}$-isotypic component for $S_{B_{\mathfrak {D}-1}}\times S_{\omega _{s}(n)}\le S_{B}$. By Pieri’s formula (Lemma 2.1), as $\nu _{\mathfrak {D}-1}\subset ^{1}\nu _{\mathfrak {D}}=\uplambda$, this isotypic component consists of a unique isomorphic copy of $V^{\nu _{\mathfrak {D}-1}(s)}\otimes \mathrm {triv}$ in $V^{\uplambda }$. Moreover, when identifying this space with $V^{\nu _{\mathfrak {D}-1}(s)}$, the action of $\pi _{\uplambda }(Q_{1})$ is given by

$$\begin{aligned} \pi _{\nu _{\mathfrak {D}-1}(s)}(Q_{2}),\quad Q_{2}{\mathop {=}\limits ^{\mathrm {def}}}\mathfrak {q}_{2}\mathfrak {p}_{\nu _{1}(s)}\cdots \mathfrak {p}_{\nu _{\mathfrak {D}-2}(s)} \end{aligned}$$

where $\mathfrak {q}_{2}$ is the projection onto the $V^{\mu }\otimes \mathrm {triv}$-isotypic subspace for

$$\begin{aligned} S_{b}\times S_{\omega _{s}(m+1)}\times S_{\omega _{s}(m+2)}\times \cdots \times S_{\omega _{s}(m+\mathfrak {D}-1)}. \end{aligned}$$

This gives

$$\begin{aligned} \dim \left( \pi _{\uplambda }(Q)V^{\uplambda }\right) =\dim (\pi _{\nu _{\mathfrak {D}-1}(s)}(Q_{2})V^{\nu _{\mathfrak {D}-1}(s)}). \end{aligned}$$

Iterating this argument, using Pieri’s formula each time, gives eventually that

$$\begin{aligned} \dim \left( \pi _{\uplambda }(Q)V^{\uplambda }\right) =\dim (\pi _{\mu }(\mathfrak {p}_{\mu })V^{\mu })=d_{\mu }. \end{aligned}$$

Thus, in total, going back to (4.29), we obtain the required

$$\begin{aligned} \dim \left( \pi _{\uplambda '}(Q)V^{\uplambda '}\right) =d_{\uplambda '/\uplambda }d_{\mu }. \end{aligned}$$

$$\square $$

#### Proof of Proposition 4.9

Pick an orthonormal basis $\{v_{1},\ldots ,v_{D}\}$ for $\pi _{\uplambda '}(Q)V^{\uplambda '}$ with $D=d_{\uplambda '/\uplambda }d_{\mu }$ and extend this to an orthonormal basis of $V^{\uplambda '}$. In regard to (4.27), we have

4.30 $$\begin{aligned}&\Vert \mathfrak {p}_{\nu _{\mathfrak {D}}(t)}\cdots \mathfrak {p}_{\nu _{1}(t)}\sum _{[\tau ]\in S_{\Pi }/S_{s}}(\mathrm {id}_{S_{b}},\pi _{0}\tau )\mathfrak {q}_{s}\mathfrak {p}_{\nu _{1}(s)}\cdots \mathfrak {p}_{\nu _{\mathfrak {D}}(s)}\Vert _{V^{\uplambda '}}^{2}\nonumber \\&\quad = \Vert \mathfrak {p}_{\nu _{\mathfrak {D}}(t)}\cdots \mathfrak {p}_{\nu _{1}(t)}\sum _{[\tau ]\in S_{\Pi }/S_{s}}(\mathrm {id}_{S_{b}},\pi _{0}\tau )Q\Vert _{V^{\uplambda '}}^{2}\nonumber \\&\quad = \sum _{i=1}^{D}\Vert \pi _{\uplambda '}(\mathfrak {p}_{\nu _{\mathfrak {D}}(t)}\cdots \mathfrak {p}_{\nu _{1}(t)})\sum _{[\tau ]\in S_{\Pi }/S_{s}}\pi _{\uplambda '}(\mathrm {id}_{S_{b}},\pi _{0}\tau )\pi _{\uplambda '}(Q)v_{i}\Vert ^{2}\nonumber \\&\quad = \sum _{i=1}^{D}\Vert \pi _{\uplambda '}(\mathfrak {p}_{\nu _{\mathfrak {D}}(t)}\cdots \mathfrak {p}_{\nu _{1}(t)})\sum _{[\tau ]\in S_{\Pi }/S_{s}}\pi _{\uplambda '}(\mathrm {id}_{S_{b}},\pi _{0}\tau )v_{i}\Vert ^{2}. \end{aligned}$$

Now, each $\pi _{\uplambda '}(\mathrm {id}_{S_{b}},\pi _{0}\tau )$ is unitary, and by the second statement of Lemma 4.10, $\pi _{\uplambda '}(\mathfrak {p}_{\nu _{\mathfrak {D}}(t)}\cdots \mathfrak {p}_{\nu _{1}(t)})$ is a self-adjoint idempotent and hence has operator norm bounded by one. Hence, for $i\in [D]$

$$\begin{aligned} \Vert \pi _{\uplambda '}(\mathfrak {p}_{\nu _{\mathfrak {D}}(t)}\cdots \mathfrak {p}_{\nu _{1}(t)})\sum _{[\tau ]\in S_{\Pi }/S_{s}}\pi _{\uplambda '}(\mathrm {id}_{S_{b}},\pi _{0}\tau )v_{i}\Vert \le [S_{\Pi }:S_{s}]. \end{aligned}$$

Combining this with (4.30), we obtain

$$\begin{aligned} \Vert \mathfrak {p}_{\nu _{\mathfrak {D}}(t)}\cdots \mathfrak {p}_{\nu _{1}(t)}\sum _{[\tau ]\in S_{\Pi }/S_{s}}(\mathrm {id}_{S_{b}},\pi _{0}\tau )\mathfrak {q}_{s}\mathfrak {p}_{\nu _{1}(s)}\cdots \mathfrak {p}_{\nu _{\mathfrak {D}}(s)}\Vert _{V^{\uplambda '}}&\le \sqrt{D}[S_{\Pi }:S_{s}]\\&=\sqrt{d_{\uplambda '/\uplambda }d_{\mu }}[S_{\Pi }:S_{s}]. \end{aligned}$$

Using this in (4.27) gives

$$\begin{aligned} \Vert {\hat{P}}[A_{s,t}^{\uplambda ,\mu }\otimes X]\Vert _{\uplambda '}&\le \frac{b!}{d_{\mu }}\frac{|S_{s}|}{(B+p)!}D_{\mu }(m)\frac{d_{\uplambda '}}{\sqrt{D_{\uplambda '}(n)}}\sqrt{d_{\uplambda '/\uplambda }d_{\mu }}[S_{\Pi }:S_{s}]\\&=\frac{b!}{\sqrt{d_{\mu }}}\frac{|S_{\Pi }|}{(B+p)!}D_{\mu }(m)\frac{d_{\uplambda '}}{\sqrt{D_{\uplambda '}(n)}}\sqrt{d_{\uplambda '/\uplambda }}. \end{aligned}$$

Finally incorporating this estimate into (4.26) gives

$$\begin{aligned} \Vert {\hat{P}}[A_{s,t}^{\uplambda ,\mu }\otimes X]\Vert ^{2}\le \frac{(b!)^{2}|S_{\Pi }|^{2}D_{\mu }(m)^{2}}{d_{\mu }((B+p)!)^{2}}\sum _{\begin{array}{c} \uplambda '\vdash B+p\\ \ell (\uplambda ')\le n \end{array} }\frac{d_{\uplambda '}^{2}d_{\uplambda '/\uplambda }}{D_{\uplambda '}(n)}. \end{aligned}$$

$$\square $$

### Completing the outlined strategy

#### Proposition 4.12

With notation as in the previous sections,

$$\begin{aligned} \Vert P[\mathcal {E}_{s,t}^{\uplambda ,\mu ,m}\otimes X]\Vert ^{2}\le (|\uplambda /\mu |+p)^{2p}\frac{D_{\mu }(m)}{D_{\uplambda }(n)}. \end{aligned}$$

#### Proof

From the argument of our strategy in §§4.3, we have

4.31 $$\begin{aligned} \Vert P[\mathcal {E}_{s,t}^{\uplambda ,\mu ,m}\otimes X]\Vert ^{2}=\frac{\Vert {\hat{P}}[A_{s,t}^{\uplambda ,\mu }\otimes X]\Vert ^{2}}{\Vert A_{s,t}^{\uplambda ,\mu }\Vert ^{2}}. \end{aligned}$$

If (4.22) fails to hold, then Propositions 4.8 and 4.9 give $\Vert P[\mathcal {E}_{s,t}^{\uplambda ,\mu ,m}\otimes X]\Vert =0$, proving Proposition 4.12 in this instance.

Now suppose (4.22) holds. Then Propositions 4.8 and 4.9 combined with (4.31) give

4.32 $$\begin{aligned} \Vert P[\mathcal {E}_{s,t}^{\uplambda ,\mu ,m}\otimes X]\Vert ^{2}&\le \left( D_{\mu }(m)\frac{d_{\uplambda }^{2}(b!)^{2}}{d_{\mu }(B!)^{2}}\prod _{i=1}^{\mathfrak {D}}\omega _{t}(m+i)!\omega _{s}(m+i)!\right) ^{-1}\nonumber \\&\quad \frac{(b!)^{2}|S_{\Pi }|^{2}D_{\mu }(m)^{2}}{d_{\mu }((B+p)!)^{2}}\sum _{\begin{array}{c} \uplambda \subset _{p}\uplambda '\\ \ell (\uplambda ')\le n \end{array} }\frac{d_{\uplambda '}^{2}d_{\uplambda '/\uplambda }}{D_{\uplambda '}(n)}\nonumber \\&=\frac{|S_{\Pi }|^{2}}{\prod _{i=1}^{\mathfrak {D}}\omega _{t}(m+i)!\omega _{s}(m+i)!}D_{\mu }(m)\frac{(B!)^{2}}{((B+p)!)^{2}}\sum _{\begin{array}{c} \uplambda \subset _{p}\uplambda '\\ \ell (\uplambda ')\le n \end{array} }\frac{d_{\uplambda '}^{2}d_{\uplambda '/\uplambda }}{d_{\uplambda }^{2}D_{\uplambda '}(n)}. \end{aligned}$$

Recalling that the block sizes of $\Pi$ are given by either side of (4.22), we have

4.33 $$\begin{aligned} \frac{|S_{\Pi }|^{2}}{\prod _{i=1}^{\mathfrak {D}}\omega _{t}(m+i)!\omega _{s}(m+i)!}&=\prod _{i=1}^{\mathfrak {D}}\frac{(\omega _{t}(m+i)+p_{i})!}{\omega _{t}(m+i)!}\frac{(\omega _{s}(m+i)+q_{i})!}{\omega _{s}(m+i)!}\nonumber \\&\le \prod _{i=1}^{\mathfrak {D}}(\omega _{t}(m+i)+p_{i})^{p_{i}}(\omega _{s}(m+i)+q_{i})^{q_{i}}\nonumber \\&\le \prod _{i=1}^{\mathfrak {D}}(|\uplambda /\mu |+p)^{p_{i}}(|\uplambda /\mu |+p)^{q_{i}}\nonumber \\&\le (|\uplambda /\mu |+p)^{2p} \end{aligned}$$

using that $$\sum _{i=1}^{\mathfrak {D}}p_{i}=\sum _{i=1}^{\mathfrak {D}}q_{i}=p$$.

Next, by using twice both the hook length formula (2.2) and the hook content formula (2.5) we obtain

4.34 $$\begin{aligned} \frac{(B!)^{2}}{((B+p)!)^{2}}\sum _{\begin{array}{c} \uplambda \subset _{p}\uplambda '\\ \ell (\uplambda ')\le n \end{array} }\frac{d_{\uplambda '}^{2}d_{\uplambda '/\uplambda }}{d_{\uplambda }^{2}D_{\uplambda '}(n)}&=\frac{(B!)}{(B+p)!}\frac{1}{d_{\uplambda }D_{\uplambda }(n)}\sum _{\begin{array}{c} \uplambda \subset _{p}\uplambda '\\ \ell (\uplambda ')\le n \end{array} }\frac{\prod _{\square \in \uplambda }(n+c(\square ))}{\prod _{\square \in \uplambda '}(n+c(\square ))}d_{\uplambda '}d_{\uplambda '/\uplambda }\nonumber \\&\le \frac{B!}{(B+p)!}\frac{1}{d_{\uplambda }D_{\uplambda }(n)}\sum _{\begin{array}{c} \uplambda \subset _{p}\uplambda '\\ \ell (\uplambda ')\le n \end{array} }d_{\uplambda '}d_{\uplambda '/\uplambda }\nonumber \\&\le \frac{1}{D_{\uplambda }(n)}, \end{aligned}$$

where the last inequality used (2.3). Inserting (4.33) and (4.34) into (4.32) gives the result stated in the proposition. $\quad \square$

### Proof of Theorem 4.1

Following our strategy, applying Proposition 4.12 to both $P[\mathcal {E}_{s,t}^{\uplambda ,\mu ,m}\otimes X]$ and $P[\mathcal {E}_{s',t'}^{\uplambda ,\mu ,m}\otimes X']$ and using the Schwarz inequality we obtain the following

$$\begin{aligned} |(4.10)|\le (|\uplambda /\mu |+p)^{2p}\frac{D_{\mu }(m)}{D_{\uplambda }(n)}. \end{aligned}$$

Therefore, for any $I\subset [m+1,n]^{|w|}$, using the argument from the beginning of §§4.3, writing *f* for an element of $\{a,b,c,d\}$, we have

4.35 $$\begin{aligned}&|\int _{h\in \mathsf {U}(n)^{4}}w_{I}(h)\langle h[\mathcal {E}_{s_{1},T_{2}}^{\uplambda ,\mu ,m}\otimes \mathcal {E}_{S_{1},s_{1}}^{\uplambda ,\mu ,m}\otimes \mathcal {E}_{s_{2},T_{4}}^{\uplambda ,\mu ,m}\otimes \mathcal {E}_{\mu ,S_{3},s_{2}}^{\uplambda ,\mu ,m}],\nonumber \\&\quad \mathcal {E}_{S_{1},S_{4}}^{\uplambda ,\mu ,m}\otimes \mathcal {E}_{S_{2},T_{2}}^{\uplambda ,\mu ,m}\otimes \mathcal {E}_{S_{3},S_{2}}^{\uplambda ,\mu ,m}\otimes \mathcal {E}_{S_{4},T_{4}}^{\uplambda ,\mu ,m}\rangle dh|\nonumber \\ \le&\frac{D_{\mu }(m)^{4}}{D_{\uplambda }(n)^{4}}\prod _{f\in \{a,b,c,d\}}(|\uplambda /\mu |+p_{f})^{2p_{f}}\nonumber \\ \le&\frac{D_{\mu }(m)^{4}}{D_{\uplambda }(n)^{4}}\left( |\uplambda /\mu |+|w|\right) ^{8|w|} \end{aligned}$$

where the last inequality used

$$\begin{aligned} (|\uplambda /\mu |+p_{f})^{2p_{f}}\le \left( |\uplambda /\mu |+|w|\right) ^{2|w|} \end{aligned}$$

for each $f\in \{a,b,c,d\}$. (For general *g* the exponents 8|*w*| and 4 in (4.35) are 4*g*|*w*| and 2*g*, respectively.)

Next using Proposition 4.5, we obtain for $m(I)=n-\mathfrak {D}(I)$

$$\begin{aligned} \mathcal {I}^{*}(w_{I},\uplambda )\le&\sum _{\begin{array}{c} \mu \subset ^{\mathfrak {D}(I)}\uplambda \\ \ell (\mu )\le m(I) \end{array} }\frac{1}{D_{\mu }(m(I))^{3}}\sum _{S_{1},S_{2},S_{3},S_{4},T_{2},T_{4},s_{1},s_{2}\in \mathcal {SST}_{[m(I)+1,n]}(\uplambda /\mu )}\\&\quad \frac{D_{\mu }(m(I))^{4}}{D_{\uplambda }(n)^{4}}\left( |\uplambda /\mu |+|w|\right) ^{8|w|}\\ =&\frac{1}{D_{\uplambda }(n)^{4}}\sum _{\begin{array}{c} \mu \subset ^{\mathfrak {D}(I)}\uplambda \\ \ell (\mu )\le m(I) \end{array} }D_{\mu }(m)\left( |\uplambda /\mu |+|w|\right) ^{8|w|}|\mathcal {SST}_{[m(I)+1,n]}(\uplambda /\mu )|^{8}. \end{aligned}$$

(For general *g*, $|\mathcal {SST}_{[m(I)+1,n]}(\uplambda /\mu )|^{8}$ is replaced by $|\mathcal {SST}_{[m(I)+1,n]}(\uplambda /\mu )|^{4g}$.) Hence, from (4.3) we obtain Theorem 4.1. $\square$

## The Total Contribution from Large-Dimensional Families

### Statement of main sectional results

The main task of this section is to estimate the sum

$$\begin{aligned} \sum _{\begin{array}{c} {{({\rho ,W)\in \widehat{\mathsf {SU}(n)}}\backslash \Omega (B;n)}} \end{array} }(\dim W){\mathcal {I}}(w,\rho ) \end{aligned}$$

appearing in the left-hand side of (1.10). Let $\Lambda (B;n)$ denote the collection of Young diagrams of length at most $n-1$ such that

$$\begin{aligned} \uplambda \in \Lambda (B;n)\mapsto (\rho _{n}^{\uplambda },W_{n}^{\uplambda })\in \widehat{\mathsf {SU}(n)}\backslash \Omega (B;n) \end{aligned}$$

is a one-to-one parametrization. The goal of this section is to prove Theorem 1.9, which amounts to establishing a bound for

$$\begin{aligned} \Sigma _{2}(w,B,n){\mathop {=}\limits ^{\mathrm {def}}}\sum _{\uplambda \in \Lambda (B;n)}D_{\uplambda }(n)\mathcal {I}(w,\uplambda ) \end{aligned}$$

and proving its absolute convergence.

Theorem 1.9 has the following corollary that will be useful later.

#### Corollary 5.1

Let $g\ge 2$ and $B\in \mathbf {N}$ be fixed. We have

$$\begin{aligned} \zeta (2g-2;n)=\sum _{(\rho ,W)\in \Omega (B;n)}\frac{1}{(\dim W)^{2g-2}}+O_{g,B}\left( \frac{1}{n}\left( n^{-2\log B}\right) \right) \end{aligned}$$

as $$n\rightarrow \infty $$.

#### Proof of Corollary 5.1

given Theorem 1.9. Let $w=\mathrm {id}_{\mathrm {\mathbf {F}} _{2g}}.$ We use the result, for any $(\rho ,W)\in \widehat{\mathsf {SU}(n)}$

5.1 $$\begin{aligned} \int \overline{\mathrm {tr} (\rho ((R_{g}(x))))}d\mu _{\mathsf {SU}(n)^{2g}}^{\mathrm {Haar}}(x)=\frac{1}{(\dim W)^{2g-1}} \end{aligned}$$

which is due to Frobenius [Fro96] when $g=1$; see [PS14, §2] or [CMP21, eq. (2.2)] for the (easy) extension to general $g\ge 2.$ Since $\mathrm {tr} (\mathrm {id}_{\mathrm {\mathbf {F}} _{2g}}(x))=n$ for all $x\in \mathsf {SU}(n)^{2g}$, we obtain from (5.1) that for any $(\rho ,W)\in \widehat{\mathsf {SU}(n)}$, $\mathcal {I}(w,\rho )=\frac{n}{(\dim W)^{2g-1}}$; hence,

5.2 $$\begin{aligned} \Sigma _{2}(\mathrm {id}_{\mathrm {\mathbf {F}} _{2g}},B,n)&=n\sum _{(\rho ,W)\in \widehat{\mathsf {SU}(n)}\backslash \Omega (B;n)}\frac{1}{(\dim W)^{2g-2}}. \end{aligned}$$

Now the corollary follows directly^7^ from Theorem 1.9. $\quad \square$

### Preliminary estimates

Even in the simple case of $\gamma =\mathrm {id}$ (5.2) shows that the problem of estimating $\Sigma _{2}$ is related to the large-*n* convergence of the Witten zeta function as in Theorem 1.8. The techniques used in [GLM12] can be adapted to deal with (5.2), and indeed, are also useful in dealing with general $\gamma$ (or *w*). The key estimate we take from *(ibid.) *is the following. Given a YD $\uplambda$ with $\ell (\uplambda )<n$, we define $x_{i}(\uplambda ){\mathop {=}\limits ^{\mathrm {def}}}\uplambda _{i}-\uplambda _{i+1}$, setting $\uplambda _{i}=0$ for $i>\ell (\uplambda )$. These are the coefficients of the highest weight of $(\rho _{n}^{\uplambda },W_{n}^{\uplambda })$ with respect to a system of fundamental weights for $\mathsf {SU}(n)$.

#### Lemma 5.2

([GLM12, eq. (1), Lemma 8]). For a YD $\uplambda$ with $\ell (\uplambda )<n$, we have

$$\begin{aligned} D_{\uplambda }(n)\ge \prod _{i=1}^{n-1}(1+x_{i}(\uplambda ))^{v_{i}} \end{aligned}$$

where $v_{i}$ are positive real numbers satisfying for $0\le j\le \frac{n}{2}$

$$\begin{aligned} v_{j}=v_{n-j}\ge j\max (1,\log (n-1)-\log j). \end{aligned}$$

For the reader’s convenience, Lemma 5.2 follows from applying the AM–GM inequality to the Weyl dimension formula. It will turn out, because of Lemma 5.2, to be useful to work with the coordinates $\mathbf {x}(\uplambda )\in \mathbf {N}_{0}^{n-1}$ from now on, instead of $\uplambda$. On the other hand, Theorem 4.1 involves quantities $|\uplambda /\mu |$ and $|\mathcal {SST}_{[m+1,n]}(\uplambda /\mu )|$ for $\mu \subset ^{\mathfrak {D}}\uplambda$, $m+\mathfrak {D}=n$. We now estimate these quantities in terms of $\mathbf {x}(\uplambda )$.

#### Lemma 5.3

If $\mathfrak {D}\in \mathbf {N}_{0}$, $\uplambda$ and $\mu$ are YDs with $\ell (\uplambda )\le n-1$ and $\mu \subset ^{\mathfrak {D}}\uplambda$, then

$$\begin{aligned} |\uplambda /\mu |\le \mathfrak {D}\sum _{j=1}^{n-1}x_{j}(\uplambda )\le \mathfrak {D}\prod _{j=1}^{n-1}(1+x_{j}(\uplambda )). \end{aligned}$$

#### Proof

The condition that $\mu \subset ^{\mathfrak {D}}\uplambda$ means that there is a chain of YDs $\mu =\mu ^{\mathfrak {D}}\subset ^{1}\mu ^{\mathfrak {D}-1}\subset ^{1}\ldots \subset \mu ^{1}\subset ^{1}\mu _{0}=\uplambda$. First note that $|\uplambda /\mu ^{1}|\le \uplambda _{1}$ since to obtain $\mu ^{1}$ from $\uplambda$, one can delete at most one box from each column and there are $\uplambda _{1}$ non-empty columns of $\uplambda$. One also has $\mu _{1}^{1}\le \uplambda _{1}$ and so repeating the argument gives $|\mu ^{1}/\mu ^{2}|\le \mu _{1}\le \uplambda _{1}$ and iterating further gives $|\mu ^{i}/\mu ^{i+1}|\le \uplambda _{1}$ for all $0\le i\le \mathfrak {D}-1$. Hence,

$$\begin{aligned} |\uplambda /\mu |=\sum _{i=0}^{\mathfrak {D}-1}|\mu ^{i}/\mu ^{i+1}|\le \mathfrak {D}\uplambda _{1}=\mathfrak {D}\sum _{j=1}^{n-1}x_{i}(\uplambda ). \end{aligned}$$

$$\square $$

#### Lemma 5.4

If $\mathfrak {D}\in \mathbf {N}_{0}$, $n\ge \mathfrak {D}$, $\uplambda$ and $\mu$ are YDs with $\ell (\uplambda )\le n-1$ and $\mu \subset ^{\mathfrak {D}}\uplambda$, and $m=n-\mathfrak {D}$, then

$$\begin{aligned} |\mathcal {SST}_{[m+1,n]}(\uplambda /\mu )|\le \left( \prod _{i=1}^{n-1}(x_{i}(\uplambda )+1)\right) ^{2^{\mathfrak {D}}}. \end{aligned}$$

#### Proof

Choosing an element of $\mathcal {SST}_{[m+1,n]}(\uplambda /\mu )$ is the same as choosing a sequence $\mu =\mu ^{\mathfrak {D}}\subset ^{1}\mu ^{\mathfrak {D}-1}\subset ^{1}\ldots \subset \mu ^{1}\subset ^{1}\mu _{0}=\uplambda$. We regard all $\mathbf {x}(\mu ^{i})\in \mathbf {N}_{0}^{n-1}$ by extending by zeros. The number of choices of $\mu ^{1}$ is $\prod _{i=1}^{n-1}(x_{i}(\uplambda )+1)$ since one removes from the $i^{th}$ row of $\uplambda$ between 0 and $x_{i}(\uplambda )$ boxes inclusive. Regardless of how $\mu ^{1}$ is chosen, we have

$$\begin{aligned} x_{i}(\mu ^{1})=\mu _{i}^{1}-\mu _{i+1}^{1}\le \uplambda _{i}-\uplambda _{i+2}=x_{i}(\uplambda )+x_{i+1}(\uplambda )\le (1+x_{i}(\uplambda ))(1+x_{i+1}(\uplambda ))-1 \end{aligned}$$

so

$$\begin{aligned} \prod _{i=1}^{n-1}(x_{i}(\mu )+1)\le \left( \prod _{i=1}^{n-1}(x_{i}(\uplambda )+1)\right) ^{2}. \end{aligned}$$

Thus, repeating the previous argument gives that the number of choices of $\mu ^{2}$, given $\mu ^{1}$, is at most $\left( \prod _{i=1}^{n-1}(x_{i}(\uplambda )+1)\right) ^{2}$. Iterating further, the number of choices of the chain $\mu ^{1},\mu ^{2},\ldots ,\mu ^{\mathfrak {D}-1}$ is at most

$$\begin{aligned}&\prod _{i=1}^{n-1}(x_{i}(\uplambda )+1)\left( \prod _{i=1}^{n-1}(x_{i}(\uplambda )+1)\right) ^{2}\left( \prod _{i=1}^{n-1}(x_{i}(\uplambda )+1)\right) ^{4}\cdots \left( \prod _{i=1}^{n-1}(x_{i}(\uplambda )+1)\right) ^{2^{\mathfrak {D}-2}}\\ =&\left( \prod _{i=1}^{n-1}(x_{i}(\uplambda )+1)\right) ^{\sum _{k=0}^{\mathfrak {D}-2}2^{k}}\le \left( \prod _{i=1}^{n-1}(x_{i}(\uplambda )+1)\right) ^{2^{\mathfrak {D}}}. \end{aligned}$$

$$\square $$

We can use Lemmas 5.3 and Lemma 5.4 to tidy up Theorem 4.1.

#### Proposition 5.5

For $w\in [\mathrm {\mathbf {F}} _{2g},\mathrm {\mathbf {F}} _{2g}]$, we have

$$\begin{aligned} |\mathcal {I}(w,\uplambda )|\ll _{w,g}n^{|w|}\frac{\left( \prod _{j=1}^{n-1}(1+x_{j}(\uplambda ))\right) }{D_{\uplambda }(n)^{2g-1}}^{C(w,g)} \end{aligned}$$

as $$n\rightarrow \infty $$, where

$$\begin{aligned} C(w,g){\mathop {=}\limits ^{\mathrm {def}}}4g|w|(1+2^{|w|}). \end{aligned}$$

#### Proof

Recalling that $\mathfrak {D}(I)$ is the number of distinct indices of some $I\in [n]^{|w|}$, we have $\mathfrak {D}(I)\le |w|$. We begin by using Lemmas 5.3 and 5.4 in Theorem 4.1 to obtain

$$\begin{aligned} |\mathcal {I}(w,\uplambda )|&\le \sum _{[I]\in S_{_{n}}\backslash [n]^{|w|}}(n)_{\mathfrak {D}(I)}\\&\quad \frac{1}{D_{\uplambda }(n)^{2g}}\sum _{\begin{array}{c} \mu \subset ^{\mathfrak {D}(I)}\uplambda \\ \ell (\mu )\le n-\mathfrak {D}(I) \end{array} }D_{\mu }(n-\mathfrak {D}(I))\left( |\uplambda /\mu |+|w|\right) ^{4g|w|}\\&\quad |\mathcal {SST}_{[n-\mathfrak {D}(I)+1,n]}(\uplambda /\mu )|^{4g|w|}\\&\ll _{w,g}\sum _{[I]\in S_{_{n}}\backslash [n]^{|w|}}n^{\mathfrak {D}(I)}\frac{\left( \prod _{j=1}^{n-1}(1+x_{j}(\uplambda ))\right) ^{4g|w|(1+2^{|w|})}}{D_{\uplambda }(n)^{2g}}\\&\sum _{\begin{array}{c} \mu \subset ^{\mathfrak {D}(I)}\uplambda \\ \ell (\mu )\le n-\mathfrak {D}(I) \end{array} }D_{\mu }(n-\mathfrak {D}(I)). \end{aligned}$$

Here the notation $\ll _{w}$ is with respect to $n\rightarrow \infty$. Since given $\uplambda$ with $\ell (\uplambda )\le n-1$, for every $\mu \subset ^{\mathfrak {D}(I)}\uplambda$ with $\ell (\mu )\le n-\mathfrak {D}(I)$, $\dim \mathrm {Hom}_{\mathsf {U}(n-\mathfrak {D}(I))}(W_{n-\mathfrak {D}(I)}^{\mu },W_{n}^{\uplambda })\ge 1$ by the branching rules, we have

$$\begin{aligned} \sum _{\begin{array}{c} \mu \subset ^{\mathfrak {D}(I)}\uplambda \\ \ell (\mu )\le n-\mathfrak {D}(I) \end{array} }D_{\mu }(n-\mathfrak {D}(I))\le D_{\uplambda }(n); \end{aligned}$$

hence,

$$\begin{aligned} |\mathcal {I}(w,\uplambda )|\ll _{w,g}\sum _{[I]\in S_{_{n}}\backslash [n]^{|w|}}n^{\mathfrak {D}(I)}\frac{\left( \prod _{j=1}^{n-1}(1+x_{j}(\uplambda ))\right) ^{4g|w|(1+2^{|w|})}}{D_{\uplambda }(n)^{2g-1}}. \end{aligned}$$

Moreover,

$$\begin{aligned} |S_{_{n}}\backslash [n]^{|w|}|\le |w|^{|w|}, \end{aligned}$$

so we obtain

5.3 $$\begin{aligned} |\mathcal {I}(w,\uplambda )|\ll _{w,g}n^{|w|}\frac{\left( \prod _{j=1}^{n-1}(1+x_{j}(\uplambda ))\right) }{D_{\uplambda }(n)^{2g-1}}^{4g|w|(1+2^{|w|})}=n^{|w|}\frac{\left( \prod _{j=1}^{n-1}(1+x_{j}(\uplambda ))\right) }{D_{\uplambda }(n)^{2g-1}}^{C(w,g)} \end{aligned}$$

as required. $$\quad \square $$

### Proof of Theorem 1.9

We begin by considering the sum $\Sigma _{2}(w,B,n)$

$=\sum _{\uplambda \in \Lambda (B;n)}D_{\uplambda }(n)\mathcal {I}(w,\uplambda )$. Fix $g\ge 2$. We use Proposition 5.5 in this sum to obtain

$$\begin{aligned} \Sigma _{2}(w,B,n)\ll _{w,g}n^{|w|}\sum _{\uplambda \in \Lambda (B;n)}\frac{\left( \prod _{j=1}^{n-1}(1+x_{j}(\uplambda ))\right) }{D_{\uplambda }(n)^{2g-2}}^{C(w,g)} \end{aligned}$$

where $C(w,g)>0$. Let $C=C(w,g)$. We need a key observation relating the weight coefficients $\mathbf {x}(\uplambda )$ to the set $\Omega (B;n)$ and hence $\Lambda (B;n)$. It is not hard to check that if $\uplambda \in \Lambda (B;n)$ then either

- $x_{i}(\uplambda )>B$ for some $i\le B$ or $i\ge n-B$ **or**
- $$x_{i}(\uplambda )>0$$ for some $$B<i<n-B$$,

and given $\mathbf {x}\in \mathbf {Z}_{\ge 0}^{n-1}$ satisfying these conditions there is at most one corresponding $\uplambda \in \Lambda (B;n)$. (Obtaining these conditions was the reason for the original choice of $\Omega (B,n)$.)

Now by Lemma 5.2

5.4 $$\begin{aligned} \Sigma _{2}(w,B,n)&\ll _{w,g}n^{|w|}\sum _{\uplambda \in \Lambda (B;n)}\frac{1}{\prod _{j=1}^{n-1}(1+x_{j}(\uplambda ))^{(2g-2)v_{j}-C}}\nonumber \\&\le n^{|w|}\sum _{\uplambda \in \Lambda (B;n)}\frac{1}{\prod _{j=1}^{n-1}(1+x_{j}(\uplambda ))^{2v_{j}-C}} \end{aligned}$$

5.5 $$\begin{aligned}&\le n^{|w|}\sum _{\begin{array}{c} \mathbf {x}\in \mathbf {N}_{0}^{n-1}:\\ x_{j}>0\text { for some }B<j<n-B{ \mathbf{or} }\\ x_{j}>B\text { for some }j\in [B]\cup [n-B,n-1] \end{array} }\frac{1}{\prod _{j=1}^{n-1}(1+x_{j}(\uplambda ))^{2v_{j}-C}}. \end{aligned}$$

Here $v_{j}$ are the constants from Lemma 5.2 satisfying for $0\le j\le \frac{n}{2}$

$$\begin{aligned} v_{j}=v_{n-j}\ge j\max (1,\log (n-1)-\log j). \end{aligned}$$

Notice that if $j\in [\frac{n-1}{e},n-\frac{n-1}{e}]$ we have $v_{j}\ge \frac{n-1}{e}$. The function $x\mapsto x\log \left( \frac{n-1}{x}\right)$ has nonnegative derivative on $[1,\frac{n-1}{e}]$, so the minimum value of $v_{j}$ for $j\in [1,\frac{n-1}{e}]\cup [n-1-\frac{n-1}{e},n-1]$ is $v_{1}\ge \log (n-1)$. We have $\frac{n-1}{e}\ge \log (n-1)$ for $n\gg 1$ so for $n\gg 1$ we have

5.6 $$\begin{aligned} v_{j}\ge \log (n-1),\quad j\in [n-1]. \end{aligned}$$

The sum in (5.5) can be crudely estimated by disregarding the constraints on $\mathbf {x}$ to obtain for $n\gg 1$

5.7 $$\begin{aligned} \Sigma _{2}(w,B,n)\ll _{w}n^{|w|}\prod _{j=1}^{n-1}\zeta (2v_{j}-C) \end{aligned}$$

where for $$\mathrm {Re}(s)>1$$

$$\begin{aligned} \zeta (s){\mathop {=}\limits ^{\mathrm {def}}}\sum _{k=1}^{\infty }\frac{1}{n^{s}} \end{aligned}$$

is an absolutely convergent sum defining *Riemann’s zeta function. *Notice that for $n\gg _{w,g}1$, $2v_{j}-C\ge 2\log (n-1)-C>1$, so (5.7) shows that $\Sigma _{2}(w,B,n)$ is defined by an absolutely convergent sum. *This proves the first statement of Theorem* 1.9.

We now turn to finer estimates for $\Sigma _{2}$. Assume $n\ge 2B\max (2B,C+1)$ and $2\log (n-1)-C>2$, so that $2v_{j}-C\ge 3$ .

Incorporating the constraints on $\mathbf {x}$ in (5.5) gives the improved estimate

$$\begin{aligned} \frac{\Sigma _{2}(w,B,n)}{n^{|w|}}&\ll _{w,g}\sum _{j=1}^{B}\zeta ^{(B+1)}(2v_{j}-C)\prod _{\begin{array}{c} i\in [n-1],\,i\ne j \end{array} }\zeta (2v_{i}-C)\\&+\sum _{j=B+1}^{n-B-1}\zeta ^{(2)}(2v_{j}-C)\prod _{\begin{array}{c} i\in [n-1],\,i\ne j \end{array} }\zeta (2v_{i}-C)\\&+\sum _{j=n-B}^{n-1}\zeta ^{(B+1)}(2v_{j}-C)\prod _{\begin{array}{c} i\in [n-1],\,i\ne j \end{array} }\zeta (2v_{i}-C)\\&\ll \left( \sum _{j=1}^{B}\zeta ^{(B+1)}(2v_{j}-C)+\sum _{j=B+1}^{\lfloor \frac{n}{2}\rfloor }\zeta ^{(1)}(2v_{j}-C)\right) \prod _{\begin{array}{c} i\in [n-1] \end{array} }\zeta (2v_{i}-C) \end{aligned}$$

where the last estimate used $v_{j}=v_{n-j}$ and $\zeta (2-C)\ge 1$, and

$$\begin{aligned} \zeta ^{(p)}(s){\mathop {=}\limits ^{\mathrm {def}}}\sum _{k=p}^{\infty }\frac{1}{n^{s}}. \end{aligned}$$

Moreover, for $s\ge 3$ the simple bound [GLM12, pg. 1826] $\zeta (s)\le 1+2\cdot 2^{-s}$ and our assumptions on *n* imply

$$\begin{aligned} \log (\prod _{\begin{array}{c} i\in [n-1] \end{array} }\zeta (2v_{i}-C))&\le 2\sum _{j=1}^{\lfloor \frac{n}{2}\rfloor }\log (1+2\cdot 2^{-2v_{j}+C})\\&\le 4\sum _{j=1}^{\lfloor \frac{n}{2}\rfloor }2^{-2v_{j}+C}\\&\le 2^{C+2}\left( \sum _{1\le j\le \lfloor \frac{n}{2}\rfloor }2^{-2j}\right) \ll _{w}1. \end{aligned}$$

Hence,

5.8 $$\begin{aligned} \frac{\Sigma _{2}(w,B,n)}{n^{|w|}}\ll _{w}\sum _{j=1}^{B}\zeta ^{(B+1)}(2v_{j}-C)+\sum _{j=B+1}^{\lfloor \frac{n}{2}\rfloor }\zeta ^{(2)}(2v_{j}-C). \end{aligned}$$

We next need bounds for the $\zeta ^{(p)}$. By comparison with an integral, we have for $s>1$ and $p\ge 2$

$$\begin{aligned} \zeta ^{(p)}(s)\le \frac{(p-1)^{1-s}}{s-1}. \end{aligned}$$

This gives

5.9 $$\begin{aligned} \sum _{j=1}^{B}\zeta ^{(B+1)}(2v_{j}-C)&\le \sum _{j=1}^{B}\frac{B^{1+C-2v_{j}}}{2v_{j}-C}\le \frac{1}{2}B^{1+C}\sum _{j=1}^{B}B^{-2v_{j}}\nonumber \\&\ll _{B,w}\sum _{j=1}^{B}B^{-2j\log \left( \frac{n-1}{j}\right) }\le \sum _{j=1}^{B}B^{-2j\log \left( \frac{n-1}{B}\right) }\nonumber \\&\ll _{B}B^{-2\log \left( \frac{n-1}{B}\right) }=\left( \frac{n-1}{B}\right) ^{-2\log B}\ll _{B}(n-1)^{-2\log B}. \end{aligned}$$

To deal with the second part of (5.8), we use a different bound. One has the bound [GLM12, proof of Lemma 6]

$$\begin{aligned} \zeta ^{(2)}(s)\le 2\cdot 2^{-s} \end{aligned}$$

for $s\ge 3$. Under our current assumptions on *n*, this gives

5.10 $$\begin{aligned} \sum _{j=B+1}^{\lfloor \frac{n}{2}\rfloor }\zeta ^{(2)}(2v_{j}-C)&\le 2\sum _{j=B+1}^{\lfloor \frac{n}{2}\rfloor }2^{C-2v_{j}}\ll _{w,g}\sum _{j=B+1}^{\lfloor \frac{n}{2}\rfloor }2^{-2v_{j}}\nonumber \\&\le \sum _{j=B+1}^{\lceil \sqrt{n-1}\rceil }2^{-2j\log \left( \frac{n-1}{j}\right) }+\sum _{j=\lceil \sqrt{n-1}\rceil +1}^{\lfloor \frac{n}{2}\rfloor }2^{-2j}\nonumber \\&\ll \sum _{j=B+1}^{\lceil \sqrt{n-1}\rceil }2^{-j\log \left( n-1\right) }+2^{-2\sqrt{n-1}}\ll (n-1)^{(B+1)\log 2}+2^{-2\sqrt{n-1}}. \end{aligned}$$

Thus, in total by combining (5.8), (5.9) and (5.10) we achieve

$$\begin{aligned} \Sigma _{2}(w,B,n)&\ll _{B,w,g}n^{|w|}\left( (n-1)^{-2\log B}+(n-1)^{(B+1)\log 2}+2^{-2\sqrt{n-1}}\right) \\&\ll _{B,w,g}n^{|w|-2\log B} \end{aligned}$$

as $n\rightarrow \infty$*. This proves the second statement of Theorem*1.9. $\square$

### Proof of Theorem 1.1

#### Proof of Theorem 1.1

We are given $M\in \mathbf {N}$ and we choose $B\in \mathbf {N}$ such that

$$\begin{aligned} 2\log B\ge M+|w|\ge M. \end{aligned}$$

We first take care of the term $\zeta (2g-2;n)^{-1}$ appearing in Corollary 1.6. Corollary 5.1 shows that

$$\begin{aligned} \zeta (2g-2;n)&=\sum _{(\rho ,W)\in \Omega (B;n)}\frac{1}{(\dim W)^{2g-2}}+O_{g,B}\left( \frac{1}{n}\left( n^{-2\log B}\right) \right) \\&=\sum _{\begin{array}{c} \mu ,\nu \\ \ell (\mu ),\ell (\nu )\le B,\mu _{1},\nu _{1}\le B^{2} \end{array} }\frac{1}{D_{[\mu ,\nu ]}(n)^{2g-2}}+O_{g,B}\left( n^{-M-1}\right) . \end{aligned}$$

Now as the sum is over a fixed finite set, Corollary 2.3 implies that for $n>2B^{3}$ we have

5.11 $$\begin{aligned} \sum _{(\mu ,\nu )\in \Omega (B)}\frac{1}{D_{[\mu ,\nu ]}(n)^{2g-2}}=F_{g,B}(n) \end{aligned}$$

for $F_{g,B}\in \mathbf {Q}(t)$ with only possible poles in $[-2B^{3},2B^{3}]$ and no zeros in $(2B^{3},\infty )$. From Theorem 1.8, $\lim _{n\rightarrow \infty }F_{g,B}(n)=1$; this can also be seen directly from (5.11). Therefore, we have

5.12 $$\begin{aligned} \zeta (2g-2;n)^{-1}= & {} \frac{1}{F_{g,B}(n)}\left( 1+O_{g,B}\left( F_{g,B}(n)^{-1}n^{-M-1}\right) \right) ^{-1}\nonumber \\= & {} \frac{1}{F_{g,B}(n)}+O_{g}\left( n^{-M-1}\right) \end{aligned}$$

as $$n\rightarrow \infty $$.

By Theorem 3.2, Theorem 1.9 and Corollary 1.6, we have

5.13 $$\begin{aligned} \mathbb {E}_{g,n}[\mathrm {tr} _{\gamma }]&=\frac{1}{\zeta (2g-2;n)}\left( Q_{B,w}(n)+O_{w,g}\left( n^{|w|-2\log B}\right) \right) \nonumber \\&=\frac{1}{\zeta (2g-2;n)}\left( Q_{B,w}(n)+O_{w,g}\left( n^{-M}\right) \right) \end{aligned}$$

as $n\rightarrow \infty$, where $Q_{w,B}\in \mathbf {Q}(t)$. Combining (5.12) with (5.13) gives as $n\rightarrow \infty$

5.14 $$\begin{aligned} \mathbb {E}_{g,n}[\mathrm {tr} _{\gamma }]&=\left( \frac{1}{F_{g,B}(n)}+O_{g}(\left( n^{-M-1}\right) \right) \left( Q_{w,B}(n)+O_{g,w}\left( n^{-M}\right) \right) \nonumber \\&=\frac{Q_{w,B}(n)}{F_{g,B}(n)}+O_{g}\left( Q_{w,B}(n)n^{-M-1}\right) +O_{g,w}\left( n^{-M}\right) . \end{aligned}$$

Using $F_{g,B}(n)\rightarrow 1$ as $n\rightarrow \infty$ again, and $\mathbb {E}_{g,n}[\mathrm {tr} _{\gamma }]\le n$, we obtain $Q_{w,B}(n)=O_{g,w}(n)$ as $n\rightarrow \infty$, and hence, we bootstrap (5.14) to

$$\begin{aligned} \mathbb {E}_{g,n}[\mathrm {tr} _{\gamma }]=\frac{Q_{w,B}(n)}{F_{g,B}(n)}+O_{g,w}\left( n^{-M}\right) . \end{aligned}$$

This completes the proof; $\frac{Q_{w,B}(n)}{F_{g,B}(n)}$ is *O*(*n*) and can be expanded as a Laurent polynomial as in (1.4) up to order $O\left( n^{-M}\right)$. Moreover, it is clear that the Laurent polynomials arising from different *M* must be coherent, i.e., arise from a fixed infinite sequence $a_{-1}(\gamma ),a_{0}(\gamma ),a_{1}(\gamma )\ldots$ of rational numbers. $\quad \square$

## Calculation of Example 1.2

Let $w=[a,b]\in \mathrm {\mathbf {F}} _{4}=\langle a,b,c,d\rangle$ and let $\gamma$ denote the projection of *w* to $\Gamma _{2}=\langle \,a,b,c,d\,|\,[a,b][c,d]\,\rangle .$ We will calculate the asymptotic expansion of $\mathbb {E}_{2,n}[\mathrm {tr} _{\gamma }]$ up to and including order $n^{-4}$.

Corollary 5.1 and Corollary 1.10 tell us that for some large enough fixed $B\in \mathbf {N}$ we have

A.1 $$\begin{aligned} \zeta (2;n)&=\sum _{(\rho ,W)\in \Omega (B;n)}\frac{1}{(\dim W)^{2}}+O\left( \frac{1}{n^{4}}\right) , \end{aligned}$$

A.2 $$\begin{aligned} \mathbb {E}_{2,n}[\mathrm {tr} _{\gamma }]=&\zeta (2;n)^{-1}\sum _{\begin{array}{c} {{({\rho ,W)\in }\Omega (B;n)}} \end{array} }(\dim W){\mathcal {I}}(w,\rho )+O\left( \frac{1}{n^{5}}\right) . \end{aligned}$$

as $n\rightarrow \infty$. Here we identify the set $\Omega (B;n)$ with pairs $(\mu ,\nu )$ of YDs as in §§3.1.

The first step is to calculate the first few terms of the asymptotic expansion of $\zeta (2;n)$. Unless the family $(\rho _{n}^{[\mu ,\nu ]},W_{n}^{[\mu ,\nu ]})$ is trivial ($\mu =\nu =\emptyset$, $\dim (W_{n}^{[\mu ,\nu ]})=1$), standard ($\mu =\square$, $\nu =\emptyset$, $\dim (W_{n}^{[\mu ,\nu ]})=n)$, or dual to standard ($\mu =\emptyset ,\nu =\square$, $\dim (W_{n}^{[\mu ,\nu ]})=n$) we have $\dim (W_{n}^{[\mu ,\nu ]})\gg n^{2}$ as $n\rightarrow \infty$. Since the set $\Omega (B;n)$ is finite, these observations give

A.3 $$\begin{aligned} \zeta (2;n)&=1+\frac{2}{n^{2}}+O\left( \frac{1}{n^{4}}\right) ,\quad \zeta (2;n)^{-1}=1-\frac{2}{n^{2}}+O\left( \frac{1}{n^{4}}\right) \end{aligned}$$

as $$n\rightarrow \infty $$.

Now, to use (A.2) we must calculate each

A.4 $$\begin{aligned}&{\mathcal {I}}(w,\rho _{n}^{[\mu ,\nu ]}) \nonumber \\&\quad =\int _{(A,B,C,D)\in \mathsf {SU}(n)^{4}}\mathrm {tr} ([A,B])\overline{s_{[\mu ,\nu ]}([A,B][C,D])}d\mu _{\mathsf {SU}(n)^{4}}^{\mathrm {Haar}}((A,B,C,D)). \end{aligned}$$

appearing therein. We use the following two formulas:

$$\begin{aligned}&\int _{D\in \mathsf {SU}(n)}\overline{s_{[\mu ,\nu ]}([A,B][C,D])}d\mu _{\mathsf {SU}(n)}^{\mathrm {Haar}}(D) =\frac{1}{\dim W_{n}^{[\mu ,\nu ]}}\overline{s_{[\mu ,\nu ]}([A,B]C)}\overline{s_{[\mu ,\nu ]}(C^{-1})},\\&\int _{C\in \mathsf {SU}(n)}\overline{s_{[\mu ,\nu ]}([A,B]C)}\overline{s_{[\mu ,\nu ]}(C^{-1})}d\mu _{\mathsf {SU}(n)}^{\mathrm {Haar}}(C) =\frac{1}{\dim W_{n}^{[\mu ,\nu ]}}\overline{s_{[\mu ,\nu ]}([A,B])} \end{aligned}$$

The first of these follows from [BtD95, Prop. 4.2] and the second by orthogonality of matrix coefficients. Using these in (A.4) to integrate out *C* and *D* gives

A.5 $$\begin{aligned} {\mathcal {I}}(w,\rho _{n}^{[\mu ,\nu ]})=&\frac{1}{(\dim W_{n}^{[\mu ,\nu ]})^{2}}\nonumber \\&\int _{(A,B)\in \mathsf {SU}(n)^{2}}\mathrm {tr} ([A,B])\overline{s_{[\mu ,\nu ]}([A,B])}d\mu _{\mathsf {SU}(n)^{2}}^{\mathrm {Haar}}((A,B)). \end{aligned}$$

It follows from Pieri’s rule [FH91, (A.7)] that

$$\begin{aligned} \mathrm {tr} ([A,B])\overline{s_{[\mu ,\nu ]}([A,B])}=\sum _{\mu ',\nu '}\overline{s_{[\mu ',\nu ']}([A,B])} \end{aligned}$$

where $\mu '$ and $\nu '$ are YDs formed by either removing one box from $\mu$ (leaving $\nu$ unaltered) or adding a box to $\nu$ (leaving $\mu$ unaltered). Finally, using a formula of Frobenius [Fro96] to obtain

$$\begin{aligned} \int _{(A,B)\in \mathsf {SU}(n)^{2}}\overline{s_{[\mu ',\nu ']}([A,B])}d\mu _{\mathsf {SU}(n)^{2}}^{\mathrm {Haar}}((A,B))=\frac{1}{\dim W_{n}^{[\mu ',\nu ']}}, \end{aligned}$$

we obtain from (A.5) that

A.6 $$\begin{aligned} (\dim W_{n}^{[\mu ,\nu ]}){\mathcal {I}}(w,\rho _{n}^{[\mu ,\nu ]})=\frac{1}{\dim W_{n}^{[\mu ,\nu ]}}\sum _{\mu ',\nu '}\frac{1}{\dim W_{n}^{[\mu ',\nu ']}}. \end{aligned}$$

Below we list the contributions from (A.6) to the sum $$\sum _{\begin{array}{c} {{({\rho ,W)\in }\Omega (B;n)}} \end{array} }(\dim W){\mathcal {I}}(w,\rho )$$ from each quadruple $(\mu ,\nu ,\mu ',\nu ')$ whose contributions are not $O(n^{-5})$. In the following, we use $\dim W_{n}^{[(1),(1)]}=(n+1)(n-1)$, $W_{n}^{[(2),\emptyset ]}=\dim W_{n}^{[\emptyset ,(2)]}=\frac{n(n+1)}{2}$, $\dim W_{n}^{[(1,1),\emptyset ]}=W_{n}^{[\emptyset ,(1,1)]}=\frac{n(n-1)}{2}$.

**Table:**

$(\mu ,\nu ,\mu ',\nu ')$	Contribution
$(\emptyset ,\emptyset ,\emptyset ,(1))$	$n^{-1}$
$((1),\emptyset ,\emptyset ,\emptyset )$	$n^{-1}$
$((1),\emptyset ,(1),(1))$	$\frac{1}{n(n+1)(n-1)}$
$(\emptyset ,(1),\emptyset ,(2))$	$\frac{2}{n^{2}(n+1)}$
$(\emptyset ,(1),\emptyset ,(1,1))$	$\frac{2}{n^{2}(n-1)}$
$((2),\emptyset ,(1),\emptyset )$	$\frac{2}{n^{2}(n+1)}$
$((1,1),\emptyset ,(1),\emptyset )$	$\frac{2}{n^{2}(n-1)}$

Using all these, we get

$$\begin{aligned} \sum _{\begin{array}{c} {{({\rho ,W)\in }\Omega (B;n)}} \end{array} }(\dim W){\mathcal {I}}(w,\rho )&=\frac{2}{n}+\frac{9}{n(n^{2}-1)}+O\left( \frac{1}{n^{5}}\right) =\frac{2}{n}+\frac{9}{n^{3}}+O\left( \frac{1}{n^{5}}\right) . \end{aligned}$$

Combining this with (A.3) gives

$$\begin{aligned} \mathbb {E}_{2,n}[\mathrm {tr} _{\gamma }]=\left( 1-\frac{2}{n^{2}}+O\left( \frac{1}{n^{4}}\right) \right) \left( \frac{2}{n}+\frac{9}{n^{3}}+O\left( \frac{1}{n^{5}}\right) \right) =\frac{2}{n}+\frac{5}{n^{3}}+O\left( \frac{1}{n^{5}}\right) . \end{aligned}$$

## Footnotes

1 Simple means non-self-intersecting.

2 These families of representations already feature in physics literature related to the problems of this paper; they are called ‘composite representations’ by Gross and Taylor in [GT93].

3 Note that compact Lie groups include all finite groups.

4 In this paper, all Hilbert spaces appearing are finite-dimensional.

5 The heat kernel will only be involved for a short time before being dispensed of.

6 This is a straightforward application of the Weingarten calculus.

7 Admittedly, this is overkill. The full arguments needed to prove Theorem 1.9 are not required for Corollary 5.1: the entirety of $\hbox { \char "0A7}$4 is bypassed by (5.2).
